# Swapping the Positions in a Cross-Strand Lateral Ion-Pairing Interaction between Ammonium- and Carboxylate-Containing Residues in a β-Hairpin

**DOI:** 10.3390/molecules26051346

**Published:** 2021-03-03

**Authors:** Cheng-Hsin Huang, Tong Wai Wong, Chen-Hsu Yu, Jing-Yuan Chang, Shing-Jong Huang, Shou-Ling Huang, Richard P. Cheng

**Affiliations:** 1 Department of Chemistry, National Taiwan University, Taipei 10617, Taiwan; R07223208@ntu.edu.tw (C.-H.H.); R02223133@ntu.edu.tw (T.W.W.); R05223205@ntu.edu.tw (C.-H.Y.); R08223149@ntu.edu.tw (J.-Y.C.); 2 Instrumentation Center, National Taiwan University, Taipei 10617, Taiwan; shingjonghuang@ntu.edu.tw (S.-J.H.); shouling@ntu.edu.tw (S.-L.H.)

**Keywords:** ion-pairing interaction, side-chain length, charged amino acids, β-hairpin, peptide

## Abstract

Cross-strand lateral ion-pairing interactions are important for antiparallel β-sheet stability. Statistical studies suggested that swapping the position of cross-strand lateral residues should not significantly affect the interaction. Herein, we swapped the position of ammonium- and carboxylate-containing residues with different side-chain lengths in a cross-strand lateral ion-pairing interaction in a β-hairpin. The peptides were analyzed by 2D-NMR. The fraction folded population and folding free energy were derived from the chemical shift data. The ion-pairing interaction energy was derived using double mutant cycle analysis. The general trends for the fraction folded population and interaction energetics remained similar upon swapping the position of the interacting charged residues. The most stabilizing cross-strand interactions were between short residues, similar to the unswapped study. However, the fraction folded populations for most of the swapped peptides were higher compared to the corresponding unswapped peptides. Furthermore, subtle differences in the ion-pairing interaction energy upon swapping were observed, most likely due to the “unleveled” relative positioning of the interacting residues created by the inherent right-handed twist of the structure. These results should be useful for developing functional peptides that rely on lateral ion-pairing interactions across antiparallel β-strands.

## 1. Introduction

The β-sheet is an important protein secondary structure. About one-fourth of protein residues adopt a β-sheet conformation in protein structures [1,2,3]. Furthermore, β-sheets are also formed in amyloid fibrils involved in various diseases, including Alzheimer’s disease [4,5], Huntington’s disease [6], and Parkinson’s disease [7,8]. Therefore, understanding the folding energetics of β-sheets is scientifically important with potential therapeutic applications [9,10].

The side-chains of the closest residues on adjacent strands are on the same face of a β-sheet. This would enable cross strand lateral side-chain-side-chain interactions. Statistical analysis showed that oppositely charged residues are frequently observed across antiparallel β-sheets [11,12,13], suggesting that cross-strand interactions between oppositely charged residues may be important for β-sheet stability. Accordingly, the energetics of cross strand ion pairs have been measured in sheet-containing host systems, including the protein G B1 domain [14,15], the zinc finger domain [16], and β-hairpins [11,17,18,19,20,21,22,23]. For the protein G B1 domain, a cross strand lateral Glu44-Lys53 ion-pairing interaction increased the protein stability by 1.0 kcal/mol based on thermal denaturation studies [14]. For the zinc finger domain, cross strand ion-pairing interactions involving Asp were more stabilizing compared to those involving Glu based on competitive metal ion binding studies [16]. In particular, cross strand Lys3-Asp10 and Arg3-Asp10 interactions stabilized the system by 0.48 and 0.26 kcal/mol, respectively [16].

The effect of charged amino acid side-chain length on cross strand lateral ion-pairing interaction was investigated in hairpin peptides [22,23]. The negatively charged carboxylate-containing amino acids with different side-chain lengths were incorporated at the N-terminal strand guest site (position 4), whereas the ammonium-containing amino acids with different side-chain lengths were incorporated at the C-terminal strand guest site (position 9) [22]. The results showed that length matching was necessary to form a stabilizing interaction, i.e., the side-chain length of the carboxylate- and ammonium-containing residues were either both long or both short [22]. The long side-chains provided large hydrophobic surfaces to interact with one another. Alternatively, the short side-chains paid less side-chain entropic penalties to interact with one another.

Statistical analysis showed that cross strand lateral residue pairs in antiparallel β-sheets are symmetric [24], meaning that swapping the position of a pair of cross strand lateral residues (i.e., orientation) should not significantly affect the interaction. However, two different experimental studies showed that swapping the positions of an amino acid pair in antiparallel β-sheets changed the stability of the system [14,19]. For the protein G B1 domain, the cross strand Phe44-Thr53 interaction stabilized the protein by 0.19 kcal/mol, but the Thr44-Phe53 interaction destabilized the protein by 0.36 kcal/mol based on thermal denaturation studies [14]. In addition, the Ile44-Phe53 and Ile44-Thr53 interactions were non-identical compared to the corresponding swapped interactions, with a change in overall thermal stability of the system [14]. Similarly, swapping the oppositely charged residues in the cross strand Lys3-Glu12 ion pair in a hairpin peptide altered the fraction folded population of the system based on NMR data [19]. As such, it appears that the statistical studies and the experimental studies contradict one another. Herein, we report the effect of lateral ion-pair interactions in a β-hairpin with the positively charged ammonium-containing residue at the N-terminal strand guest site (position 4) and negatively charged carboxylate-containing residue at the C-terminal strand guest site (position 9), effectively swapping the positions of the oppositely charged residues in a previous study [22].

## 2. Results

### 2.1. Peptide Design and Synthesis

**Figure 1:**
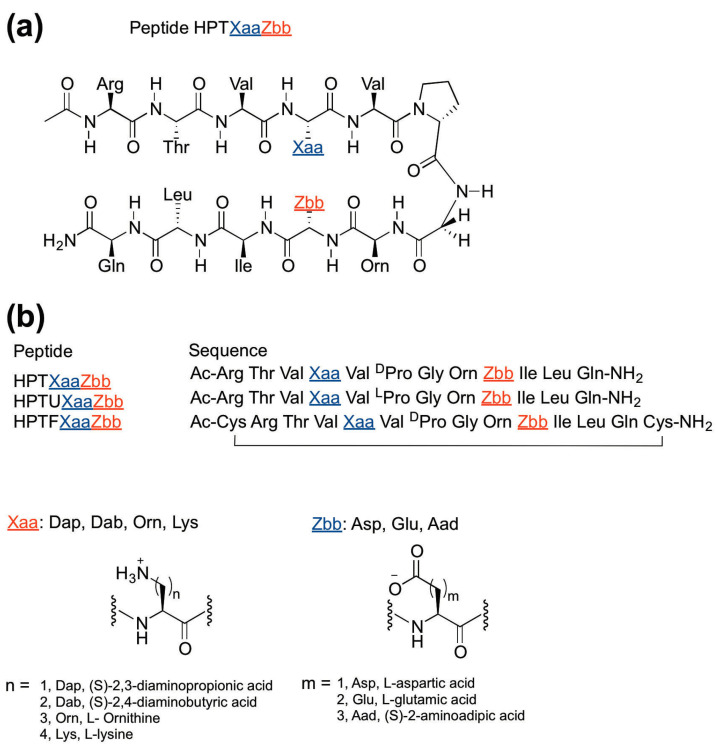
Design of peptides to study the effect of charged amino acid side-chain length upon swapping the charged amino acid positions in lateral ion-pairing interactions. (**a**) The chemical structure of the experimental HPTXaaZbb peptides; (**b**) The sequences of the experimental HPTXaaZbb peptides, the unfolded reference HPTUXaaZbb peptides, and the folded reference HPTFXaaZbb peptides.

The experimental HPTXaaZbb peptides were designed based on Gellman’s YKL peptide [11,25,26] and hairpin peptides in our previous studies [3,22,23] (Figure 1a). The Tyr2 (in peptide YKL) was replaced with Thr [22] because the aromatic side-chain of Tyr may interact diagonally with the residue at position 9 due to the right-handed twist [11,27,28]. An acetyl group and a carboxamide group was incorporated at the N- and C-termini, respectively, to remove the terminal charges, preventing unintended electrostatic interactions [17]. Non-hydrogen-bonded sites at positions 4 and 9 were chosen as guest sites [3,22,23], which were near the center of the strands to avoid end fraying near the termini and excessive folding near the turn [20,21]. Our previous study placed the negatively charged carboxylate-containing residues at position 4 and the positively charged ammonium- (or guanidinium-)-containing residues at position 9 [22,23]. To investigate the effect of charged amino acid side-chain length upon swapping the position of the charged residues in the lateral cross strand ion-pairing interaction, the positively charged ammonium-containing residues (Xaa = Lys, Orn, Dab, Dap) and negatively charged carboxylate-containing residues (Zbb = Aad, Glu, Asp) were incorporated at positions 4 and 9, respectively, to give the experimental HPTXaaZbb peptides (Figure 1b). The peptides were named with an “HPT” prefix, representing hairpin peptide with Thr at position 2, followed by the positively charged Xaa residue at position 4 and the negatively charged Zbb residue at position 9.

The fully folded reference peptides and the fully unfolded reference peptides were necessary to determine the fraction folded population of the experimental HPTXaaZbb peptides [26]. For the fully folded reference peptides, cysteine residues were added to both termini of the experimental HPTXaaZbb peptides to form intramolecular disulfide bonds to give macrocyclic peptides to serve as the fully folded reference peptides HPTFXaaZbb [3,11,22,23,25,26]. For the fully unfolded reference peptides, the DPro6 in the experimental HPTXaaZbb peptides was replaced with Pro to give the fully unfolded reference peptides HPTUXaaZbb [3,11,22,23,25,26], because Pro does not favor β-hairpin structures [11,26].

The peptides were synthesized by solid-phase peptide synthesis using Fmoc-based chemistry [29,30]. The disulfide bond in the folded reference HPTFXaaZbb peptides was formed via charcoal mediated air oxidation [31]. All peptides were purified by reverse-phase high-performance liquid chromatography (RP-HPLC) to higher than 95% purity and confirmed by matrix-assisted laser desorption ionization time-of-flight mass spectrometry (MALDI-TOF). Since the nuclear magnetic resonance (NMR) spectra (chemical shift and line width) of analogous hairpin peptides did not change with concentration (20 μM to 10 mM) [3,11,12,32], the peptides in this study (2.0−15.4 mM) should not aggregate in solution. Accordingly, the experimental data should reflect the intramolecular interactions with minimal interference from intermolecular interactions.

### 2.2. β-Hairpin Structure Characterization by NMR

The peptides were analyzed by ^1^H–^1^H homonuclear two-dimensional solution NMR spectroscopy, including double-quantum filtered-correlated spectroscopy (DQF-COSY) [33], total correlation spectroscopy (TOCSY) [34], and rotating-frame nuclear Overhauser effect spectroscopy (ROESY) [35] at 298 K. Sequence-specific assignment of all peptides was performed based on the TOCSY and ROESY spectra (Tables S1–S36) [36]. For a given Xaa4-Zbb9 pair, the chemical shift dispersion of the peptides followed the trend HPTFXaaZbb > HPTXaaZbb > HPTUXaaZbb (Tables S1–S36). Since the higher the fraction folded population, the higher the chemical shift dispersion [37], this trend is consistent with the intended designs of the peptides.

**Figure 2:**
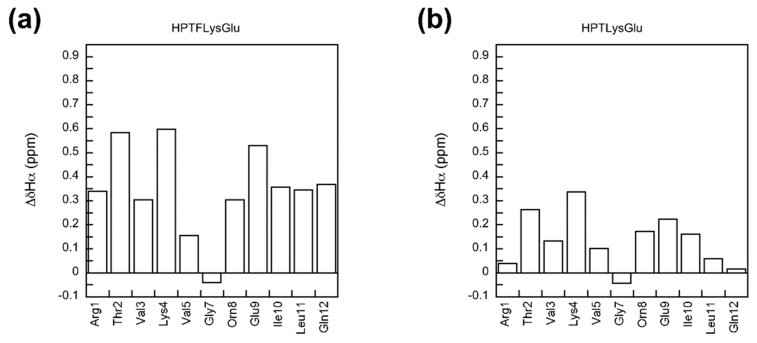
The chemical shift deviation (ΔδHα) for the residues in peptides HPTFLysGlu (**a**) and HPTLysGlu (**b**).

The β structure of the experimental and fully folded reference peptides was confirmed by the chemical shift deviations of the Hα signals, the ^3^J_HNα_ spin–spin coupling constants, and the NOE cross-peaks. The Hα chemical shift deviation (ΔδHα) is the difference between the Hα signal for the residue of interest and the corresponding random coil Hα signal [38]. In this study, the fully unfolded reference peptides were considered to be random coil [11,22,23,26]. A positive ΔδHα value suggests an extended β-sheet conformation [38,39]. The ΔδHα values of the residues Thr2 through Val5 and Orn8 through Leu11 for the experimental HPTXaaZbb peptides and the fully folded reference HPTFXaaZbb peptides were positive (Figure 2, Figures S1 and S2), suggesting an extended β-strand conformation for these residues. This is consistent with the intended design. In general, the ΔδHα values for the residues in the strand regions (residues 2–5 and residues 8–11) of the fully folded reference peptides were more positive compared to those for the corresponding experimental peptides (Figure 2, Figures S1 and S2), suggesting that the fully folded reference peptides were more well folded than the corresponding experimental peptides. The ΔδHα values of the terminal residues Arg1 and Gln12 for the experimental peptides were near zero (Figure 2, Figures S1 and S2), most likely due to end fraying effects [21]. The ΔδHα values for Gly7 were negative or mostly close to zero (Figure 2, Figures S1 and S2), consistent with turn formation [12].

The DQF-COSY spectra were used to determine the ^3^J_HNα_ spin–spin coupling constants for each residue in the peptides (Tables S37–S45) [33,40]. The ^3^J_HNα_ coupling constants of the residues in the fully folded reference HPTFXaaZbb peptides showed values higher than 7 Hz (Tables S43–S45), consistent with a β-hairpin structure [36,41]. The experimental HPTXaaZbb peptides also exhibited ^3^J_HNα_ coupling constants higher than 7 Hz, but slightly lower ^3^J_HNα_ values compared to those for the fully folded reference HPTFXaaZbb peptides (Tables S37–S39 and S43–S45). This suggested that the experimental HPTXaaZbb peptides may not be as well folded as the fully folded reference HPTFXaaZbb peptides. For the unfolded reference HPTUXaaZbb peptides, some residues exhibited ^3^J_HNα_ values near or less than 7 Hz (Tables S40–S42), suggesting that these peptides may not be as well folded as the experimental HPTXaaZbb peptides or the fully folded reference HPTFXaaZbb peptides.

The NOE cross-peaks in the ROESY spectra included sequential, intra-residues, medium-range, and long-range NOEs with a number of cross strand Hα–Hα, Hα–HN, HN–HN correlations (Figures S3–S50). All sequential Hα–HN NOE correlations in every strand for all peptides were observed (Figures S39–S50), consistent with β-strand formation [42,43]. In addition, the lack of d_αN_(*i*, *i* + n) (*n* = 2,3,4) and d_NN_(*i*, *i* + n) (*n* = 1, 2) patterns rules out the formation of other secondary structures (Figures S39–S50) [42,43]. A network of cross strand side-chain-side-chain NOEs between residues on the two β-strands was observed for the experimental peptides HPTXaaZbb and fully folded reference peptides HPTFXaaZbb (Figures S3−S38), consistent with β-hairpin formation for these peptides. Long-range NOE cross-peaks between Thr2 and Xaa9 were observed for most of the experimental HPTXaaZbb peptides and fully folded reference HPTFXaaZbb peptides (Figures S3−S38), consistent with a right-handed twist [11,27,28]. The number of cross-peaks in the ROESY spectra followed the general trend HPTFXaaZbb > HPTXaaZbb > HPTUXaaZbb (Figures S3−S38), consistent with the intended fraction folded population for our designs [3,22,23].

### 2.3. Fraction Folded Population and ΔG_fold_

The fraction folded population and folding free energy (ΔG_fold_) of each residue on the experimental peptides were derived from the Hα chemical shift deviation data (Figures S51 and S52). The residues close to the termini suffered from the end fraying effects [3,21,22,23]. The residues next to the turn were intrinsically highly folded due to proximity to the turn residues. Therefore, the residues near the center of the strands (positions 2, 3, 9, 10) were used to derive the fraction folded population and ΔG_fold_ for each peptide (Table 1 and Table 2) [3,11,20,22,23]. Both hydrogen-bonded sites (positions 3 and 10) and non-hydrogen-bonded sites (positions 2 and 9) were included [3,11,20,22,23]. Since the fraction folded population and the folding free energy showed the same trends (i.e., the more negative the folding free energy, the higher the fraction folded population), further discussion will only focus on the fraction folded data.

The fraction folded populations for the peptides were between 35% and 72%, and the standard deviations were within 5% (Table 1). Peptides HPTDapAsp and HPTDapGlu, containing the shortest positively charged residue Dap, exhibited exceptionally high fraction folded populations. In particular, HPTDapGlu exhibited the highest fraction folded population among all the HPTXaaZbb peptides. In contrast, HPTDapAad exhibited the least fraction folded population.

The fraction folded population of the HPTXaaAsp peptides followed the trend HPTDapAsp > HPTDabAsp ~ HPTOrnAsp > HPTLysAsp. Similarly, the fraction folded population of the HPTXaaGlu peptides followed the trend HPTDapGlu > HPTDabGlu ~ HPTOrnGlu ~ HPTLysGlu. However, the fraction folded population of the HPTXaaAad peptides followed the trend HPTDapAad < HPTDabAad < HPTOrnAad > HPTLysAad. If one disregards HPTDapAad and HPTDabAad, the fraction folded population of the HPTXaaZbb peptides for a given negatively charged residue Zbb9 generally decreased upon increasing the side-chain length of the positively charged residue Xaa4.

The fraction folded population of the HPTDapZbb peptides followed the trend HPTDapAsp < HPTDapGlu > HPTDapAad. The fraction folded population of the HPTDabZbb peptides followed the trend HPTDabAsp ~ HPTDabGlu < HPTDabAad. Similarly, the fraction folded population of the HPTOrnZbb peptides followed the trend HPTOrnAsp ~ HPTOrnGlu < HPTOrnAad. The fraction folded population of the HPTLysZbb peptides followed the trend HPTLysAsp ~ HPTLysGlu < HPTLysAad. Again, if one disregards HPTDapAad, the fraction folded population of the HPTXaaZbb peptides for a given positively charged residue Xaa4 generally increased with increasing side-chain length of the negatively charged residue Zbb9.

### 2.4. Lateral Cross Strand Xaa-Zbb Interactions

Double mutant cycle analysis was performed to derive the interaction free energy (ΔG_int_) for each lateral Xaa4-Zbb9 interaction (Table 3) [44,45]. For the reference peptides with minimal cross strand interaction, Ala was incorporated at position 4, position 9, or both positions 4 and 9 simultaneously because of the small side-chain of Ala [22,23,44]. The difference in folding energetics between peptides HPTXaaZbb and HPTAlaAla [22] would reflect the effect of simultaneously incorporating Xaa at position 4 and Zbb at position 9. This energy difference would include the effect of incorporating the Xaa residue and Zbb residue individually at positions 4 and 9, respectively, and the interaction between Xaa4 and Zbb9. Therefore, the effect of individually incorporating Xaa and Zbb would need to be considered to derive the Xaa4-Zbb9 interaction energy. The difference in folding energetics between peptides HPTXaaAla [3] and HPTAlaAla [22] would represent the effect of only incorporating Xaa at position 4. Similarly, the difference in folding energetics between peptides HPTAlaZbb [3] and HPTAlaAla [22] would represent the effect of only incorporating Zbb at position 9. The Xaa4-Zbb9 interaction energy (ΔG_int_) was determined from the folding energetics for the peptides HPTXaaZbb, HPTXaaAla [3], HPTAlaZbb [3], and HPTAlaAla [22] using Equation (7) (Table 3).

All of the cross strand lateral Xaa4-Zbb9 ion-pairing interactions were apparently stabilizing (Table 3). For the HPTXaaAsp peptides, the Xaa4-Asp9 interaction energy followed the trend Dap < Dab < Orn < Lys. Similarly, for the HPTXaaGlu peptides, the Xaa4-Glu9 interaction energy followed the trend Dap < Dab ~ Orn ~ Lys. For the HPTXaaAad peptides, the Xaa4-Aad9 interaction energy followed the trend Dap > Dab ~ Orn < Lys. If one disregards the Dap4-Aad9 interaction, the Xaa4-Zbb9 interaction generally becomes more stabilizing with decreasing Xaa4 side-chain length for a given Zbb9. Interestingly, the Dap4-Asp9 and Dap4-Glu9 interactions were the most stabilizing, providing more than 1 kcal/mol stabilization (Table 3). In contrast, the Dap4-Aad9 interaction provided the least stabilization, being essentially nonexistent. This showed that interaction between oppositely charged residues with short side-chains form stabilizing lateral cross strand ion-pairing interactions.

## 3. Discussion

The effect of side-chain length on lateral cross strand ion-pairing interactions between ammonium- and carboxylate-containing amino acids upon swapping the position of the charged amino acids was investigated. The fraction folded population for the HPTXaaZbb peptides was between 35% and 72% (Table 1). The extensive range of fraction folded population of the HPTXaaZbb peptides can be rationalized by the individual effects of the side-chain length of the ammonium- and carboxylate-containing at positions 4 and 9 on the hairpin formation, respectively, and the lateral cross strand Xaa4-Zbb9 interaction. In general, the fraction folded population of the HPTXaaZbb peptides for a given positively charged residue Xaa4 increased with increasing side-chain length of the negatively charged residue Zbb9 (except for peptide HPTDapAad; vide supra) (Table 1). This is consistent with the increased fraction folded hairpin population upon increasing the negatively charged residue side-chain length at position 9 for the HPTAlaZbb peptides [3]. In general, the fraction folded population of the HPTXaaZbb peptides for a given negatively charged residue Zbb9 decreased upon increasing the side-chain length of the positively charged residue Xaa4 (except for peptides HPTDapAad and HPTDabAad; vide supra) (Table 1). However, the fraction folded hairpin population for the HPTXaaAla peptides increased upon increasing the positively charged residue side-chain length at position 4 [3], suggesting the presence of cross strand Xaa4-Zbb9 interactions.

The two peptides with the highest fraction folded populations were HPTDapGlu (72 ± 3%) and HPTDapAsp (63 ± 1%). Similarly, the peptides with the same interacting residues, but the positions unswapped also exhibited the highest fraction folded populations in our previous study on HPTZbbXaa peptides (HPTGluDap: 63 ± 2%; HPTAspDap: 55 ± 3%) [22]. Nonetheless, the fraction folded populations of the HPTXaaZbb peptides with the charged residues swapped in this study (Table 1) were consistently higher compared to the corresponding unswapped HPTZbbXaa peptides in our previous study [22]. This is consistent with the higher fraction folded hairpin population for the HPTXaaAla peptides with the positively charged residue (Xaa) at position 4 compared to the corresponding HPTAlaXaa peptides with the positively charged residues (Xaa) at position 9 [3]. The change in fraction folded population upon swapping the residues in an interacting pair was consistent with studies on the protein G B1 domain [14] and a different hairpin system [19], which both showed a change in the stability of the host system upon swapping the position of interacting residues.

The largest difference in fraction folded population upon swapping was between the unswapped peptide HPTAadDab (26 ± 2%) [22] and the corresponding swapped peptide HPTDabAad (50 ± 1%). To gain further insight into this difference in the fraction folded population upon swapping, side-chain conformational analysis was performed on these two peptides by molecular mechanics calculations. The initial model was generated based on the solution structure of an analog of the parent YKL peptide (pdb code 1JY9 [46]). All possible combinations of low-energy side-chain dihedral angles (χ) for Aad and Dab were investigated. A combined total of 2916 conformations were minimized. The lowest energy conformation for the unswapped HPTAadDab exhibited higher energy (less negative energy, i.e., less stable) compared to that for the swapped HPTDabAad (Table 4), consistent with the fraction folded population for the two peptides. Conformations within 4 kcal/mol of the lowest energy conformer for each peptide were then examined (i.e., low-energy conformations, Table 4) because room temperature can provide up to 4 kcal/mol of thermal energy. All but one low-energy conformation exhibited salt bridges between the charged residues at positions 4 and 9. There were more low-energy conformations for the unswapped HPTAadDab compared to the swapped HPTDabAad. The energy reflects the enthalpic component of the conformation, whereas the number of low-energy conformations reflects the entropic component of the folded form of the peptide. The side-chain conformational entropy contribution of the residues at positions 4 and 9 to the free energy of the folded form for the two peptides was calculated based on the Boltzmann distribution of the various low-energy conformations (Table 4). The more negative −TS reflected the higher side-chain conformational entropy in the folded form for the unswapped HPTAadDab compared to the swapped HPTDabAad, despite involving the same two potentially interacting residues. The conformation of the low-energy conformers was examined in detail. Each χ_1_ dihedral was divided into three categories: gauche− (60°, g−), trans (180°, t), and gauche+ (300°, g+) [47,48]. The combination of the χ_1_ dihedrals was represented in parentheses (Table 4, Figures S53 and S54), showing the conformation for the residue at position 4 followed by the conformation for the residue at position 9. For example, a conformation with t at position 4 and g+ at position 9 would be designated (t, g+). For the unswapped HPTAadDab, 8 of the 9 possible combinations were present (Table 4 and Figure S53), whereas only 4 of the 9 possible combinations were observed for the swapped HPTDabAad (Table 4 and Figure S54). Importantly, the majority of the low-energy conformations did not involve g− (for χ_1_) at either position in either peptide (68% for HPTAadDab, and 94% for HPTDabAad). This is most likely because the g− conformation is higher in energy compared to t and g+ [47,48], and the g− conformation would inherently point the side-chain away from the neighboring strand.

**Figure 3:**
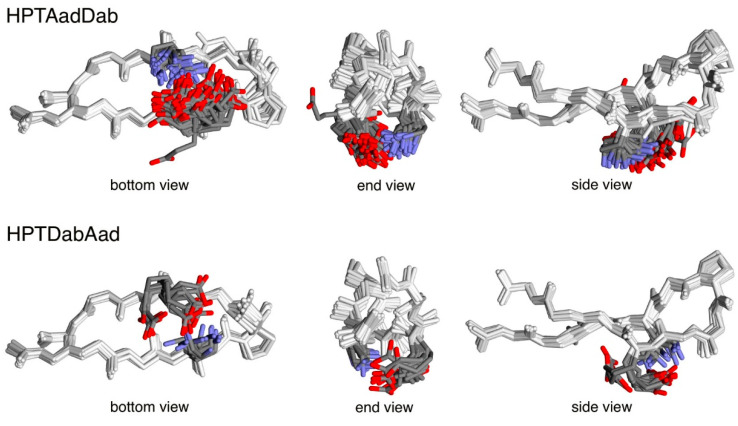
The low-energy conformations from molecular mechanics calculations for peptides HPTAadDab and HPTDabAad. The backbone and DPro side-chain are shown in white. The residues at positions 4 and 9 are colored according to element: carbon in gray, oxygen in red, and nitrogen in blue. The other side-chains and all hydrogen atoms are omitted for clarity.

**Figure 4:**
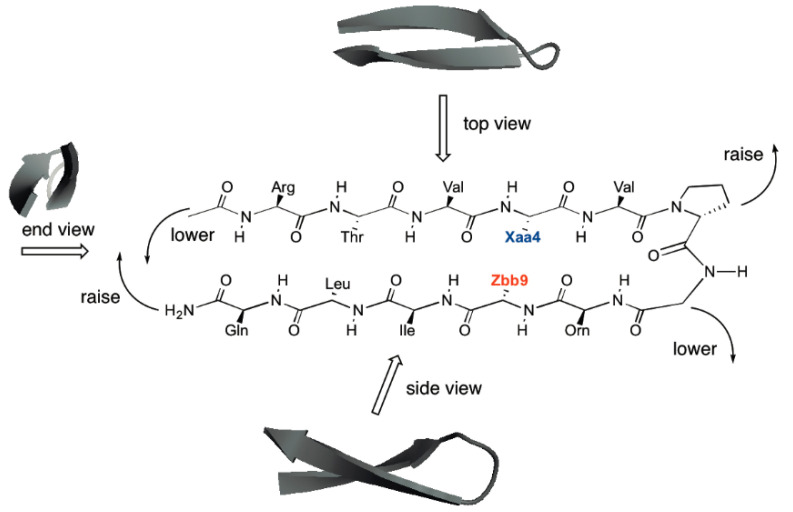
The chemical structure of HPTXaaZbb shown in perspective view, showing the consequence of the right-handed twist on a flat structure. The cartoon ribbon representation was generated from the solution structure of an analog of the parent YKL peptide using PyMOL (pdb code 1JY9 [46]).

More low-energy conformations were observed for the unswapped Aad4-Dab9 interaction compared to the swapped Dab9-Aad9 interaction (Table 4 and Figure 3). Apparently, the right-handed twist of the hairpin structure [11] raised the residue at position 4 and lowered the residue at position 9 (Figure 3 and Figure 4). This “unleveled” relative positioning of the interacting residues resulted in more proper length matching for the Aad4-Dab9 interaction, enabling more low-energy conformations with the Aad4-Dab9 interaction and a higher proportion of the g− conformation in χ_1_ (21 in 65, or 32%). For the swapped Dab4-Aad9 interaction, the unleveled relative positioning exacerbated the length difference between Dab4 and Aad9, leading to less low-energy conformations with the Dab4-Aad9 interaction and a relatively low proportion of the g− conformation in χ_1_ (1 in 16, or 6%). This unleveled positioning created by the right-handed twist appeared to be one of the factors giving rise to the difference between the unswapped and swapped peptides.

The energetic contribution of lateral cross strand Xaa4-Zbb9 interactions to hairpin formation was determined by double mutant cycle analysis (Table 3). For the HPTXaaZbb swapped peptides in this study, the Dap4-Glu9 and Dap4-Asp9 interactions were most stabilizing (Table 3). Similarly, the Glu4-Dap9 and Asp4-Dap9 interactions were the most stabilizing in our previous study on the original unswapped HPTZbbXaa peptides [22]. This may be because shorter amino acids such as Dap and Asp are conformationally less flexible compared to amino acids with longer side-chains, leading to higher stabilizing lateral cross strand ion-pairing interactions due to less entropic penalty [22]. The mean conformational entropic penalty of one side-chain rotatable bond upon folding is 0.5 kcal/mol [50].

There is a dramatic decrease in fraction folded population and stabilizing side-chain interaction for peptides HPTDapAsp and HPTDapGlu upon increasing the Dap side-chain length by just one methylene to Dab (Table 1 and Table 3). Increasing the side-chain length of Dap by one methylene to Dab would increase the electron-donating characteristics, decrease the electron-withdrawing characteristics from the backbone functionality, and thus decrease the cationic charge density on the ammonium group. This decrease in cationic charge density would decrease the electrostatic interaction with the negatively charged Asp and Glu, resulting in the decreased fraction folded population for peptides HPTDabGlu and HPTDabAsp compared to peptides HPTDapGlu and HPTDapAsp, respectively (Table 1 and Figure 3).

Further lengthening the positively charged residue Dab in HPTDabGlu and HPTDabAsp did not alter the fraction folded population or the Xaa4-Zbb9 interaction as drastically as the change upon lengthening Dap to Dab. The longer side-chains are more flexible, and therefore, more energy would be needed to confine the side-chain conformation to enable the cross strand ion-pairing interaction. This would decrease the overall energetic contribution of the cross strand ion-pairing interaction for residues with longer side-chains. However, as the side-chain length of the positively charged residue Xaa4 increases, the fraction folded population of HPTXaaAla peptide increases [3]. As such, this increase in hairpin formation (due to the positively charged residue Xaa) compensates for the increase in the side-chain entropic penalty for the longer side-chains to form a cross strand ion-pairing interaction, leading to less drastic changes in the fraction folded population.

In general, the interaction free energy became less stabilizing with increasing side-chain length of the positively charged residue Xaa4 for a given negatively charged residue Zbb9 (except for Dap4-Aad9) in the swapped HPTXaaZbb peptides in this study (Table 3). The same general trend was also observed in our previous study on the original unswapped HPTZbbXaa peptides, but with less stabilizing lateral cross strand interactions between residues at positions 4 and 9 [22]. This difference in interaction energy could be due to the difference in the relative placement of the residues at position 9 on the C-terminal strand and position 4 on the N-terminal strand (Figure 4, vide infra), stemming from the inherent right-handed twist of sheet structures [28].

The Xaa4-Asp9 interactions were more stabilizing compared to the corresponding Xaa4-Glu9 and Xaa4-Aad9 interactions for a given Xaa4 (Table 3). This is perhaps the result of the relative positioning of Xaa4 and Zbb9. The right-handed twist of the hairpin structure [11] lowers Zbb9 and raises Xaa4 (Figure 4). For Xaa4 to interact with Zbb9, the ammonium group on Xaa4 and the carboxylate group on Zbb9 need to be close to one another. Apparently, length matching is critical for lateral cross strand interactions [22,23]. Since the carboxylate group on Zbb9 is inherently longer than the ammonium group on Xaa4, the shorter Asp9 would be more well suited to interact with Xaa4 (especially shorter residues) compared to the longer Glu9 and Aad9 due to the “unleveled” relative positioning of the residues created by the right-handed twist (Figure 4). In comparison, interactions between the longer Glu9 (and Aad9) and Xaa4 would be weaker compared to the corresponding Xaa4-Asp9 interactions. Furthermore, the Xaa4-Asp9 interaction would be more stabilizing because of the need to pay less of an entropic penalty to confine the short Asp9 side-chain conformation to enable the Xaa4-Asp9 interaction compared to the longer Glu and Aad. For the original unswapped HPTZbbXaa peptides [22], there is no general trend among the Asp4-Xaa9, Glu4-Xaa9, and Aad4-Xaa9 interactions for a given Xaa9 residue. This may be because the unleveled relative positioning created by the right-handed twist facilitates the length matching, bringing the inherently shorter ammonium functionality closer to the inherently longer carboxylate functionality. These results are consistent with the studies on cross-strand interactions in the protein G B1 domain [14], showing that swapping the amino acid positions in lateral cross-strand interactions changed the interaction energy. Overall, our results suggest that there is an orientation preference for lateral cross strand interactions to stabilize sheet systems, despite the apparent symmetry of lateral cross strand interactions based on statistical studies [24].

The Dap4-Aad9 and Lys4-Aad9 interactions were less stabilizing compared to the Dab4-Aad9 and Orn4-Aad9 interactions if one disregards the error bars. The low stabilization of the Lys4-Aad9 interaction may be due to the conformationally more flexible Lys side-chain, leading to the need to pay a higher entropic penalty to confine the long Lys and Aad side-chains to enable the Lys4-Aad9 interaction. The low stabilization of the Dap4-Aad9 interaction may be due to the length discrepancy between Dap and Aad, which is further magnified by the unleveled relative placement of residues at positions 4 and 9 resulting from the right-handed twist. Importantly, the combination of unleveled relative placement of the interacting functional groups and difference in entropic penalty necessary to form the lateral cross-strand interaction resulted in the observed trends and the effects upon swapping interacting residues.

## 4. Materials and Methods

### 4.1. Peptide Synthesis

Peptides were synthesized by solid-phase peptide synthesis using Fmoc-based chemistry [29,30]. The disulfide bond in the Cys-containing HPTFXaaZbb peptides was formed via charcoal-mediated air oxidation [31]. All peptides were purified by reverse-phase high-performance liquid chromatography (RP-HPLC) (Waters, Milford, MA, USA) to higher than 95% purity. The identity of the peptides was confirmed by matrix-assisted laser desorption ionization time-of-flight mass spectrometry (MALDI-TOF) (Bruker, Billerica, MA, USA). More detailed procedures and peptide characterization data are provided in the Supplementary Materials.

### 4.2. Nuclear Magnetic Resonance Spectroscopy

Purified peptides were dissolved in H_2_O/D_2_O (9:1 ratio by volume) in the presence of 50 mM sodium deuterioacetate buffer (pH 5.5 uncorrected). Peptide concentrations were 2.0–15.4 mM. 2-Dimethyl-2-silapentane-5-sulfonate (DSS) was added to the sample as an internal reference. All NMR experiments were performed on a Brüker AVIII 800 MHz spectrometer (Bruker, Billerica, MA, USA). ^1^H-^1^H homonuclear phase-sensitive double-quantum filtered-correlated spectroscopy (DQF-COSY) [33], total correlation spectroscopy (TOCSY) [34], and rotating-frame nuclear Overhauser effect spectroscopy (ROESY) [35] experiments were performed by collecting 2048 point in f_2_ with 4–8 scans and 256–512 points in f_1_ at 298 K. Solvent suppression was achieved by the WATERGATE solvent suppression sequence [51]. TOCSY and ROESY experiments employed a spin locking field of 10 kHz. Mixing times of 60 and 200 ms were used for the TOCSY and ROESY experiment, respectively.

### 4.3. Chemical Shift Deviation

Sequence-specific assignments for all peptides were completed by using the 2D-NMR spectra (TOCSY and ROESY). The chemical shift deviation (ΔδHα) for each residue of the experimental peptide ΔδHα(exp)) and the folded reference peptide (ΔδHα(F)) was derived using Equations (1) and (2), respectively [38]. δHα(exp) is the chemical shift for the residue of interest on the experimental peptide, and δHα(U) is the chemical shift for the corresponding residue of interest on the fully unfolded reference peptide. δHα(F) is the chemical shift for the residue of interest on the fully folded reference peptide.

(1) ∆δHα(exp) = δHα(exp) − δHα(U)

(2) ∆δHα(F) = δHα(F) − δHα(U)

#### 4.4. J_HNα_ Spin–Spin Coupling Constant

The peak-to-peak separation in the absorptive (v_a_) and the dispersive (v_d_) spectra were measured to derive the *J* coupling constant. The v_a_ and v_d_ values were obtained using values on the f_2_ axis. Equation (3) was used to derive the coupling constants [40].

(3) $$J^{6}{\;-\;\upsilon }_{d}{}^{2}J^{4}+\left(-\frac{9}{4}{\;\upsilon }_{a}{}^{4}+\;\frac{3}{2}{\;\upsilon }_{a}{}^{2}{\;\upsilon }_{d}{}^{2}+\;\frac{3}{4}{\;\upsilon }_{d}{}^{4}\right)J^{2}+\;\frac{81}{64}{\;\upsilon }_{a}{}^{6}\;-\;\frac{9}{16}{\;\upsilon }_{a}{}^{4}\upsilon_{d}{}^{2}\;-\;\frac{21}{32}{\;\upsilon }_{a}{}^{2}\upsilon_{d}{}^{4}\;-\;\frac{1}{16}{\;\upsilon }_{d}{}^{6}\;-\;\frac{\upsilon_{d}{}^{8}}{64\upsilon_{a}{}^{2}}\;=\;0$$

### 4.5. Interproton Distance Determination via NOE Integration

The NOE cross-peaks for all peptides were assigned from the corresponding ROESY spectra. Integration was performed based on Gaussian peak modeling to obtain the intensity of cross-peaks (I). The distance between the β-hydrogen atoms on the proline side-chain (regardless of stereochemistry) was set as the standard (1.77 Å) to derive the interproton distance for the cross-peak of interest using Equation (4). The distances (r) were grouped into short (≤2.5 Å), medium (2.5~3.5 Å), and long (>3.5 Å) for the depictions in the Wüthrich diagrams (Figures S39–S50).

(4) $$r\;=\;1.77\times 10^{-10}\times {\left(\frac{I_{\mathrm{standard}}}{I_{\mathrm{NOE}}}\right)}^{\frac{1}{6}}$$

### 4.6. Fraction Folded Population and Folding Free Energy (ΔG_fold_)

The equilibrium constant between the unfolded and folded states of an experimental peptide is the ratio of the folded and unfolded populations. The fraction folded population for each residue was derived from the chemical shift data according to Equation (5). The folding free energy ΔG_fold_ for each residue was derived using Equation (6). The fraction folded population and folding free energy (ΔG_fold_) of the peptide was obtained by averaging the fraction folded population and ΔG_fold_, respectively, for the residues 2, 3, 9, and 10 [11,20,22,23].

(5) $$\mathrm{Fraction}\;\mathrm{Folded}\;\mathrm{Population}\;=\;\frac{\delta H\alpha \left(\exp\right)\;-\;\delta H\alpha \left(U\right)}{\delta H\alpha \left(F\right)\;-\;\delta H\alpha \left(U\right)}\times 100\%$$

(6) $$∆G_{\mathrm{fold}}\;=\;-\mathrm{RTln}\frac{\delta H\alpha \left(\exp\right)\;-\;\delta H\alpha \left(U\right)}{\delta H\alpha \left(F\right)\;-\;\delta H\alpha \left(\exp\right)}$$

### 4.7. Double Mutant Cycle Analysis

Double mutant cycle analysis [44] was performed to determine the interaction free energy (ΔG_int_) between charged residues Xaa4 and Zbb9 in the HPTXaaZbb peptides using Equation (7) [44,45]. This analysis accounted for the effect of each charged residue (individually) on strand stability using data from the corresponding Ala-containing peptides HPTXaaAla [3] and HPTAlaZbb [3] to determine the Xaa4-Zbb9 ion-pairing interaction exclusively (Table 3). The peptide with Ala incorporated at positions 4 and 9, HPTAlaAla [22], was used as the reference peptide.

(7) $${\Delta G}_{\mathrm{int}}=\left(∆G_{\mathrm{HPTXaaZbb}}-∆G_{\mathrm{HPTAlaAla}}\right)-\left(∆G_{\mathrm{HPTXaaAla}}-∆G_{\mathrm{HPTAlaAla}}\right)-\left(∆G_{\mathrm{HPTAlaZbb}}-∆G_{\mathrm{HPTAlaAla}}\right)$$

### 4.8. Side-Chain Conformational Analysis by Molecular Mechanics Calculations

The conformational analysis was performed using the program Discovery Studio 2.1 (Accelrys, CA, USA) on an IBM x3550M2 workstation (CPU: Dual Xeon E5530 2.4 GHz with Quad cores; RAM: 48 G) running the operating system CentOS 5.3. The models were created based on the solution structure of an analogous peptide of the parent YKL peptide [46] with various combinations of potential low-energy side-chain dihedrals. For each side-chain dihedral angle (χ) involving sp^3^ carbons, three possible low-energy staggered conformations were considered: gauche− (60°, g−), trans (180°, t), and gauche+ (300°, g+) [47,48]. For the dihedral angle involving the sp^2^ carboxylate carbon of Aad, six conformations were considered: 0°, 30°, 60°, 90°, 120°, and 150°. For each peptide (HPTAadDab and HPTDabAad), 1458 conformations were evaluated. Each conformation was minimized using the CFF forcefield. The nonbond radius of 99 Å, nonbond higher cutoff distance of 98 Å and nonbond lower cutoff distance of 97 Å were employed to perform the calculations with effectively no cutoffs. Distance dependent dielectric constant of 2 was used as the implicit solvent model. Minimization was performed by steepest descent and conjugate gradient protocols until convergence (converging slope was set to 0.1 kcal/(mol × Å). After minimization, each conformation was reexamined to remove duplicating conformations because minimization with different starting conformations occasionally resulted in the same final conformation. When the same conformation was obtained more than once, only the lowest energy conformation was considered in further analyses. The probability of conformation i at 298 K (p_i_) was calculated based on Boltzmann distribution using Equation (8), in which ε_i_ is the energy of conformation i, k_B_ is the Boltzmann constant, and T is the temperature (298 K). The entropic contribution to the folded form at 298 K was calculated using Equation (9).

(8) $$p_{i}\;=\;\frac{e^{\frac{-\varepsilon_{i}}{k_{B}T}}}{\sum_{j}e^{\frac{-\varepsilon_{j}}{k_{B}T}}}$$

(9) $$-\mathrm{TS}\;=\;-T\cdot \left(-k_{B}\right)\underset{i}{\sum }p_{i}\cdot \ln\left(p_{i}\right)$$

## 5. Conclusions

We investigated the effect of swapping the cross strand interacting charged amino acid positions in a β-hairpin from the original Zbb4-Xaa9 in a previous study (HPTZbbXaa peptides) to the swapped Xaa4-Zbb9 in this study (HPTXaaZbb peptides). The general trends for the fraction folded population, and side-chain interaction energetics remained similar upon swapping the position of potentially interacting charged residues. Nonetheless, the fraction folded populations for most of the swapped HPTXaaZbb peptides were higher compared to the corresponding original HPTZbbXaa peptides, consistent with the inherent effect of the positively charged Xaa residue on hairpin formation at the two different positions. The most stabilizing cross strand interactions were between short residues (Dap4-Asp9 and Dap4-Glu9) even after swapping the position of the charged residues. However, subtle differences were present, most likely due to the unleveled relative placement of the residues at positions 4 and 9 created by the right-handed twist of the sheet structure. These results should be useful for developing functional peptides that rely on lateral ion-pairing interactions across antiparallel β-strands.

## Figures and Tables

**Table 1:** The fraction folded population (%) for the HPTXaaZbb peptides ^1^.

Xaa4	Zbb9		
	Asp	Glu	Aad
Dap	63 ± 1	72 ± 3	35 ± 2
Dab	47 ± 5	45 ± 2	50 ± 1
Orn	46 ± 2	47 ± 2	57 ± 1
Lys	41 ± 2	44 ± 1	52 ± 2

^1^ Average value for residues 2, 3, 9, and 10.

**Table 2:** The folding free energy (ΔG_fold_, kcal/mol) for the HPTXaaZbb peptides ^1^.

Xaa4		Zbb9	
	Asp	Glu	Aad
Dap	−0.30 ± 0.03	−0.57 ± 0.08	0.36 ± 0.04
Dab	0.08 ± 0.03	0.12 ± 0.04	0.00 ± 0.03
Orn	0.10 ± 0.04	0.08 ± 0.06	−0.17 ± 0.02
Lys	0.23 ± 0.04	0.14 ± 0.03	−0.05 ± 0.04

^1^ Average value for residues 2, 3, 9, and 10.

**Table 3:** The Xaa4-Zbb9 ion-pairing interaction energy (ΔG_int_, kcal/mol) ^1^.

Xaa4	Zbb9		
	Asp	Glu	Aad
Dap	−1.09 ± 0.05	−1.10 ± 0.07	−0.07 ± 0.06
Dab	−0.54 ± 0.05	−0.23 ± 0.08	−0.27 ± 0.05
Orn	−0.40 ± 0.05	−0.17 ± 0.12	−0.32 ± 0.05
Lys	−0.24 ± 0.06	−0.13 ± 0.12 ^2^	−0.17 ± 0.11

^1^ Average value for residues 2, 3, 9, and 10. ^2^ Average value for residues 2, 3, and 10.

**Table 4:** Summary of the low-energy conformations from the side-chain conformational analysis of peptides HPTAadDab and HPTDabAad by molecular mechanics calculations.

Peptide	Lowest Energy Conformation Energy	Side-Chain Conformational Entropy Contribution	Conformations within 4 kcal/mol of the Lowest Energy Conformer		
	(kcal/mol)	−TS (kcal/mol)	No ^1^	Salt Bridge ^2^ (%)	Conformations, Number (Residue 4 χ_1_, Residue 9 χ_1_)
HPTAadDab	−384.4	−1.67	65	98	16 (g+, g+), 14 (t, g+) 13 (g+, t), 8 (g−, g+) 6 (g−, t), 5 (g+, g−) 2 (g+, g−), 1 (t, t)
HPTDabAad	−387.8	−1.05	16	100	7 (t, g+), 5 (g+, t), 3 (g+, g+), 1 (g−, g+)

^1^ The number of conformations within 4 kcal/mol of the lowest energy conformer for each peptide. ^2^ The percentage of conformations within 4 kcal/mol of the lowest energy conformer with an Aad–Dab salt bridge, which is a hydrogen-bonded ion pair [49].

## Data Availability

The data presented in this study are available in the Supplementary Materials. The raw data are available on request from the corresponding author.

## Supplementary Materials

The following are available online. Tables S1–S36: The 1H chemical shift assignments for the peptides. Tables S37–S45: The 3JNHα values of the peptides. Figure S1: The Hα chemical shift deviations for the residues in the experimental HPTXaaZbb peptides. Figure S2: The Hα chemical shift deviations for the residues in the fully folded reference HPTFXaaZbb peptides. Figures S3–S38: The NOEs observed involving the side-chains of the peptides. Figures S39–S50: Wüthrich diagrams of the backbone NOE connectivities involving the α-protons and amide protons for the peptides. Figure S51: The fraction folded of the residues in the peptides. Figure S52: The ΔGfold of the residues in the peptides. Figure S53: The low-energy conformations for peptide HPTAadDab. Figure S54: The low-energy conformations for peptide HPTDabAad. Detailed peptide synthesis procedures and peptide characterization data.

## Sample Availability

Samples of the compounds are not available from the authors.
