# Supplementary material for: Swapping the Positions in a Cross-Strand Lateral Ion-Pairing Interaction between Ammonium- and Carboxylate-Containing Residues in a β-Hairpin
Source: Molecules. 2021 Mar 3;26(5):1346. doi: 10.3390/molecules26051346 (PMC7961788; doi:10.3390/molecules26051346)
Supplement: Supplementary file 1 [file molecules-26-01346-s001.pdf]

Supplementary Materials

Swapping the Positions in a Cross Strand Lateral Ion Pairing Interaction between Ammonium- and Carboxylate-Containing Residues in a β-Hairpin

Cheng-Hsin Huang, Tong Wai Wong, Chen-Hsu Yu, Jing-Yuan Chang, Shing-Jong Huang, Shou-Ling Huang, Richard P. Cheng*

Tables

Tables S1~S36. The ^{1}H Chemical Shift Assignments for the Peptides S2~S19

Tables S37~S45. The $^{3}J_{NH^{\alpha}}$ Values of the Peptides S20~S24

Figures

Figure S1. The Hα chemical shift deviations for the residues in the experimental HPTXaaZbb peptides. S25

Figure S2. The Hα chemical shift deviations for the residues in the fully folded reference HPTFXaaZbb peptides. S26

Figures S3~S38. The NOEs observed involving the side chains of the peptides. S27~S38

Figures S39~S50. Wüthrich diagrams of the backbone NOE connectivities involving the α-protons and amide protons for the peptides. S39~S50

Figure S51. The fraction folded of the residues in the peptides. S51

Figure S52. The ΔG_{fold} of the residues in the peptides. S52

Figure S53. The low energy conformations for peptide HPTAadDab. S53

Figure S54. The low energy conformations for peptide HPTDabAad. S54

Material and Methods S55~S66

References S67

Table S1. The ^{1}H Chemical Shift Assignments for Peptide HPTDapAsp

Residue	HN	Hα	Hβ	Others
Ac-		2.028		
Arg1[b]	8.297	4.399	1.749, 1.832	Hγ: 1.607, 1.652; Hδ: 3.201; HNt: 7.195
Thr2[c]	8.280	4.724	4.023	Hγ: 1.092
Val3[d]	8.684	4.354	2.035	Hγ: 0.897
Dap4[a]	8.859	5.320	3.261, 3.371	
Val5[e]	8.920	4.595	2.011	Hγ: 0.916
^{D}Pro6		4.382	1.974, 2.350	Hγ: 2.061; Hδ: 3.821
Gly7	8.650	3.829, 4.005		
Orn8	7.946	4.566	1.779, 1.847	Hγ: 1.686; Hδ: 3.007; NHt: 7.603
Asp9	8.536	5.209	2.410, 2.545	
Ile10	8.670	4.353	1.848	Hγ: 1.114, 1.377, 0.870 (Me); Hδ: 0.811
Leu11	8.421	4.476	1.591	Hγ: 1.591; Hδ: 0.837, 0.884
Gln12[f]	8.559	4.329	1.926, 2.079	Hγ: 2.296, 2.322; HNt: 6.872, 7.399
NH_2	7.134, 7.659			

[a]Signal for the terminal HN not observed. [b]The assignments for the minor Arg1 spin system are 8.351(HN), 4.288(Hα), 1.757, 1.841(Hβ), 1.598, 1.662(Hγ), 3.200(Hδ); [c]Thr2 spin system are 8.250(HN), 4.412 (Hα), 4.211(Hβ), 1.194(Hγ); [d]Val3 spin system are 8.210(HN), 4.173(Hα), 2.082(Hβ), 0.930(Hγ); [e]Val5 spin system are 8.115(HN), 4.127(Hα), 1.880(Hβ), 0.896(Hγ); [f]Gln12 spin system are 8.247(HN), 4.281(Hα), 1.974, 2.124(Hβ), 2.363(Hγ).

Table S2. The ^{1}H Chemical Shift Assignments for Peptide HPTDabAsp

Residue	HN	Hα	Hβ	Others
Ac-		2.030		
Arg1	8.306	4.381	1.749, 1.836	Hγ: 1.612, 1.657; Hδ: 3.204; HNt: 7.195
Thr2	8.232	4.643	4.065	Hγ: 1.113
Val3[b]	8.519	4.252	2.015	Hγ: 0.893
Dab4[a]	8.633	*4.879*	2.986, 3.028	Hγ: 2.041, 2.112
Val5[c]	8.595	4.532	1.996	Hγ: 0.921
^{D}Pro6		4.413	1.978, 2.327	Hγ: 2.038, 2.084; Hδ: 3.812
Gly7[d]	8.608	3.911, 3.967		
Orn8	8.047	4.531	1.789, 1.867	Hγ: 1.706 ; Hδ: 3.011; NHt: 7.615
Asp9	8.529	4.917	2.493, 2.543	
Ile10	8.575	4.285	1.878	Hγ: 1.172, 1.394, 0.884 (Me); Hδ: 0.820
Leu11	8.399	4.418	1.597	Hγ: 1.597 Hδ: 0.849, 0.902
Gln12[e]	8.457	4.308	1.936, 2.092	Hγ: 2.316, 2.335; HNt: 6.877, 7.428
NH_2	7.120, 7.618			

[a]Signal for the terminal HN not observed. [b]The assignments for the minor Val3 spin system are 8.188(HN), 4.120(Hα), 2.051(Hβ), 0.915(Hγ); [c]Val5 spin system are 8.107(HN), 4.125(Hα), 1.868(Hβ), 0.890(Hγ); [d]Gly7 spin system are 8.608(HN), 3.943, 4.024(Hα); [e]Gln12 spin system are 8.247(HN), 4.390(Hα), 1.973, 2.120(Hβ), 2.365(Hγ).

Table S3. The ^{1}H Chemical Shift Assignments for Peptide HPTOrnAsp

Residue	HN	Hα	Hβ	Others
Ac-		2.042		
Arg1[b]	8.310	4.382	1.833, 1.745	Hγ: 1.655, 1.605; Hδ: 3.202; HNt: 7.205
Thr2	8.236	4.637	4.074	Hγ: 1.115
Val3[c]	8.505	4.243	2.009	Hγ: 0.892
Orn4[a]	8.455	4.766	1.728	Hγ: 1.636; Hδ: 2.925, 2.873
Val5[d]	8.654	4.533	2.004, 1.969	Hγ: 0.924
^{D}Pro6		4.403	2.094, 1.969	Hγ: 2.039, 1.983; Hδ: 2.331
Gly7[e]	8.525	3.944, 3.889		
Orn8	8.046	4.539	1.858, 1.789	Hγ: 1.699; Hδ: 3.010; NHt: 7.615
Asp9	8.510	4.851	2.549, 2.499	
Ile10	8.626	4.275	1.891	Hγ: 1.401, 1.201, 0.880 (Me); Hδ: 0.812
Leu11	8.399	4.412	1.627, 1.597	Hγ: 0.902; Hδ: 0.853
Gln12[f]	8.459	4.305	2.094, 1.941	Hγ: 2.327; HNt: 7.435
NH_2	7.620, 7.122			

[a]Signal for the terminal HN not observed. [b]The assignments for the minor Arg1 spin system are 8.334(HN), 4.348(Hα), 1.837(Hβ), 1.751, 1.633(Hγ); [c]Val3 spin system are 8.188(HN), 4.116(Hα), 2.048(Hβ), 0.916(Hγ); [d]Val5 spin system are 8.113(HN), 4.126(Hα),1.879(Hβ), 0.893(Hγ); [e]Gly7 spin system are 8.606(HN), 4.308, 3.946(Hα); [f]Gln12 spin system are 8.255(HN), 4.380(Hα), 1.977, 2.129(Hβ), 2.361(Hγ).

Table S4. The ^{1}H Chemical Shift Assignments for Peptide HPTLysAsp

Residue	HN	Hα	Hβ	Others
Ac-		2.029		
Arg1[a]	8.322	4.375	1.748, 1.834	Hγ: 1.612, 1.656; Hδ: 3.202; HNt: 7.216
Thr2	8.254	4.606	4.079	Hγ: 1.125
Val3[b]	8.486	4.231	2.026	Hγ: 0.890
Lys4	8.385	4.682	1.678	Hγ: 1.323; Hδ: 1.575; Hε: 2.929 NHt: 7.571
Val5[c]	8.632	4.522	1.999	Hγ: 0.932
^{D}Pro6		4.400	1.974, 2.340	Hγ: 2.043, 2.096; Hδ: 3.773, 3.792
Gly7[d]	8.464	3.840, 3.967		
Orn8[e]	8.056	4.527	1.791, 1.868	Hγ: 1.702; Hδ: 3.012; NHt: 7.621
Asp9[f]	8.503	*4.772*	2.541	
Ile10	8.593	4.260	1.897	Hγ: 1.214, 1.409, 0.888 (Me); Hδ: 0.819
Leu11	8.396	4.406	1.633	Hγ: 1.596; Hδ: 0.856, 0.906
Gln12[g]	8.439	4.302	1.947, 2.101	Hγ: 2.323, 2.344 ; HNt: 6.891, 7.462
NH_2	7.130, 7.617			

[a]The assignments for the minor Arg1 spin system are 8.345(HN), 4.341(Hα), 1.749, 1.835(Hβ), 1.618(Hγ) 3.206(Hδ); [b]Val3 spin system are 8.128(HN), 4.129(Hα), 1.882(Hβ), 0.897(Hγ); [c]Val5 spin system are 8.675(HN), 4.524(Hα), 1.997(Hβ), 0.926(Hγ); [d]Gly7 spin system are 8.614(HN), 3.946, 4.037(Hα); [e]Orn8 spin system are 7.887(HN), 4.006(Hα), 1.848(Hβ), 1.589(Hγ), 3.135, 3.350(Hδ); [f]Asp9 spin system are 8.792(HN), 4.719(Hα), 2.562, 2.708(Hβ); [g]Gln12 spin system are 8.313(HN), 4.293(Hα), 1.977, 2.123(Hβ), 2.346(Hγ).

Table S5. The [1]H Chemical Shift Assignments for Peptide HPTUDapAsp

Residue	HN	Hα	Hβ	Others
Ac-		2.043		
Arg1	8.348	4.339	1.757, 1.843	Hγ: 1.6281.669Hδ: 3.207 HNt: 7.207
Thr2	8.247	4.406	4.204	Hγ: 1.192
Val3	8.224	4.174	2.076	Hγ: 0.931
Dap4[a]	8.859	4.815	3.260, 3.431	
Val5	8.474	4.449	2.080	Hγ: 0.919, 0.979
Pro6		4.409	1.936, 2.314	Hγ: 1.995, 2.080; Hδ: 3.698, 3.887
Gly7[b]	8.493	3.970		
Orn8[a]	8.224	4.386	1.885	Hγ: 1.697, 1.761; Hδ: 3.003; NHt:
Asp9	8.441	4.616	2.591, 2.698	
Ile10[c]	8.082	4.137	1.887	Hγ: 1.191, 1.443, 0.900 (Me); Hδ: 0.861
Leu11	8.302	4.349	1.683	Hγ: 1.585, 1.619; Hδ: 0.868, 0.929
Gln12	8.266	4.287	1.977, 2.215	Hγ: 2.354, 2.375; HNt: 6.852, 7.543
NH_2	7.103, 7.526			

[a]Signal for the terminal HN not observed. [b]The assignments for the minor Gly7 spin system are 8.620(HN), 3.901, 4.036(Hα), Ile10 spin system are 8.144(HN), 4.139(Hα), 1.883, 1.200, 1.442(Hβ), 0.898(Hγ).

Table S6. The [1]H Chemical Shift Assignments for Peptide HPTUDabAsp

Residue	HN	Hα	Hβ	Others
Ac-		2.040		
Arg1	8.337	4.341	1.751, 1.839	Hγ: 1.623, 1.666; Hδ: 3.204; HNt: 7.208
Thr2	8.243	4.379	4.177	Hγ: 1.185
Val3	8.197	4.118	2.407	Hγ: 0.914
Dab4[a]	8.637	4.512	3.015, 3.054	Hγ: 2.048, 2.116
Val5[b]	8.419	4.420	2.079	Hγ: 0.934, 0.980
Pro6		4.402	1.939, 2.314	Hγ: 1.996, 2.078; Hδ: 3.703,3.886
Gly7[c]	8.468	3.972		
Orn8	8.241	4.379	1.887	Hγ: 1.700, 1.762 ; Hδ: 3.003
Asp9	8.437	4.596	2.584, 2.692	
Ile10	8.079	4.132	1.886	Hγ: 1.185, 1.442, 0.901 (Me); Hδ: 0.859
Leu11	8.297	4.345	1.685	Hγ: 1.583, 1.620; Hδ: 0.867, 0.930
Gln12	8.265	4.284	1.976, 2.125	Hγ: 2.352, 2.376; HNt: 6.877, 7.428
NH_2	7.103, 7.524			

[a]Signal for the terminal HN not observed. [b]The assignments for the minor Val5 spin system are 8.132(HN), 4.241(Hα), 1.996(Hβ), 0.897(Hγ); [c]Gly7 spin system are 8.604(HN), 3.895, 4.048(Hα).

Table S7. The ^{1}H Chemical Shift Assignments for Peptide HPTUOrnAsp

Residue	HN	Hα	Hβ	Others
Ac-		2.041		
Arg1[b]	8.332	4.344	1.749, 1.835	Hγ: 1.622, 1.660; Hδ: 3.205; HNt: 7.210
Thr2[c]	8.249	4.374	4.172	Hγ: 1.184
Val3[d]	8.185	4.115	2.041	Hγ: 0.913
Orn4	8.493	4.400	1.741,1.813	Hγ: 1.657; Hδ: 3.004
Val5[e]	8.373	4.420	2.077	Hγ: 0.939, 0.976
^{D}Pro6		4.401	1.941,2.313	Hγ: 1.991, 2.077; Hδ: 3.708, 3.883
Gly7	8.451	3.948, 3.992		
Orn8	8.249	4.373	1.883	Hγ: 1.710, 1.758; Hδ: 3.003; NHt: 7.613
Asp9	8.449	4.598	2.589, 2.700	
Ile10	8.077	4.135	1.889	Hγ: 1.183, 1.442, 0.904 (Me); Hδ: 0.862
Leu11	8.298	4.348	1.687,	Hγ: 1.579; Hδ: 0.867, 0.930
Gln12[f]	8.262	4.285	1.979, 2.128	Hγ: 2.353, 2.378; HNt: 6.852, 7.545
NH_2	7.103, 7.524			

[a]Signal for the terminal HN not observed. [b]The assignments for the minor Gly7 spin system are 8.597(HN), 3.893,4.044(Hα)

Table S8. The ^{1}H Chemical Shift Assignments for Peptide HPTULysAsp

Residue	HN	Hα	Hβ	Others
Ac-		2.306		
Arg1	8.327	4.344	1.748, 1.832	Hγ: 1.619, 1.660; Hδ: 3.203; HNt: 7.209
Thr2	8.257	4.367	4.165	Hγ: 1.179
Val3	8.184	4.115	2.307	Hγ: 0.911
Lys4	8.423	4.351	1.719, 1.766	Hγ: 1.343, 1.418; Hδ: 1.667
Val5[a]	8.339	4.419	2.066	Hγ: 0.934, .0968
Pro6		4.389	1.943, 2.308	Hγ: 1.977, 2.073; Hδ: 3.708, 3.876
Gly7[b]	8.457	3.972		
Orn8[a]	8.243	4.390	1.883	Hγ: 1.706, 1.766; Hδ: 3.005
Asp9	8.449	4.596	2.583, 2.697	
Ile10[c]	8.072	4.135	1.886	Hγ: 1.178, 1.443, 0.904 (Me); Hδ: 0.860
Leu11	8.301	4.345	1.684	Hγ: 1.585, 1.619 Hδ: 0.865, 0.928
Gln12	8.263	4.284	1.977, 2,.126	Hγ: 2.354, 2.375; HNt: 6.852, 7.544
NH_2	7.104, 7.525			

[a]Signal for the terminal HN not observed. [b]The assignments for the minor Val5 spin system are 7.983(HN), 4.257(Hα), 1.980(Hβ), 0.882(Hγ); [c]Gly7 spin system are 8.580(HN), 3.883, 4.025(Hα); [e]Ile11 spin system are 8.129(HN), 4.137(Hα), 1.883 (Hβ), 1.139, 1.443, (Hγ); 0.897(Hδ)

Table S9. The ¹H Chemical Shift Assignments for Peptide HPTFDapAsp

Residue	HN	Hα	Hβ	Others
Ac-		2.078		
Cys1	8.455	5.231	2.646, 3.169	
Arg2	8.764	4.659	1.826	Hγ: 1.607, 1.652; Hδ: 3.182; HNt: 7.195
Thr3	8.647	4.911	3.903	Hγ: 1.026
Val4	9.202	4.469	2.006	Hγ: 0.840
Dap5[a]	8.886	5.587	3.226, 3.343	
Val6	9.194	4.639	1.989	Hγ: 0.901
^{D}Pro7		4.347	1.965, 2.363	Hγ: 2.073; Hδ: 3.793, 3.845
Gly8	8.800	3.765, 4.035		
Orn9	7.821	4.826	1.774, 1.827	Hγ: 1.672; Hδ: 3.005; NHt: 7.602
Asp10	8.519	5.552	2.240, 2.513	
Ile11	9.088	4.482	1.824	Hγ: 1.074, 1.344, 0.855 (Me); Hδ: 0.798
Leu12	8.464	4.732	1.641	Hγ: 1.496; Hδ: 0.781, 0.811
Gln13	9.233	4.665	1.884, 2.078	Hγ: 2.296, 2.322; HNt: 6.832, 7.333
Cys14	9.009	5.093	3.007, 3.129	
NH_2	7.242, 7.597			

[a]Signal for the terminal HN not observed.

Table S10. The ¹H Chemical Shift Assignments for Peptide HPTFDabAsp

Residue	HN	Hα	Hβ	Others
Ac-		2.077		
Cys1	8.684	5.214	2.053, 2.095	
Arg2	8.761	4.670	1.833	Hγ: 1.533, 1.680; Hδ: 3.181; NHt: 7.132
Thr3	8.610	4.932	3.907	Hγ: 1.033
Val4	9.159	4.397	1.961	Hγ: 0.821, 0.856
Dab5[a]	8.468	5.235	3.166	Hγ: 2.642, 2.664
Val6	8.830	4.585	1.940	Hγ: 0.886, 0.901
^{D}Pro7		4.375	1.962, 2.342	Hγ: 2.038, 2.127; Hδ: 3.754, 3.867
Gly8	8.786	3.894, 3.969		
Orn9	7.864	4.688	1.793, 1.844	Hγ: 1.690 ; Hδ: 3.006; NHt: 7.623
Asp10	8.546	5.305	2.263, 2.486	
Ile11	9.191	4.463	1.868	Hγ: 1.143, 1.350, 0.858 (Me); Hδ: 0.797
Leu12	8.466	4.706	1.651	Hγ: 1.492; Hδ: 0.781, 0.816
Gln13	9.225	4.659	1.882, 2.074	Hγ: 2.219, 2.280; HNt: 6.845, 7.310
Cys14	9.005	5.080	3.001, 3.006	
NH_2	7.253, 7.613			

[a]Signal for the terminal HN not observed.

Table S11. The ^{1}H Chemical Shift Assignments for Peptide HPTFOrnAsp

Residue	HN	Hα	Hβ	Others
Ac-		2.078		
Cys1	8.449	5.216	3.161, 2.661	
Arg2[b]	8.759	4.660	1.819	Hγ: 1.683, 1.531; Hδ: 3.180; HNt: 7.123
Thr3	8.581	4.950	3.901	Hγ: 1.034
Val4	9.143	4.410	1.972	Hγ: 0.855, 0.815
Orn5[a]	8.464	5.097	1.738	Hγ: 1.597; Hδ: 2.885, 2.826
Val6	8.919	4.594	1.963, 1.923	Hγ: 0.910, 0.890
^{D}Pro7		4.366	2.346, 1.961	Hγ: 2.137, 2.039; Hδ: 3.771
Gly8	8.689	3.945, 3.871		
Orn9	7.897	4.667	1.829, 1.791	Hγ: 1.686 ; Hδ: 3.007; HNt: 7.164
Asp10	8.465	5.129	2.520, 2.270	
Ile11	9.260	4.438	1.891	Hγ: 1.364, 1.180, 0.789 (Me); Hδ: 0.855
Leu12	8.432	4.697	1.655	Hγ: 1.491; Hδ: 0.817, 0.78
Gln13[c]	9.219	4.666	2.074, 1.890	Hγ: 2.283, 2.217; HNt: 7.289
Cys14	8.978	5.081	3.132, 3.002	
NH_2	7.604, 7.240			

[a]Signal for the terminal HN not observed.

Table S12. The ^{1}H Chemical Shift Assignments for Peptide HPTFLysAsp

Residue	HN	Hα	Hβ	Others
Ac-		2.078		
Cys1	8.464	5.212	2.665, 3.165	
Arg2[a]	8.766	4.668	1.683, 1.833	Hγ: 1.531; Hδ: 3.178; NHt: 7.129
Thr3	8.586	4.972	3.910	Hγ: 0.809, 0.854
Val4	9.118	4.415	1.978	Hγ: 0.814, 0.855
Lys5	8.385	5.012	1.671	Hγ: 1.508, 1.570; Hδ: 1.266, 1.329; Hε: 2.903
Val6	8.953	4.591	1.955	Hγ: 0.874, 0.903
^{D}Pro7		4.355	1.959, 2.365	Hγ: 2.045, 2.141; Hδ: 3.809, 3.868
Gly8	8.635			
Orn9	7.917	4.650	1.790, 1.839	Hγ: 1.682; Hδ: 3.007; NHt: 7.615
Asp10	8.450	5.011	2.277, 2.520	
Ile11	9.289	4.444	1.909	Hγ: 1.218, 1.367, 0.859 (Me); Hδ: 0.794
Leu12	8.419	4.692	1.659	Hγ: 1.489; Hδ: 0.783, 0.816
Gln13	9.226	4.657	1.892, 2.072	Hγ: 2.218, 2.283; HNt: 6.844, 7.300
Cys14[b]	8.991	5.075	3.000, 3.134	
NH_2	7.250, 7.614			

[a]The assignments for the minor Arg2 spin system are 8.841(HN), 4.681(Hα), 1.701, 1.835(Hβ), 1.525(Hγ), 3.168(Hδ); [b]Cys14 spin system are 8.948(HN), 5.054(Hα), 2.993, 3.143(Hβ).

Table S13. The [1]H Chemical Shift Assignments for Peptide HPTDapGlu

Residue	HN	Hα	Hβ	Others
Ac-		2.033		
Arg1	8.304	4.390	1.757, 1.847	Hγ: 1.619, 1.670; Hδ: 3.211; HNt: 7.203
Thr2	8.180	4.843	4.075	Hγ: 1.085
Val3	8,784	4.390	2.015	Hγ: 0.894
Dap4[a]	8.790	5.116	3.300, 3.393	
Val5	8.858	4.599	1.966	Hγ: 0.879, 0.899
[D]Pro6		4.371	1.979, 2.354	Hγ: 2.062; Hδ: 3.794, 3.818
Gly7[b]	8.710	3.842, 4.021		
Orn8	7.988	4.603	1.801, 1.833	Hγ: 1.698; Hδ: 3.008; NHt: 7.609
Glu9	8.490	4.420	1.828, 1.885	Hγ: 2.189, 2.239
Ile10	8.819	4.390	1.851	Hγ: 1.144, 1.371, 0.867 (Me); Hδ: 0.787
Leu11	8.487	4.419	1.599	Hγ: 1.599; Hδ: 0.846, 0.889
Gln12	8.541	4.307	1.908, 2.062	Hγ: 2.288, 2.311; HNt: 6.874, 7.364
NH_2	7.128, 7.666			

[a]Signal for the terminal HN not observed. [b]The assignments for the minor Gly7 spin system are 8.629(HN), 3.947, 4.044(Hα)

Table S14. The [1]H Chemical Shift Assignments for Peptide HPTDabGlu

Residue	HN	Hα	Hβ	Others
Ac-		2.033		
Arg1	8.311	4.383	1.754, 1.842	Hγ: 1.618, 1.662; Hδ: 3.206; HNt: 7.206
Thr2	8.230	4.649	4.106	Hγ: 1.133
Val3[b]	8.517	4.263	2.023	Hγ: 0.894
Dab4[a]	8.644	4.814	2.058, 2.105	Hγ: 2.972, 2.995
Val5[c]	8.497	4.527	1.989	Hγ: 0.918
[D]Pro6		4.416	1.984, 2.329	Hγ: 2.038, 2.086; Hδ: 3.810
Gly7[d]	8.549	3.921, 3.979		
Orn8	8.092	4.525	1.876, 1.805	Hγ: 1.715; Hδ: 3.016; NHt: 7.624
Glu9	8.599	4.557	1.916	Hγ: 2.169, 2.277
Ile10	8.596	4.293	1.853	Hγ: 1.160, 1.410, 0.869 (Me); Hδ: 0.812
Leu11	8.408	4.436	1.602	Hγ: 1.602; Hδ: 0.850, 0.899
Gln12[e]	8.492	4.311	1.936, 2.086	Hγ: 2.316, 2.336; HNt: 6.870, 7.431
NH_2	7.118, 7.636			

[a]Signal for the terminal HN not observed. [b]The assignments for the minor Val3 spin system are 8.267(HN), 4.127(Hα), 1.849(Hβ), 0.882(Hγ); [c]Val5 spin system are 8.366(HN), 4.116(Hα), 2.008(Hβ), 0.881, 0.898(Hγ); [d]Gly7 spin system are 8.614(HN), 3.957, 4.040(Hα); [e]Gln12 spin system are 8.340(HN), 4.298(Hα), 1.974, 2.115(Hβ), 2.367(Hγ)

Table S15. The ^{1}H Chemical Shift Assignments for Peptide HPTOrnGlu

Residue	HN	Hα	Hβ	Others
Ac-		2.032		
Arg1	8.305	4.386	1.752, 1.840,	Hγ: 1.616, 1.661; Hδ: 3.206; HNt: 7.205
Thr2	8.231	4.663	4.099	Hγ: 1.128
Val3[b]	8.530	4.262	2.013	Hγ: 0.889
Orn4[a]	8.477	4.704	1.758	Hγ: 1.588, 1.656; Hδ: 2.943
Val5[c]	8.590	4.526	1.984	Hγ: 0.917
^{D}Pro6		4.409	1.980, 2.329	Hγ: 2.038, 2.096; Hδ: 3.810
Gly7[d]	8.545	3.933		
Orn8	8.071	4.542	1.806, 1.874	Hγ: 1.711; Hδ: 3.016; NHt: 7.619
Glu9	8.565	4.558	1.910	Hγ: 2.159, 2.257
Ile10	8.651	4.305	1.860	Hγ: 1.163, 1.400, 0.869 (Me); Hδ: 0.810
Leu11	8.403	4.443	1.599	Hγ: 1.599; Hδ: 0.849, 0.898
Gln12[e]	8.504	4.315	1.934, 2.085	Hγ: 2.310, 2.335; HNt: 6.873, 7.426
NH_2	7.118, 7.643			

[a]Signal for the terminal HN not observed. [b]The assignments for the minor Val3 spin system are 8.268(HN), 4.122(Hα), 1.845(Hβ), 0.882(Hγ); [c]Val5 spin system are 8.321(HN), 4.099(Hα), 2.013(Hβ), 0.808, 0.903(Hγ); [d]Gly7 spin system are 8.610(HN), 3.955, 4.033(Hα); [e]Gln12 spin system are 8.338(HN), 4.346(Hα), 1.977, 2.114(Hβ), 2.364(Hγ)

Table S16. The ^{1}H Chemical Shift Assignments for Peptide HPTLysGlu

Residue	HN	Hα	Hβ	Others
Ac-		2.032		
Arg1	8.303	4.382	1.748, 1.836	Hγ: 1.612, 1.656 ; Hδ: 3.204; HNt: 7.206
Thr2	8.237	4.633	4.094	Hγ: 1.128
Val3[a]	8.511	4.248	2.017	Hγ: 0.888
Lys4	8.404	4.687	1.674	Hγ: 1.230, 1.345; Hδ: 1.599; Hε: 2.921; HNt: 7.553
Val5[b]	8.570	4.520	1.990	Hγ: 0.923
^{D}Pro6		4.398	1.972, 2.335	Hγ: 2.042, 2.101; Hδ: 3.822
Gly7[c]	8.496	3.867, 3.967		
Orn8	8.041	4.543	1.797, 1.878	Hγ: 1.715; Hδ: 3.017; NHt: 7.616
Glu9	8.616	4.511	1.903	Hγ: 2.138, 2.241
Ile10	8.648	4.296	1.861	Hγ: 1.173, 1.409, 0.873 (Me); Hδ: 0.808
Leu11	8.392	4.438	1.598	Hγ: 1.598; Hδ: 0.849, 0.897
Gln12[d]	8.496	4.310	1.934, 2.086	Hγ: 2.315, 2.335; HNt: 6.872, 7.438
NH_2	7.114, 7.631			

[a]The assignments for the minor Val3 spin system are 8.177(HN), 4.107(Hα), 2.041(Hβ), 0.907(Hγ); [b]Val5 spin system are 8.268(HN), 4.122(Hα), 1.848(Hβ), 0.880(Hγ); [c]Gly7 spin system are 8.606(HN), 3.955, 4.038(Hα); [d]Gln12 spin system are 8.344(HN), 4.293(Hα), 1.973, 2.113(Hβ), 2.365(Hγ)

Table S17. The ^{1}H Chemical Shift Assignments for Peptide HPTUDapGlu

Residue	HN	Hα	Hβ	Others
Ac-		2.044		
Arg1	8.347	4.337	1.753, 1.842	Hγ: 1.625, 1.668; Hδ: 3.205; HNt: 7.208
Thr2	8.246	4.409	4.204	Hγ: 1.191
Val3	8.225	4.173	2.076	Hγ: 0.931
Dap4[a]	8.856	4.807	3.257, 3.430	
Val5	8.422	4.453	2.098	Hγ: 0.914, 0.977
^{D}Pro6		4.416	1.939, 2.320	Hγ: 1.998, 2.076; Hδ: 3.693, 3.881
Gly7[b]	8.253	3.962, 4.000		
Orn8[a]	8.246	4.379	1.892	1.706, 1.756; Hδ: 3.009
Glu9[c]	8.596	4.291	1.931, 2.020	Hγ: 2.246, 2.295
Ile10	8.234	4.136	1.856	Hγ: 1.188, 1.477, 0.891 (Me); Hδ: 0.859
Leu11	8.321	4.379	1.654	Hγ: 1.596; Hδ: 0.861, 0.924
Gln12	8.347	4.292	1.971, 2.112	Hγ: 2.352, 2.376; HNt: 6.855, 7.532
NH_2	7.100, 7.566			

[a]Signal for the terminal HN not observed. [b]The assignments for the minor Gly7 spin system are 8.655(HN), 3.915, 4.051(Hα); [c]Glu9 spin system are 8.647(HN), 4.279 (Hα), 1.932. 2.014(Hβ), 2.275(Hγ).

Table S18. The ^{1}H Chemical Shift Assignments for Peptide HPTUDabGlu

Residue	HN	Hα	Hβ	Others
Ac-		2.043		
Arg1	8.336	4.341	1.750, 1.838	Hγ: 1.623, 1.665; Hδ: 3.206; HNt: 7.195
Thr2	8.243	4.378	4.179	Hγ: 1.186
Val3	8.194	4.120	2.047	Hγ: 0.923
Dab4[a]	8.634	4.511	2.047, 2.114	Hγ: 3.013, 3.054
Val5[b]	8.384	4.425	2.081	Hγ: 0.934, 0982
^{D}Pro6		4.410	1.942, 2.321	Hγ: 1.998, 2.078; Hδ: 3.703, 3.882
Gly7[c]	8.501	3.959, 4.011		
Orn8[a]	8.260	4.378	1.765, 1.896	Hγ: 1.709; Hδ: 3.013
Glu9	8.605	4.283	1.933, 2.020	Hγ: 2.238, 2.287
Ile10	8.228	4.135	1.858	Hγ: 1.190, 1.481, 0.889 (Me); Hδ: 0.855
Leu11	8.320	4.377	1.654	Hγ: 1.593; Hδ: 0.864, 0.925
Gln12	8.347	4.294	1.977, 2.115	Hγ: 2.356, 2.379; HNt: 6.857, 7.534
NH_2	7.101, 7.567			

[a]Signal for the terminal HN not observed. [b]The assignments for the minor Val5 spin system are 8.141(HN), 4.250(Hα), 1.996(Hβ), 0.900(Hγ); [c]Gly7 spin system are 8.637(HN), 3.908, 4.409(Hα).

Table S19. The ^{1}H Chemical Shift Assignments for Peptide HPTUOrnGlu

Residue	HN	Hα	Hβ	Others
Ac-		2.043		
Arg1	8.330	4.344	1.838, 1.750	Hγ: 1.623, 1.663; Hδ: 3.205; HNt: 7.206
Thr2	8.249	4.377	4.175	Hγ: 1.183
Val3[b]	8.184	4.116	2.044	Hγ: 0.919
Orn4[a]	8.488	4.398	1.740, 1.816	Hγ: 1.656; Hδ: 3.008
Val5	8.330	4.424	2.076	Hγ: 0.938, 0.978
Pro6		4.409	1.943, 2.317	Hγ: 1.998, 2.072; Hδ: 3.706, 3.879
Gly7[c]	8.476	3.953, 4.013		
Orn8	8.262	4.373	1.894	Hγ: 1.702, 1.768; Hδ: 3.015; NHt: 7.624
Glu9	8.589	4.293	1.943, 2.027	Hγ: 2.268, 2.317
Ile10	8.233	4.136	1.859	Hγ: 1.482, 1.183, 0.919 (Me); Hδ: 0.854
Leu11	8.321	4.381	1.659	Hγ: 1.584; Hδ: 0.865, 0.927
Gln12	8.341	4.296	1.976, 2.116	Hγ: 2.356, 2.379; HNt: 6.857, 7.534
NH_2	7.103, 7.569			

[a]Signal for the terminal HN not observed. [b]The assignments for the minor Val3 spin system are 8.069(HN), 4.258(Hα), 1.994(Hβ), 0.903(Hγ); [c]Gly spin system are 8.621(HN), 3.897, 4.046(Hα)

Table S20. The ^{1}H Chemical Shift Assignments for Peptide HPTULysGlu

Residue	HN	Hα	Hβ	Others
Ac-				
Arg1	8.324	4.343	1.749, 1.833	Hγ: 1.620, 1.659; Hδ: 3.202; HNt: 7.206
Thr2	8.258	4.370	4.166	Hγ: 1.180
Val3[a]	8.181	4.115	2.040	Hγ: 0.914
Lys4	8.418	4.350	1.714	Hγ: 1.345, 1.416; Hδ: 1.666; Hε: 2.979; HNt: 7.536
Val5	8.290	4.419	2.068	Hγ: 0.934, 0.970
Pro6		4.396	1.945, 2.311	Hγ: 1.988, 2.072; Hδ: 3.707, 3.872
Gly7[b]	8.486	3.960, 4.010		
Orn8	8.261	4.370	1.895	Hγ: 1.711, 1.769; Hδ: 3.015; NHt: 7.618
Glu9	8.604	4.287	2.256, 2.277	Hγ: 1.935, 2.022
Ile10	8.228	4.135	1.857	Hγ: 1.477, 1.181, 0.891 (Me); Hδ: 0.861
Leu11	8.320	4.379	1.573	Hγ: 1.592; Hδ: 0.863, 0.927
Gln12	8.344	4.294	1.970, 2.116	Hγ: 2.355, 2.379; HNt: 6.857, 7.533
NH_2	7.102, 7.568			

[a]The assignments for the minor Val3 spin system are 7.985(HN), 4.261(Hα), 1.990(Hβ), 0.903(Hγ); [b]Gly7 spin system are 8.607(HN), 3.902, 4.039(Hα),

Table S21. The ^{1}H Chemical Shift Assignments for Peptide HPTFDapGlu.

Residue	HN	Hα	Hβ	Others
Ac-		2.083		
Cys1	8.446	5.219	2.678, 3.168	
Arg2	8.752	4.688	1.830, 1.860	Hγ: 1.535, 1.686; Hδ: 3.185; HNt: 7.131
Thr3	8.558	5.017	3.948	Hγ: 1.077
Val4	9.223	4.475	2.000	Hγ: 0.846
Dap5[a]	8.875	5.287	3.309, 3.371	
Val6	9.035	4.638	1.941	Hγ: 0.866, 0.890
^{D}Pro7		4.344	1.974, 2.367	Hγ: 2.050, 2.093; Hδ: 3.771, 3.848
Gly8	8.831	3.804, 4.041		
Orn9	7.908	4.690	1.811	Hγ: 1.688; Hδ: 3.006; NHt: 7.610
Glu10	8.423	5.038	1.745, 1.879	Hγ: 2.142, 2.191
Ile11	9.078	4.470	1.832	Hγ: 1.111, 1.337, 0.866 (Me); Hδ: 0.777
Leu12	8.508	4.655	1.685	Hγ: 1.496; Hδ: 0.787, 0.828
Gln13	9.225	4.664	1.887, 2.067	Hγ: 2.220, 2.278; HNt: 6.825, 7.303
Cys14	8.974	5.083	3.006, 3.134	
NH_2	7.242, 7.606			

[a]Signal for the terminal HN not observed.

Table S22. The ^{1}H Chemical Shift Assignments for Peptide HPTFDabGlu

Residue	HN	Hα	Hβ	Others
Ac-		2.083		
Cys1	8.450	5.215	2.657, 3.168	
Arg2	8.758	4.683	1.825, 1.852	Hγ: 1.544, 1.685; Hδ: 3.186; HNt: 7.195
Thr3	8.583	4.950	3.948	Hγ: 1.069
Val4	9.120	4.445	1.985	Hγ: 0.815, 0.854
Dab5[a]	8.710	5.130	2.074, 2.101	Hγ: 2.931, 2.966
Val6	8.599	4.586	1.913	Hγ: 0.870, 0.899
^{D}Pro7		4.380	1.969, 2.347	Hγ: 2.043, 2.133; Hδ: 3.744, 3.867
Gly8	8.775	3.906, 3.994		
Orn9	7.938	4.703	1.823, 1.869	Hγ: 1.705 ; Hδ: 3.011; NHt: 7.624
Glu10	8.567	4.910	1.998, 2.228	Hγ: 1.783, 1.878
Ile11	9.052	4.492	1.834	Hγ: 1.094, 1.329, 0.851 (Me); Hδ: 0.789
Leu12	8.450	4.715	1.658	Hγ: 1.499 Hδ: 0.785, 0.820
Gln13	9.207	4.660	1.879, 2.078	Hγ: 2.214, 2.272; HNt: 6.826, 7.320
Cys14	8.989	5.083	3.006, 3.134	
NH_2	7.241, 7.605			

[a]Signal for the terminal HN not observed.

Table S23. The ^{1}H Chemical Shift Assignments for Peptide HPTFOrnGlu

Residue	HN	Hα	Hβ	Others
Ac-		2.077		
Cys1	8.468	5.213	2.649, 3.167	
Arg2	8.770	4.679	1.824	Hγ: 1.687; Hδ: 3.182; HNt: 7.134
Thr3	8.599	4.946	3.939	Hγ: 1.060
Val4	9.137	4.423	1.971	Hγ: 0.814, 0.851
Orn5[a]	8.555	4.954	1.753, 1.807	Hγ: 1.580, 1.653; Hδ: 2.933
Val6	8.767	4.571	1.924	Hγ: 0.870, 0.895
^{D}Pro7		4.376	1.969, 2.335	Hγ: 2.036, 2.134; Hδ: 3.724, 3.864
Gly8	8.754	3.934		
Orn9	7.953	4.688	1.817,1.862	Hγ: 1.698; Hδ: 3.008; NHt: 7.603
Glu10	8.515	4.891	1.778, 1.877	Hγ: 2.000, 2.208
Ile11	9.103	4.496	1.844	Hγ: 1.109, 1.324, 0.848 (Me); Hδ: 0.789
Leu12	8.447	4.725	1.653	Hγ: 1.492; Hδ: 0.780, 0.814
Gln13	9.220	4.659	1.872, 2.072	Hγ: 2.212, 2.269; HNt: 6.840, 7.329
Cys14	9.008	3.001, 3.133		
NH_2	7.254, 7.615			

[a]Signal for the terminal HN not observed.

Table S24. The ^{1}H Chemical Shift Assignments for Peptide HPTFLysGlu

Residue	HN	Hα	Hβ	Others
Ac-		2.080		
Cys1	8.449	5.211	2.656, 3.170	
Arg2	8.758	4.682	1.837	Hγ: 1.541, 1.683; Hδ: 3.182; NHt: 7.128
Thr3	8.575	4.953	3.941	Hγ: 1.061
Val4	9.083	4.420	1.975	Hγ: 0.814, 0.853
Lys5[a]	8.462	4.947	1.670, 1.708	Hγ: 1.225, 1.313; Hδ: 1.606 ; Hε: 2.906
Val6	8.758	4.575	1.937	Hγ: 0.878, 0.903
^{D}Pro7		4.368	1.959, 2.350	Hγ: 2.041, 2.139; Hδ: 3.767, 3.857
Gly8	8.672	3.855, 3.970		
Orn9	7.938	4.674	1.810, 1.868	Hγ: 1.691; Hδ: 3.011; NHt: 7.615
Glu10	8.555	4.817	1.781, 1.871	Hγ: 1.982, 2.191
Ile11	8.398	4.492	1.851	Hγ: 1.120, 1.341, 0.854 (Me); Hδ: 0.789
Leu12	8.398	4.724	1.650	Hγ: 1.495; Hδ: 0.782, 0.817
Gln13	9.203	4.662	1.875, 2.075	Hγ: 2.209, 2.272; HNt: 6.829, 7.321
Cys14	8.986	5.076	3.004, 3.131	
NH_2	7.238, 7.603			

Table S25. The ^{1}H Chemical Shift Assignments for Peptide HPTDapAad

Residue	HN	Hα	Hβ	Others
Ac-		2.038		
Arg1	8.328	4.376	1.757, 1.844	Hγ: 1.665; Hδ: 3.207; HNt: 7.207
Thr2	8.246	4.606	4.119	Hγ: 1.155
Val3[a]	8.464	4.290	2.077	Hγ: 0.912
Dap4[b]	8.872	5.001	3.269, 3.418	
Val5[c]	8.543	4.555	2.025	Hγ: 0.912
^{D}Pro6		4.423	1.597, 2.330	Hγ: 1.986, 2.049; Hδ: 3.790, 3.836
Gly7[d]	8.508	3.922, 3.954		
Orn8[e]	8.120	4.488	1.799, 1.874	Hγ: 1.710; Hδ: 3.017; NHt:
Aad9	8.475	4.512	1.612, 1.697	Hγ: 2.191; Hδ: 1.490
Ile10	8.451	4.258	1.835	Hγ: 1.154, 1.418, 0.869(Me); Hδ: 0.820
Leu11	8.397	4.428	1.596	Hγ: 1.831; Hδ: 0.850
Gln12[f]	8.470	4.302	1.944, 2.083	Hγ: 2.337; HNt: 6.867
NH_2	7.118, 7.635			

[a]The assignments for the minor Val3 spin system are 8.128(HN), 4.178 (Hα), 2.080(Hβ), 0.931(Hγ); [b]Signal for the terminal HN not observed; [c]Val5 spin system are 8.279(HN), 4.127(Hα), 1.833(Hβ), 0.879(Hγ); [d]Gly7 spin system are 8.613(HN), 3.926, 4.029(Hα); [e]Orn8 spin system are 8.361(HN), 4.193(Hα),1.753, 1.855(Hβ), 1.677(Hγ) ; [f]Gln12 spin system are 8.357(HN),4.344(Hα), 1.975, 2.111(Hβ), 2.365(Hγ).

Table S26. The ^{1}H Chemical Shift Assignments for Peptide HPTDabAad

Residue	HN	Hα	Hβ	Others
Ac-		2.035		
Arg1[a]	8.309	4.394	1.754, 1.842	Hγ: 1.614, 1.659; Hδ: 3.207; HNt: 7.206
Thr2	8.248	4.681	4.077	Hγ: 1.133
Val3[b]	8.547	4.287	2.013	Hγ: 0.890
Dab4[c]	8.667	4.887	2.964	Hγ: 2.021, 2.108
Val5[d]	8.527	4.544	2.068	Hγ: 0.918
^{D}Pro6		4.409	1.980, 2.330	Hγ: 2.041, 2.098; Hδ: 3.206, 3.815
Gly7[e]	8.569	3.943		
Orn8[f]	8.060	4.558	1.806, 1.870	Hγ: 1.711; Hδ: 3.015; HNt: 7.622
Aad9	8.529	4.597	1.633, 1.696	Hγ: 2.174; Hδ: 1.450
Ile10	8.606	4.313	1.841	Hγ: 1.139, 1.399, 0.865(Me); Hδ: 0.807
Leu11	8.409	4.453	1.593	Hγ: 1.706; Hδ: 0.845, 0.894
Gln12[g]	8.529	4.306	1.940, 2.085	Hγ: 2.321; HNt: 6.872, 7.428
NH_2	7.126, 7.656			

[a]The assignments for the minor Arg1 spin system are 8.337(HN), 4.358(Hα),1.757, 1.843(Hβ), 1.579, 1.653(Hγ), 3.212(Hδ).[b]Val3 spin system are 8.275(HN), 4.128 (Hα), 1.838(Hβ), 0.877(Hγ); [c]Signal for the terminal HN not observed; [d]Val5 spin system are 8.194(HN), 4.124(Hα), 2.054(Hβ), 0.921(Hγ); [e]Gly7 spin system are 8.606(HN), 4.024(Hα); [f]Orn8 spin system are 8.364(HN), 4.360(Hα), 1.880(Hβ), 1.755(Hγ); [g]Gln12 spin system are 8.359(HN), 4.295(Hα),1.975 (Hβ), 2.369(Hγ).

Table S27. The ^{1}H Chemical Shift Assignments for Peptide HPTOrnAad

Residue	HN	Hα	Hβ	Others
Ac-		2.029		
Arg1	8.302	4.401	1.751, 1.840	Hγ: 1.610, 1.659; Hδ: 3.205; HNt: 7.201
Thr2	8.243	4.728	4.057	Hγ: 1.118
Val3[a]	8.587	4.295	2.005	Hγ: 0.880
Orn4[b]	8.493	4.766	1.719, 1.770	Hγ: 1.570, 1.646; Hδ: 2.933
Val5	8.663	4.544	1.975	Hγ: 0.913
^{D}Pro6		4.400	1.976, 2.327	Hγ: 2.039, 2.105; Hδ: 3.786, 3.831
Gly7	8.583	3.926		
Orn8	8.032	4.582	1.805, 1.863	Hγ: 1.703; Hδ: 3.012; HNt: 7.618
Aad9	8.507	4.639	1.611, 1.684	Hγ: 2.165; Hδ: 1.432
Ile10	8.702	4.336	1.848	Hγ: 1.145, 1.390, 0.863(Me); Hδ: 0.799
Leu11	8.409	4.461	1.589	Hγ: 1.711; Hδ: 0.845, 0.886
Gln12[c]	8.547	4.310	1.921, 2.075	Hγ: 2.312; HNt: 6.876, 7.406
NH_2	7.128, 7.665			

[a]The assignments for the minor Val3 spin system are 8.189(HN), 4.114(Hα), 2.047(Hβ), 0.914(Hγ); [b]Signal for the terminal HN not observed; [c]The assignments for the minor Gln12 spin system are 8.358(HN), 4.295(Hα), 1.754, 1.974(Hβ), 2.364(Hγ).

Table S28. The ^{1}H Chemical Shift Assignments for Peptide HPTLysAad

Residue	HN	Hα	Hβ	Others
Ac-		2.029		
Arg1	8.350	4.388	1.748, 1.831	Hγ: 1.608, 1.655; Hδ: 3.202; HNt: 7.226
Thr2	8.306	4.698	4.063	Hγ: 1.122
Val3[a]	8.599	4.273	2.008	Hγ: 0.879
Lys4[b]	8.461	4.770	1.653	Hγ: 1.200, 1.303; Hε: 2.894
Val5[c]	8.678	4.540	1.978	Hγ: 0.922
^{D}Pro6		4.386	1.970, 2.346	Hγ: 2.042, 2.111; Hδ: 3.834
Gly7[d]	8.580	3.838, 3.969		
Orn8	8.020	4.591	1.795, 1.867	Hγ: 1.694; Hδ: 3.010; HNt: 7.158
Aad9	8.610	4.556	1.617, 1.690	Hγ: 2.152, 2.186; Hδ: 1.444
Ile10[e]	8.747	4.317	1.857	Hγ: 1.168, 1.394, 0.864(Me); Hδ: 0.796
Leu11	8.452	4.451	1.589	Hδ: 0.842, 0.891
Gln12[f]	8.581	4.303	1.932, 2.080	Hγ: 2.318; HNt: 6.920
NH_2	7.703			

[a]The assignments for the minor Val3 spin system are 8.208(HN), 4.038(Hα), 2.034(Hβ), 0.955(Hγ); [b]Signal for the terminal HN not observed.; [c]The assignments for the minor Val5 spin system are 8.243(HN), 4.104(Hα), 2.034(Hβ), 0.913(Hγ); [d]The assignments for the minor Gly7 spin system are 8.644(HN), 3.929, 4.017(Hα); [e]The assignments for the minor Ile10 spin system are 8.374(HN), 4.075(Hα), 1.744(Hβ), 0.798, 0.902(Hγ); [f]The assignments for the minor Gln12 spin system are 8.422(HN), 4.302(Hα), 1.971, 2.113(Hβ), 2.369(Hγ).

Table S29. The ^{1}H Chemical Shift Assignments for Peptide HPTUDapAad

Residue	HN	Hα	Hβ	Others
Ac-		2.039		
Arg1	8.365	4.336	1.756, 1.843	Hγ: 1.628, 1.670; Hδ: 3.207; HNt: 7.216
Thr2	8.264	4.405	4.204	Hγ: 1.193
Val3	8.237	4.171	2.075	Hγ: 0.932
Dap4[a]	8.873	*4.805*	3.256, 3.429	
Val5	8.453	4.452	2.098	Hγ: 0.915, 0.982
Pro6		4.412	1.937, 2.318	Hγ: 2.001, 2.078; Hδ: 3.697, 3.883
Gly7[b]	8.479	3.927, 3.997		
Orn8[a]	8.234	4.391	1.878	Hγ: 1.707, 1.751 ; Hδ: 3.011;
Aad9[c]	8.406	4.288	1.704, 1.751	Hγ: 1.535, 1.619; Hδ: 2.205, 2.245
Ile10[d]	8.269	4.122	1.840	Hγ: 1.185, 1.489, 0.882 (Me); Hδ: 0.851
Leu11	8.356	4.376	1.648	Hγ: 1.574; Hδ: 0.860, 0.923
Gln12	8.379	4.288	1.975, 2.112	Hγ: 2.352, 2.381; HNt: 6.875, 7.555
NH_2	7.114, 7.595			

[a]Signal for the terminal HN not observed; [b]The assignments for the minor Gly7 spin system are 8.638(HN), 3.893, 4.022(Hα); [c]Aad9 spin system are 8.450(HN), 4.281(Hα), 1.721(Hβ), 1.536, 1.624(Hγ); [d]Ile10 spin system are 8.313(HN), 4.131(Hα), 1.834(Hβ), 1.190, 1.484(Hγ); 0.879(Me(Hγ)); 0.879(Hδ).

Table S30. The ^{1}H Chemical Shift Assignments for Peptide HPTUDabAad

Residue	HN	Hα	Hβ	Others
Ac-		2.040		
Arg1	8.353	4.338	1.748, 1.835	Hγ: 1.619, 1.665; Hδ: 3.203; HNt: 7.215
Thr2	8.258	4.377	4.178	Hγ: 1.184
Val3	8.213	4.116	2.044	Hγ: 0.919
Dab4[a,b]	8.657	4.509	3.009, 3.054	Hγ: 2.048, 2.116;
Val5[c]	8.403	4.426	2.086	Hγ: 0.933, 0.980
Pro6		4.406	1.964, 2.314	Hγ: 2.072; Hδ: 3.702, 3.879
Gly7[d]	8.457	3.923, 3.999		
Orn8	8.244	4.387	1.875	Hγ: 1.707, 1.761 ; Hδ: 3.011; NHt: 7.627
Aad9[e]	8.407	4.289	1.704, 1.751	Hγ: 1.541, 1.618; Hδ: 2.216, 2.255
Ile10[f]	8.274	4.123	1.838	Hγ: 1.486, 1.184, 0.881 (Me); Hδ: 0.848
Leu11	8.354	4.375	1.648	Hγ: 1.571; Hδ: 0.857, 0.924
Gln12	8.376	4.288	1.976, 2.109	Hγ: 2.353, 2.380; HNt: 6.875, 7.554
NH_2	7.114, 7.595			

[a]Signal for the terminal HN not observed. [b]The assignments for the minor Dab4 spin system are 8.602(HN), 4.483(Hα), 3.040(Hβ), 2.039, 2.130(Hγ); [c]Val5 spin system are 8.158(HN), 4.246(Hα), 1.997(Hβ), 0.901(Hγ); [d]Gly7 spin system are 8.662(HN), 3.882, 4.034(Hα); [e]Aad9 spin system are 8.450(HN), 4.280(Hα), 1.718(Hβ), 1.536, 1.620(Hγ), 2.232(Hδ); [f]Ile10 spin system are 8.311(HN), 4.129(Hα), 1.833(Hβ), 1.482, 1.182(Hγ), 0.879(Me(Hγ)).

Table S31. The ^{1}H Chemical Shift Assignments for Peptide HPTUOrnAad

Residue	HN	Hα	Hβ	Others
Ac-		2.039		
Arg1	8.347	4.341	1.750, 1.834	Hγ: 1.619, 1.663; Hδ: 3.204; HNt: 7.215
Thr2	8.266	4.375	4.171	Hγ: 1.183
Val3[b]	8.206	4.112	2.041	Hγ: 0.917
Orn4[a,c]	8.513	4.396	1.815	Hγ: 1.654, 1.739; Hδ: 3.007
Val5	8.360	4.425		Hγ: 0.937, 0.978
Pro6		4.403	1.939, 2.317	Hγ: 1.998, 2.072; Hδ: 3.701, 3.879
Gly7	8.447	3.922, 4.003		
Orn8	8.248	4.337	1.877	Hγ: 1.697, 1.757 ; Hδ: 3.011; NHt: 7.624
Aad9	8.414	4.289	1.706, 1.745	Hγ: 1.536, 1.624; Hδ: 2.214, 2.253
Ile10	8.278	4.122	1.840	Hγ: 1.493, 1.184, 0.881 (Me); Hδ: 0.853
Leu11	8.356	4.376	1.648	Hγ: 1.574; Hδ: 0.862, 0.924
Gln12	8.380	4.289	1.971, 2.113	Hγ: 2.352, 2.382; HNt: 6.874, 7.555
NH_2	7.115, 7.593			

[a]Signal for the terminal HN not observed; [b]The assignments for the minor Val3 spin system are 8.094(HN), 4.250(Hα), 1.994(Hβ), 0.900(Hγ); [c]Orn4 spin system are 8.464(HN), 4.370(Hα), 1.829(Hβ), 1.662, 1.737(Hγ), 3.009(Hδ).

Table S32. The ^{1}H Chemical Shift Assignments for Peptide HPTULysAad

Residue	HN	Hα	Hβ	Others
Ac-		2.042		
Arg1	8.327	4.343	1.748, 1.883	Hγ: 1.618, 1.659; Hδ: 3.204; HNt: 7.208
Thr2	8.257	4.369	4.165	Hγ: 0.923
Val3[a]	8.183	4.116	2.039	Hγ: 0.912
Lys4	8.424	4.349	1.759	Hγ: 1.343, 1.417; Hδ: 1.719; Hε: 2.976; HNt: 7.541
Val5	8.285	4.423	2.072	Hγ: 0.932, 0.967
Pro6		4.392	1.945, 2.310	Hγ: 1.985, 2.069; Hδ: 3.701, 3.879
Gly7	8.437	3.926, 3.994		
Orn8	8.228	4.391	1.880	Hγ: 1.710, 1.768 ; Hδ: 3.013; NHt: 7.617
Aad9	8.395	4.292	1.706, 1.752	Hγ: 1.535, 1.623; Hδ: 2.210, 2.247
Ile10	8.252	4.126	1.840	Hγ: 1.477, 1.180, 0.881 (Me); Hδ: 0.855
Leu11	8.334	4.375	1.649	Hγ: 1.574; Hδ: 0.863, 0.922
Gln12	8.357	4.292	1.974, 2.112	Hγ: 2.355, 2.375; HNt: 6.874, 7.555
NH_2	7.102, 7.582			

[a]The assignments for the minor Val3 spin system are 7.984(HN), 4.257(Hα), 1.994(Hβ), 0.905(Hγ).

Table S33. The [1]H Chemical Shift Assignments for Peptide HPTFDapAad

Residue	HN	Hα	Hβ	Others
Ac-		2.082		
Cys1	8.450	5.220	2.661, 3.168	
Arg2	8.765	4.690	1.840	Hγ: 1.547, 1.683; Hδ: 3.186; HNt: 7.316
Thr3	8.583	4.974	3.946	Hγ: 1.085
Val4	9.161	4.502	2.035	Hγ: 0.849, 0.874
Dap5[a]	8.911	5.299	3.310, 3.390	
Val6	8.780	4.632	1.936	Hγ: 0.870, 0.901
[D]Pro7		4.368	1.969, 2.367	Hγ: 2.044, 2.106; Hδ: 3.776, 3.850
Gly8	8.783	3.859, 3.999		
Orn9	7.946	4.673	1.845	Hγ: 1.701; Hδ: 3.007; NHt: 7.618
Aad10	8.513	4.960	1.583, 1.705	Hγ:1.400, 1.522; Hδ: 2.099
Ile11	8.899	4.491	1.809	Hγ: 1.096, 1.323, 0.845 (Me); Hδ: 0.785
Leu12	8.479	4.708	1.667	Hγ: 1.493; Hδ: 0.781, 0.820
Gln13	9.205	4.643	1.857, 2.074	Hγ: 2.210, 2.267; HNt: 6.822, 7.334
Cys14	8.991	5.085	3.006, 3.133	
NH_2	7.242, 7.607			

[a]Signal for the terminal HN not observed.

Table S34. The [1]H Chemical Shift Assignments for Peptide HPTFDabAad

Residue	HN	Hα	Hβ	Others
Ac-		2.082		
Cys1	8.449	5.223	2.654, 3.170	
Arg2	8.763	4.693	1.842	Hγ: 1.542, 1.688; Hδ: 3.185 NHt: 7.128
Thr3	8.594	4.976	3.945	Hγ: 1.080
Val4	9.103	4.460	1.988	Hγ: 0.814, 0.855
Dab5[a]	8.747	5.172	2.013, 2.122	Hγ: 2.910, 2.942
Val6	8.619	4.604	1.918	Hγ: 0.874, 0.903
[D]Pro7		4.378	1.968, 2.348	Hγ: 2.041, 2.134; Hδ: 3.754, 3.867
Gly8	8.768	3.905, 3.992		
Orn9	7.940	4.711	1.847	Hγ: 1.703 ; Hδ: 3.013; NHt: 7.623
Aad10	8.575	4.926	1.602, 1.694	Hγ: 1.373, 1.499; Hδ: 2.082, 2.102
Ile11	9.019	4.497	1.823	Hγ: 1.083, 1.328, 0.850 (Me); Hδ: 0.795
Leu12	8.448	4.713	1.661	Hγ: 1.488; Hδ: 0.782, 0.817
Gln13	9.215	4.663	1.877, 2.075	Hγ: 2.210, 2.269; HNt: 6.863, 7.544
Cys14	8.990	5.085	3.006, 3.131	
NH_2	7.240, 7.606			

[a]Signal for the terminal HN not observed.

Table S35. The ^{1}H Chemical Shift Assignments for Peptide HPTFOrnAad

Residue	HN	Hα	Hβ	Others
Ac-		2.078		
Cys	8.455	5.221	2.643, 3.169	
Arg1	8.760	4.687	1.841	Hγ: 1.539, 1.684; Hδ: 3.186; HNt: 7.125
Thr2	8.585	4.985	3.939	Hγ: 1.076
Val3	9.102	4.434	1.969	Hγ: 0.815
Orn4 [a]	8.555	4.951	1.711, 1.810	Hγ: 1.554, 1.622; Hδ: 2.916
Val5	8.827	4.577	1.936	Hγ: 0.885
^{D}Pro6		4.375	1.970, 2.335	Hγ: 2.035, 2.137; Hδ: 3.731, 3.864
Gly7	8.729	3.930		
Orn8	7.938	4.710	1.835	Hγ: 1.695; Hδ: 3.009; NHt: 7.614
Aad9	8.507	4.909	1.587, 1.666	Hγ: 2.081; Hδ: 1.358, 1.486
Ile10	9.084	4.493	1.842	Hγ: 1.108, 1.330, 0.852(Me); Hδ: 0.785
Leu11	8.434	4.710	1.486	Hγ: 1.657; Hδ: 0.781, 0.815
Gln12	9.219	4.660	2.214, 2.267	Hγ: 1.875, 2.074; HNt: 6.829, 7.316
Cys	8.995	5.086	3.001	
NH_2	7.242, 7.605			

[a]Signal for the terminal HN not observed.

Table S36. The ^{1}H Chemical Shift Assignments for Peptide HPTFLysAad

Residue	HN	Hα	Hβ	Others
Ac-		2.079		
Cys	8.445	5.221	2.655, 3.168	
Arg1	8.763	4.674	1.841	Hγ: 1.538, 1.687; Hδ: 3.182 NHt: 7.127
Thr2	8.577	4.991	3.941	Hγ: 1.079
Val3	9.067	4.430	1.975	Hγ: 0.811, 0.850
Lys4[a]	8.470	4.957	1.599, 1.720	Hγ: 1.193, 1.250; Hδ: 2.859, 2.899
Val5	8.805	4.580	1.937	Hγ: 0.880, 0.908
^{D}Pro6		4.364	1.959, 2.351	Hγ: 2.040, 2.141; Hδ: 3.776, 3.861
Gly7	8.666	3.842, 3.974		
Orn8	7.933	4.694	1.807, 1.858	Hγ: 1.686; Hδ: 3.008; NHt: 7.618
Aad9	8.545	4.809	1.598, 1.689	Hγ: 1.373, 1.487; Hδ: 2.073, 2.112
Ile10	9.139	4.479	1.850	Hγ: 1.128, 1.343, 0.851 (Me); Hδ: 0.787
Leu11	8.403	4.714	1.657	Hγ: 1.485; Hδ: 0.782, 0.817
Gln12	9.213	4.664	1.879, 2.074	Hγ: 2.210, 2.268; HNt: 6.826, 7.312
Cys	8.981	5.084	2.999, 3.132	
NH_2	7.238, 7.606			

[a]Signal for the terminal HN not observed.

Table S37. The $^{3}J_{HN\alpha}$ (Hz) Values of the HPTXaaAsp Peptides

	Xaa			
Residue	Dap	Dab	Orn	Lys
Arg1	10	10	10	8.8
Thr2	11	11	8.8	10
Val3	9.4	10	11	8.8
**Xaa4**	11	11	13	11
Val5	10	12	8.9	11
Gly7	10	9.2	10	10
Orn8	12	11	7.6	10
**Asp9**	9.2	11	10	11
Ile10	9.2	11	8.8	11
Leu11	12	11	10	8.8
Gln12	10	10	10	10

Table S38. The $^{3}J_{HN\alpha}$ (Hz) Values of the HPTXaaGlu Peptides

	Xaa			
Residue	Dap	Dab	Orn	Lys
Arg1	8.8	8.8	10	8.6
Thr2	11	10	11	11
Val3	9.9	9.8	10	8.4
**Xaa4**	11	10	10	10
Val5	10	9.4	10	10
Gly7	8.4	8.8	12	8.8
Orn8	10	10	10	10
**Glu9**	9.2	9.6	10	10
Ile10	9.0	11	11	11
Leu11	8.8	9.9	8.6	10
Gln12	8.6	9.0	7.7	8.6

Table S39. The $^{3}J_{HN\alpha}$ (Hz) Values of the HPTXaaAad Peptides.

	Xaa			
Residue	Dap	Dab	Orn	Lys
Arg1	11	10	9.9	8.8
Thr2	11	11	10	11
Val3	11	10	8.6	10
**Xaa4**	12	10	11	8.1
Val5	12	11	11	11
Gly7	11	11	14	9.0
Orn8	11	11	10	11
**Aad9**	11	11	11	9.9
Ile10	10	10	11	9.4
Leu11	10	10	9.8	9.9
Gln12	9.0	10	8.4	9.8

Table S40. The $^{3}J_{HN\alpha}$ (Hz) Values of the HPTUXaaAsp Peptides.

	Xaa			
Residue	Dap	Dab	Orn	Lys
Arg1	9.8	9.8	8.8	8.6
Thr2	9.2	10	11	11
Val3	8.6	10	9.2	9.6
**Xaa4**	12	12	10	10
Val5	11	9.8	9.4	11
Gly7	6.8	7.7	15	7.9
Orn8	11	11	9.8	9.9
**Asp9**	9.8	9.4	9.8	9.8
Ile10	10	8.8	10	10
Leu11	10	9.2	9.8	9.9
Gln12	8.8	11	11	11

Table S41. The $^{3}J_{HN\alpha}$ (Hz) Values of the HPTUXaaGlu Peptides.

	Xaa			
Residue	Dap	Dab	Orn	Lys
Arg1	9.4	10	9.2	9.0
Thr2	12	9.0	8.8	9.4
Val3	10	9.8	9.8	9.8
**Xaa4**	14	7.7	11	10
Val5	11	9.6	9.9	9.8
Gly7	15	9.8	9.0	8.8
Orn8	10	8.1	9.4	9.4
**Glu9**	9.9	9.9	8.6	8.8
Ile10	11	11	10	11
Leu11	9.9	8.2	9.9	9.9
Gln12	10	9.8	7.7	9.8

Table S42. The $^{3}J_{HN\alpha}$ (Hz) Values of the HPTUXaaAad Peptides.

	Xaa			
Residue	Dap	Dab	Orn	Lys
Arg1	9.9	9.2	10	9.3
Thr2	13	7.3	10	10
Val3	9.8	11	11	10
**Xaa4**	12	11	10	9.0
Val5	8.9	11	9.9	9.8
Gly7	8.8	10	10	9.0
Orn8	9.6	9.0	8.8	9.6
**Aad9**	7.9	8.6	10	10
Ile10	10	10	10	11
Leu11	8.6	11	8.6	10
Gln12	9.4	10	8.8	9.9

Table S43. The $^{3}J_{HN\alpha}$ (Hz) Values of the HPTFXaaAsp Peptides.

	Xaa			
Residue	Dap	Dab	Orn	Lys
Cys	9.4	11	10	10
Arg1	12	8.4	11	11
Thr2	11	11	11	11
Val3	10	12	11	11
**Xaa4**	11	10	13	12
Val5	12	10	11	12
Gly7	8.1	10	11	5
Orn8	12	10	11	11
**Asp9**	11	8.4	11	11
Ile110	10	11	11	10
Leu11	11	11	11	10
Gln12	11	11	11	10
Cys	11	11	11	12

Table S44. The $^{3}J_{HN\alpha}$ (Hz) Values of the HPTFXaaGlu Peptides.

	Xaa			
Residue	Dap	Dab	Orn	Lys
Cys	11	11	10	11
Arg1	10	11	11	9.6
Thr2	11	10	9.4	11
Val3	11	12	11	12
**Xaa4**	12	12	11	12
Val5	11	10	11	11
Gly7	9.2	10	14	8.1
Orn8	10	11	11	12
**Glu9**	10	10	10	11
Ile110	11	11	11	11
Leu11	9.4	9.6	10	11
Gln12	10	12	11	10
Cys	10	11	11	12

Table S45. The $^3J_{HN\alpha}$ (Hz) Values of the HPTFXaaAad Peptides.

	Xaa			
Residue	Dap	Dab	Orn	Lys
Cys	12	10	10	10
Arg1	11	9.2	11	9.4
Thr2	12	10	10	11
Val3	11	10	10	11
**Xaa4**	11	9.6	9.2	10
Val5	10	11	10	11
Gly7	12	10	14	8.3
Orn8	12	11	11	11
**Aad9**	11	8.9	11	11
Ile110	12	12	10	12
Leu11	9.4	9.9	10	11
Gln12	13	11	10	10
Cys	10	12	11	11

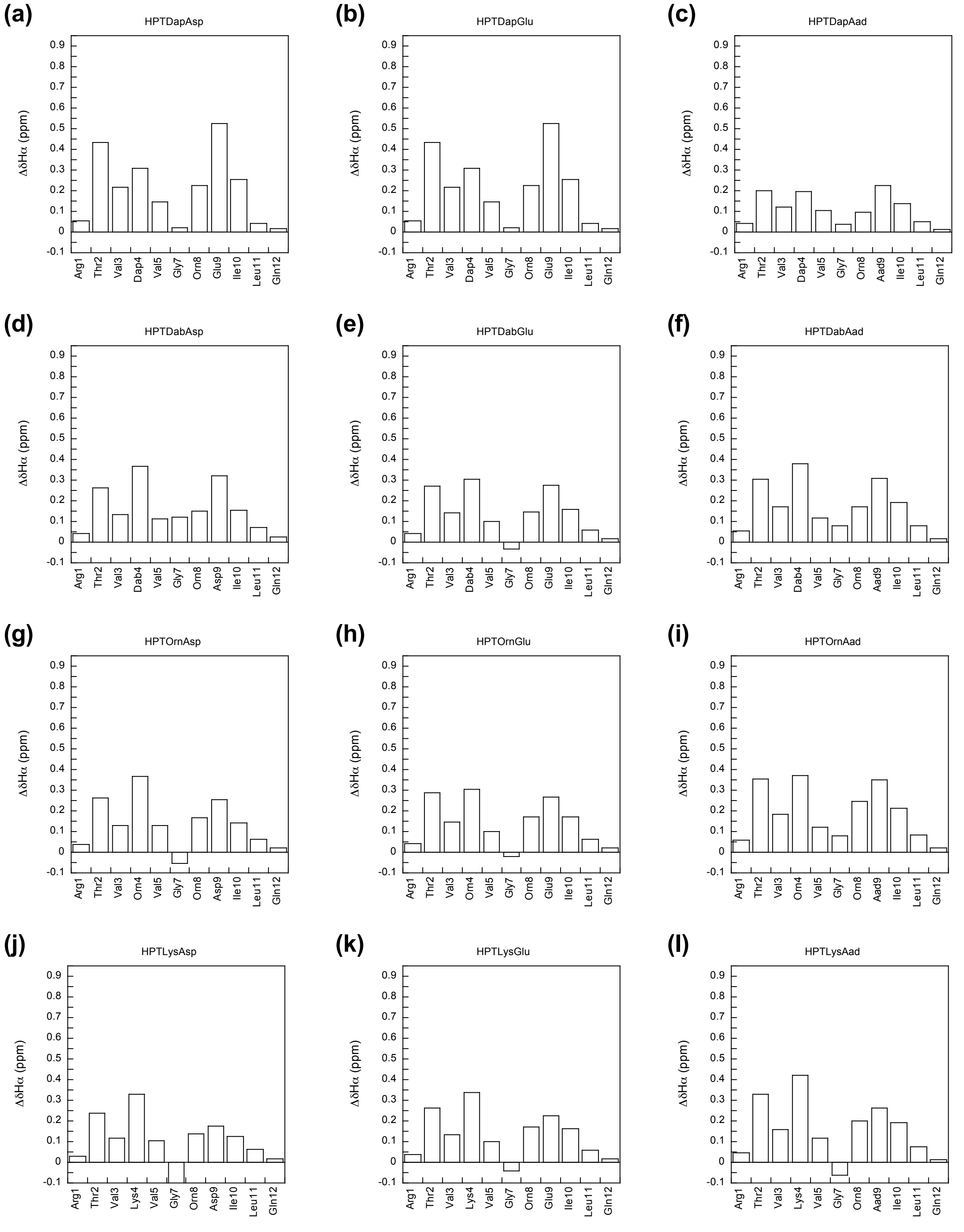

Figure S1. The Hα chemical shift deviation for the residues in the experimental HPTXaaZbb peptides: HPTDapAsp (a), HPTDapGlu (b), HPTDapAad (c), HPTDabAsp (d), HPTDabGlu (e), HPTDabAad (f), HPTOrnAsp (g), HPTOrnGlu (h), HPTOrnAad (i), HPTLysAsp (j), HPTLysGlu (k), HPTLysAad (l).

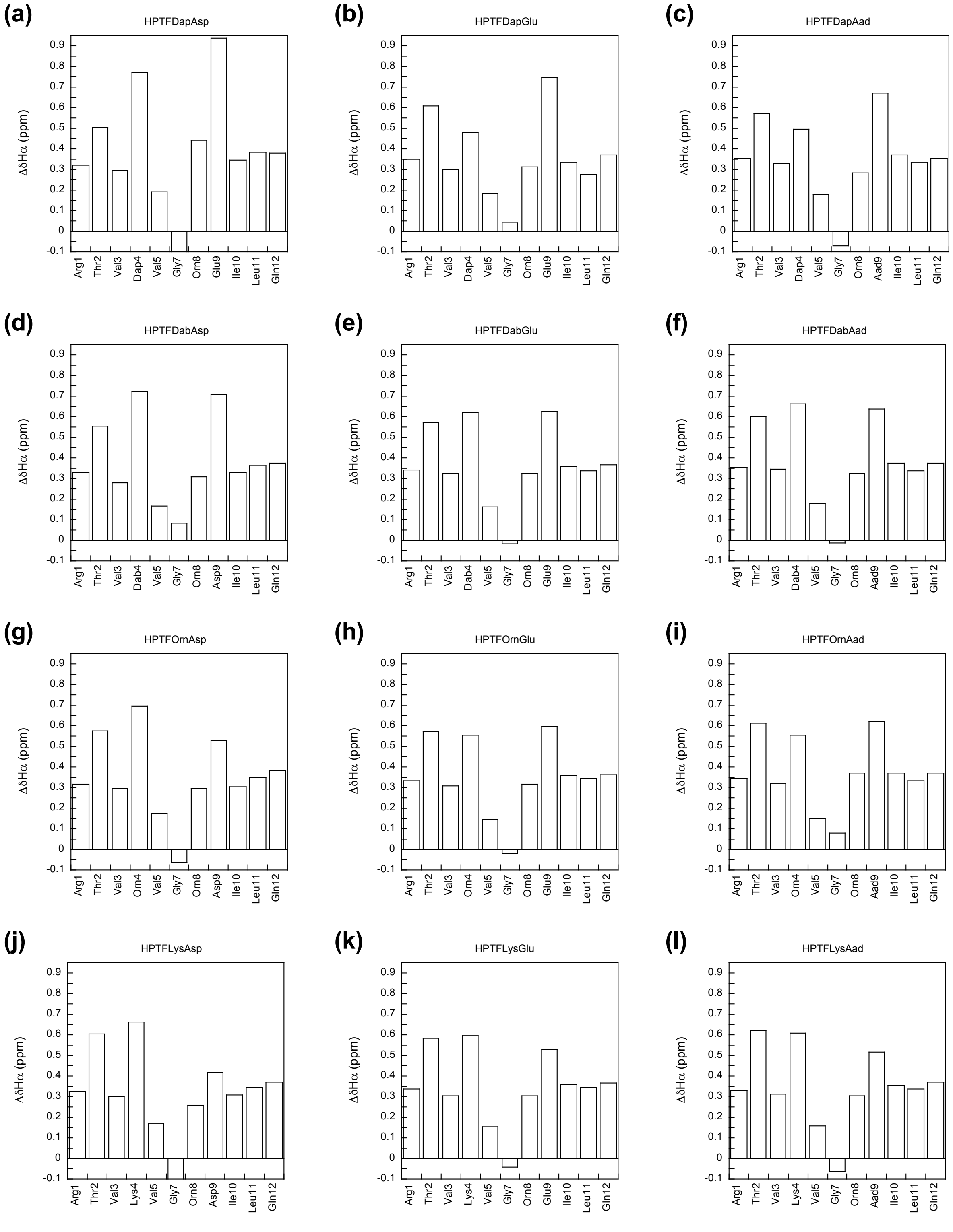

Figure S2. The Hα chemical shift deviation for the residues in the fully folded reference HPTFXaaZbb peptides: HPTFDapAsp (a), HPTFDapGlu (b), HPTFDapAad (c), HPTFDabAsp (d), HPTFDabGlu (e), HPTFDabAad (f), HPTFOrnAsp (g), HPTFOrnGlu (h), HPTFOrnAad (i), HPTFLysAsp (j), HPTFLysGlu (k), HPTFLysAad (l).

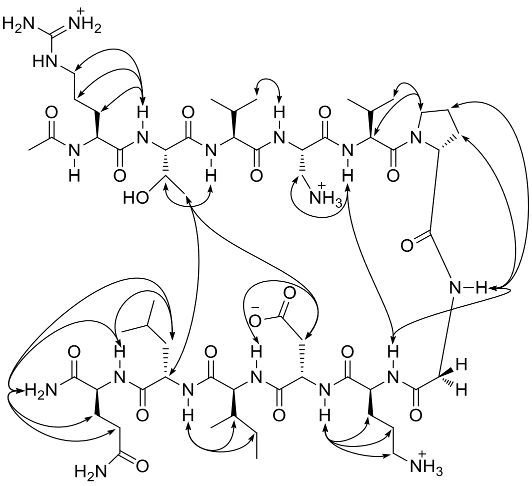

Figure S3. The NOEs in the ROESY spectra of HPTDapAsp involving side chain protons.

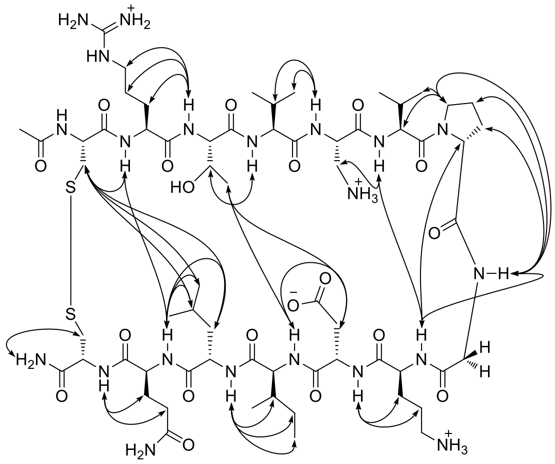

Figure S4. The NOEs in the ROESY spectra of HPTFDapAsp involving side chain protons.

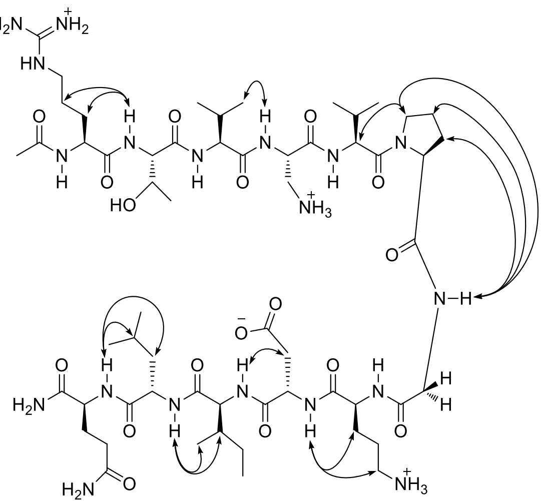

Figure S5. The NOEs in the ROESY spectra of HPTUDapAsp involving side chain protons.

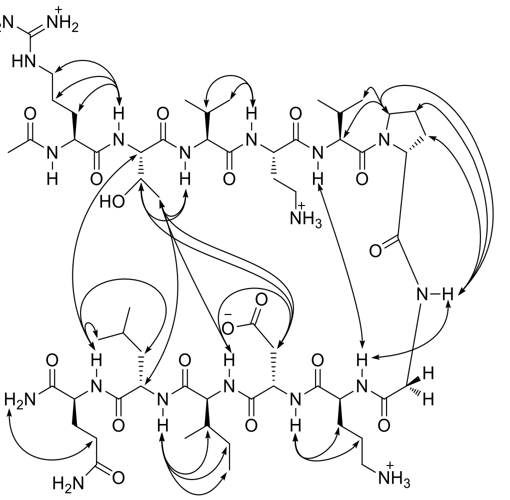

Figure S6. The NOEs in the ROESY spectra of HPTDabAsp involving side chain protons.

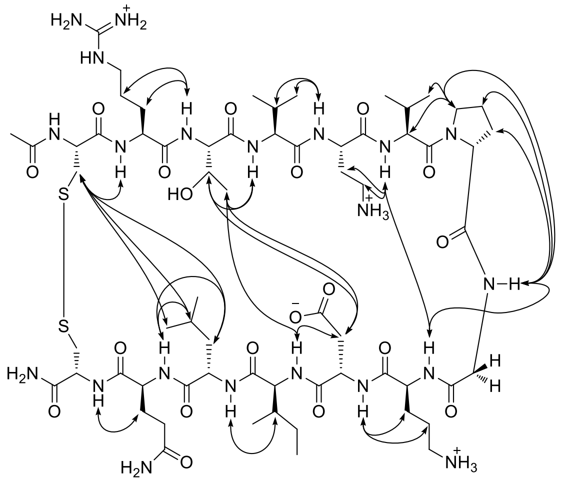

Figure S7. The NOEs in the ROESY spectra of HPTFDabAsp involving side chain protons.

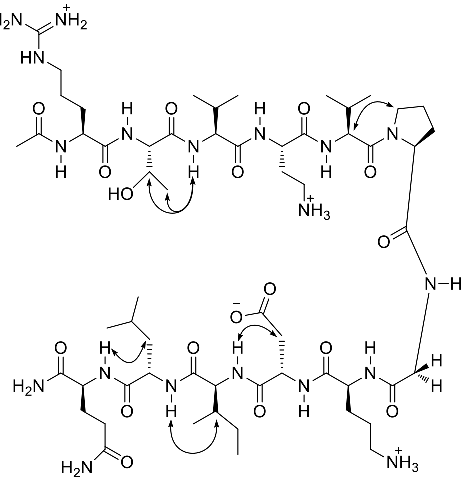

Figure S8. The NOEs in the ROESY spectra of HPTUDabAsp involving side chain protons.

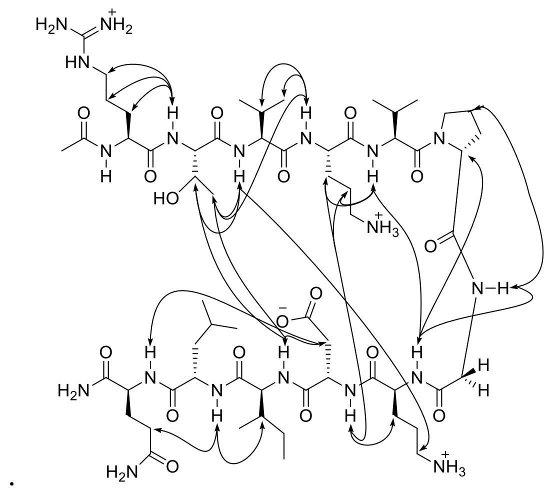

Figure S9. The NOEs in the ROESY spectra of HPTOrnAsp involving side chain protons.

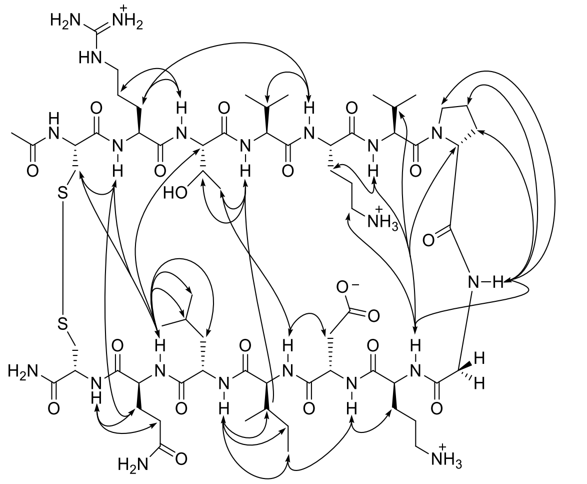

Figure S10. The NOEs in the ROESY spectra of HPTFOrnAsp involving side chain protons.

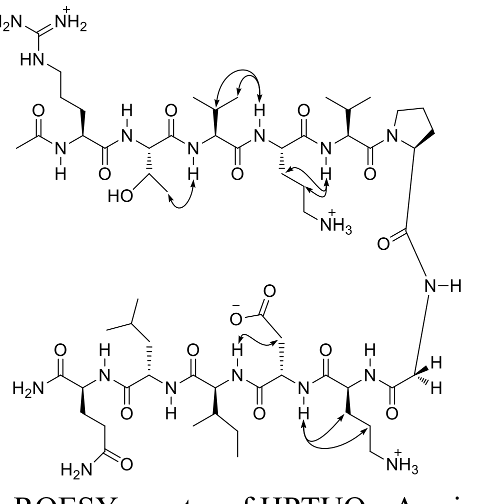

Figure S11. The NOEs in the ROESY spectra of HPTUOrnAsp involving side chain protons.

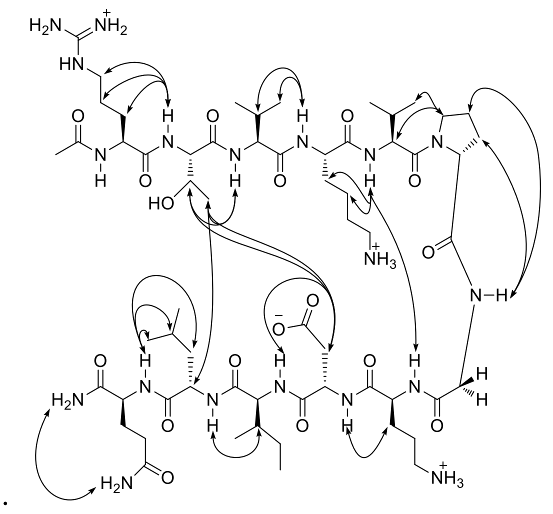

Figure S12. The NOEs in the ROESY spectra of HPTLysAsp involving side chain protons.

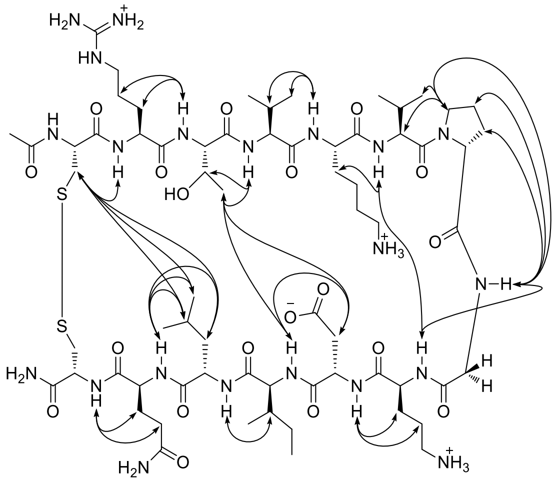

Figure S13. The NOEs in the ROESY spectra of HPTFLysAsp involving side chain protons.

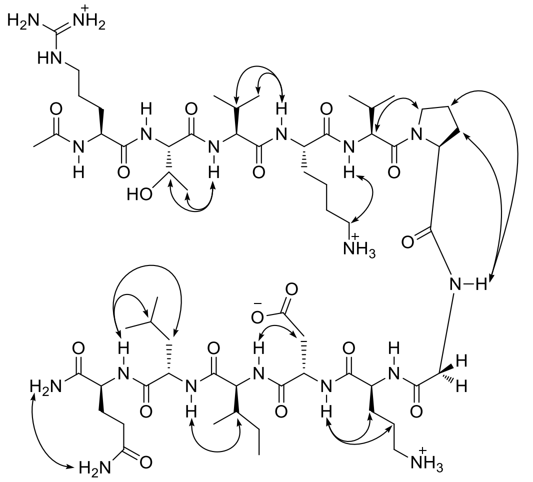

Figure S14. The NOEs in the ROESY spectra of HPTULysAsp involving side chain protons.

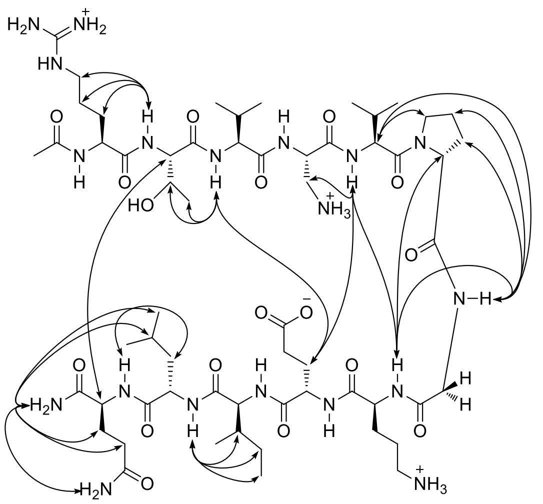

Figure S15. The NOEs in the ROESY spectra of HPTDapGlu involving side chain protons.

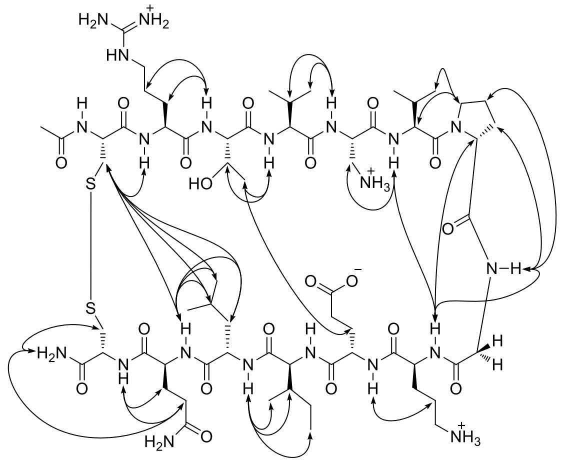

Figure S16. The NOEs in the ROESY spectra of HPTFDapGlu involving side chain protons.

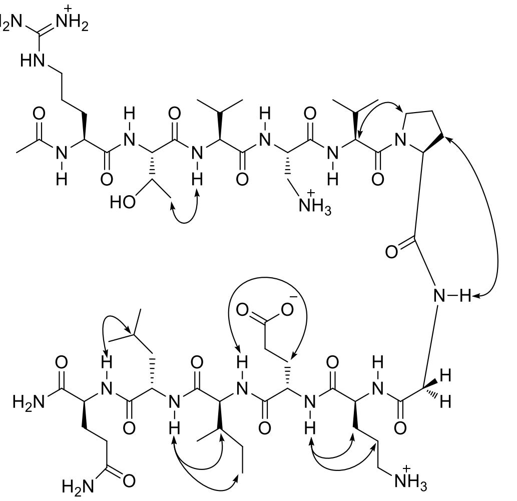

Figure S17. The NOEs in the ROESY spectra of HPTUDapGlu involving side chain protons.

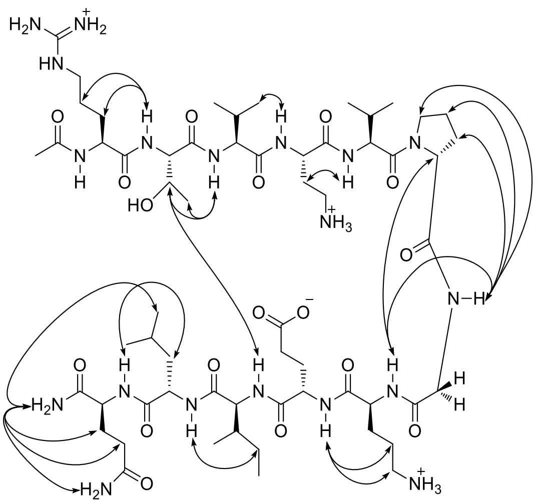

Figure S18. The NOEs in the ROESY spectra of HPTDabGlu involving side chain protons.

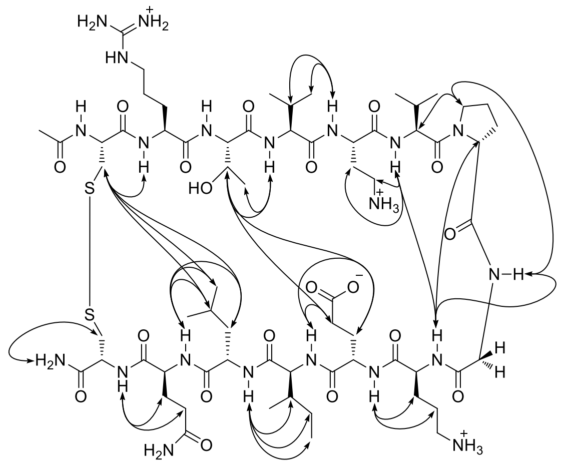

Figure S19. The NOEs in the ROESY spectra of HPTFDabGlu involving side chain protons.

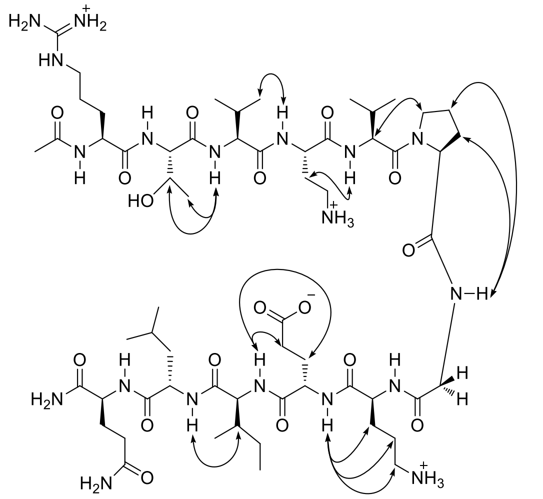

Figure S20. The NOEs in the ROESY spectra of HPTDabGlu involving side chain protons.

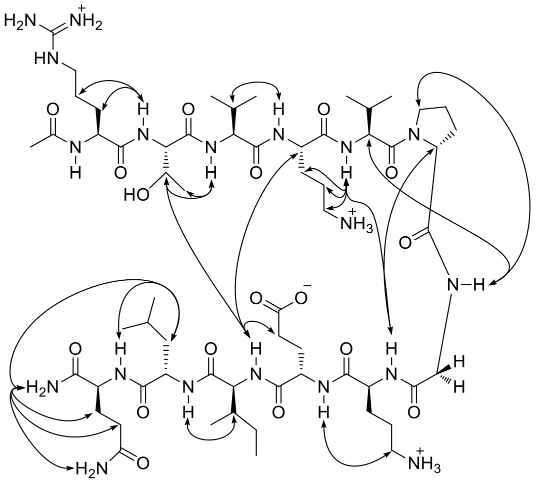

Figure S21. The NOEs in the ROESY spectra of HPTOrnGlu involving side chain protons.

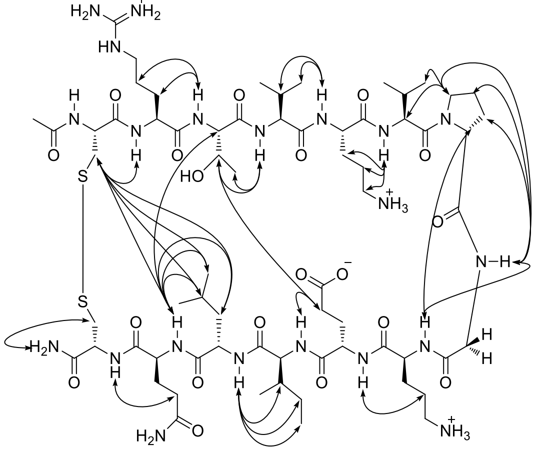

Figure S22. The NOEs in the ROESY spectra of HPTFOrnGlu involving side chain protons.

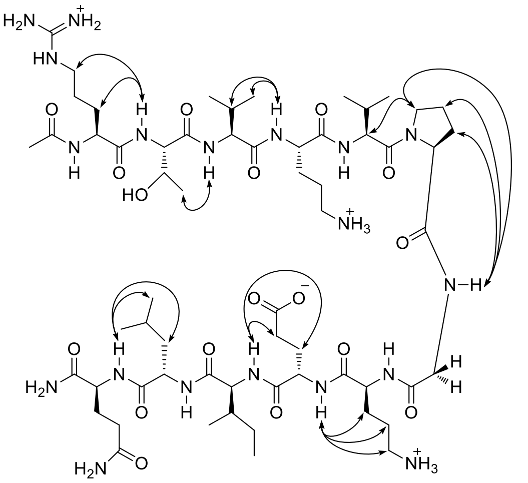

Figure S23. The NOEs in the ROESY spectra of HPTUOrnGlu involving side chain protons.

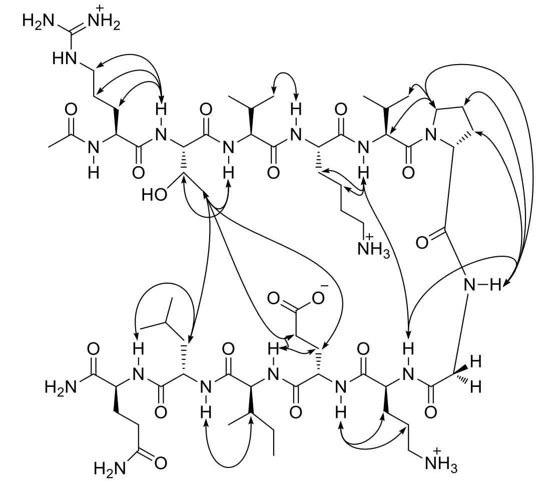

Figure S24. The NOEs in the ROESY spectra of HPTLysGlu involving side chain protons.

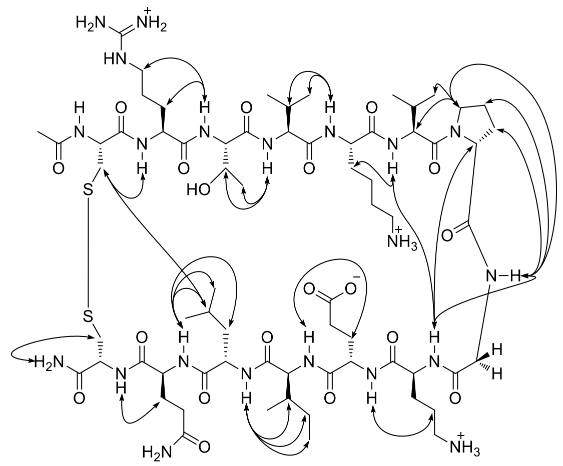

Figure S25. The NOEs in the ROESY spectra of HPTFLysGlu involving side chain protons.

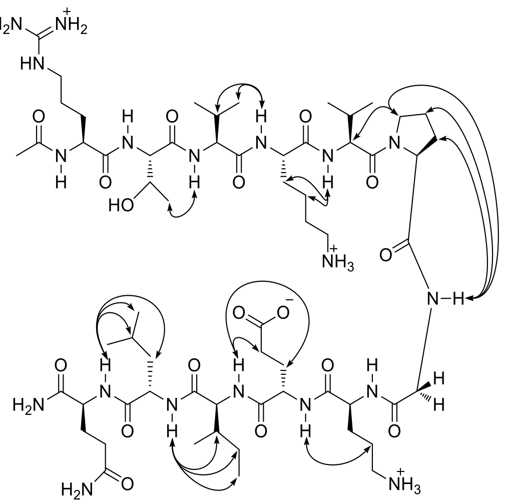

Figure S26. The NOEs in the ROESY spectra of HPTULysGlu involving side chain protons.

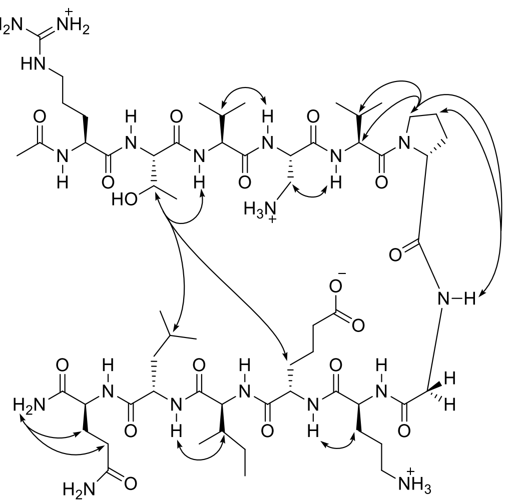

Figure S27. The NOEs in the ROESY spectra of HPTDapAad involving side chain protons.

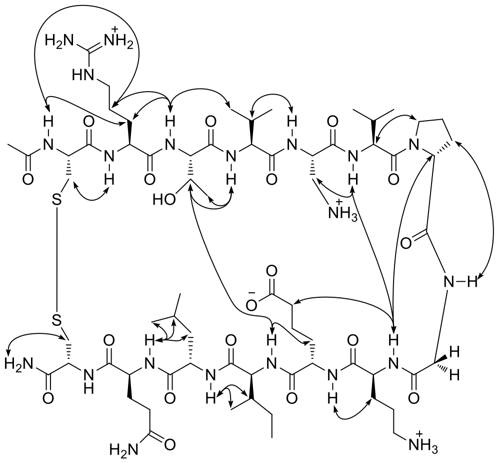

Figure S28. The NOEs in the ROESY spectra of HPTFDapAad involving side chain protons.

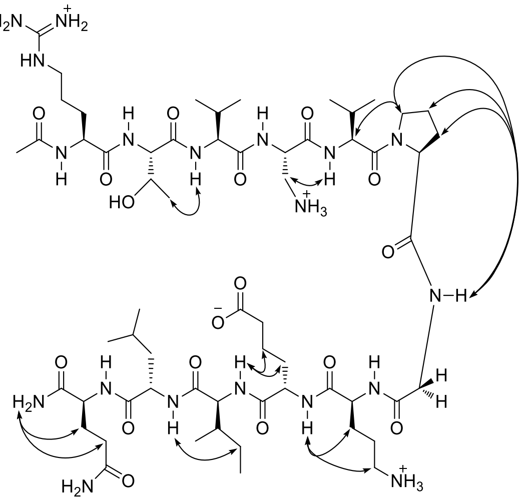

Figure S29. The NOEs in the ROESY spectra of HPTUDapAad involving side chain protons.

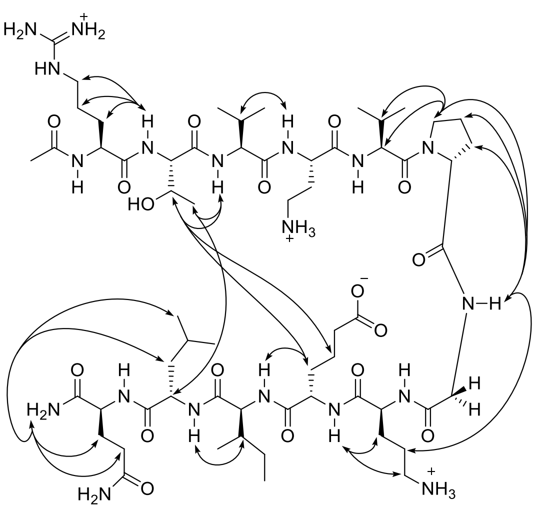

Figure S30. The NOEs in the ROESY spectra of HPTDabAad involving side chain protons.

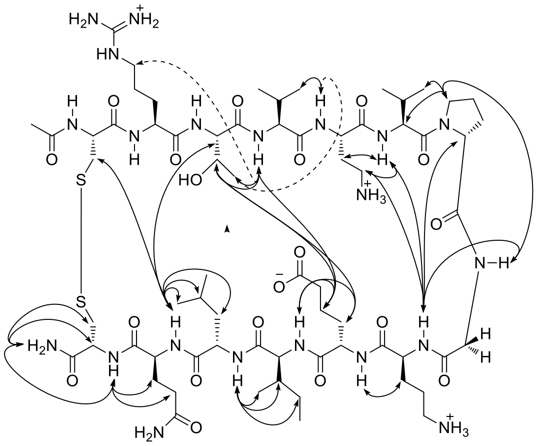

Figure S31. The NOEs in the ROESY spectra of HPTFDabAad involving side chain protons.

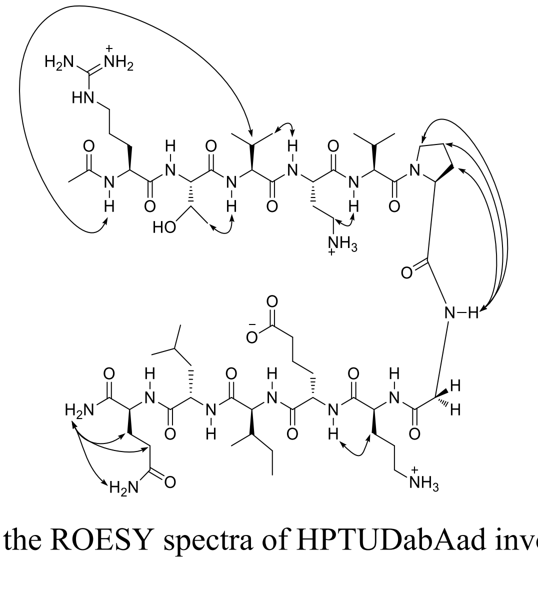

Figure S32. The NOEs in the ROESY spectra of HPTUDabAad involving side chain protons.

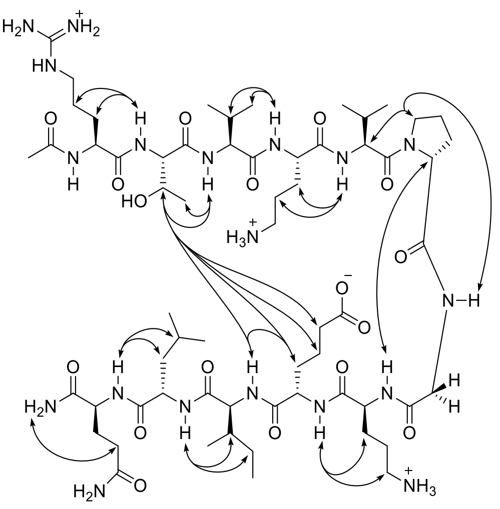

Figure S33. The NOEs in the ROESY spectra of HPTOrnAad involving side chain protons.

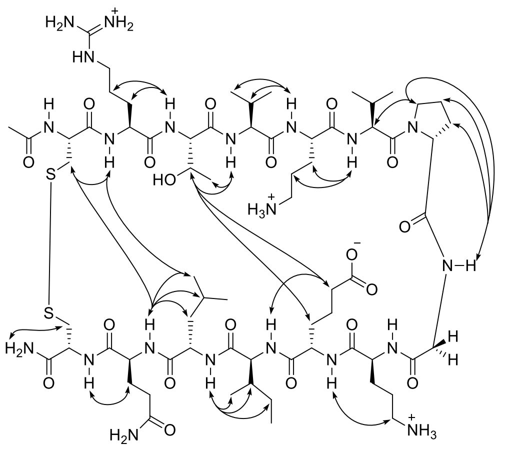

Figure S34. The NOEs in the ROESY spectra of HPTFOrnAad involving side chain protons.

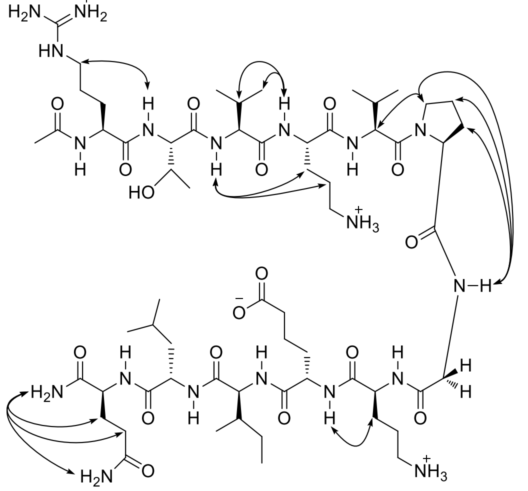

Figure S35. The NOEs in the ROESY spectra of HPTUOrnAad involving side chain protons.

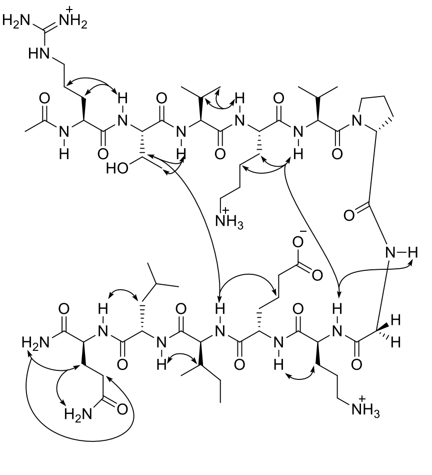

Figure S36. The NOEs in the ROESY spectra of HPTLysAad involving side chain protons.

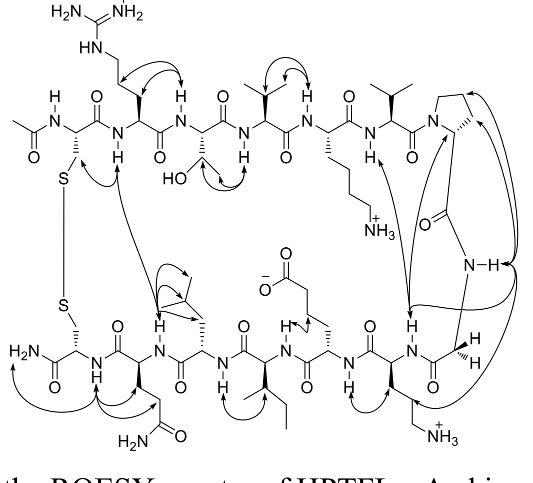

Figure S37. The NOEs in the ROESY spectra of HPTFLysAad involving side chain protons.

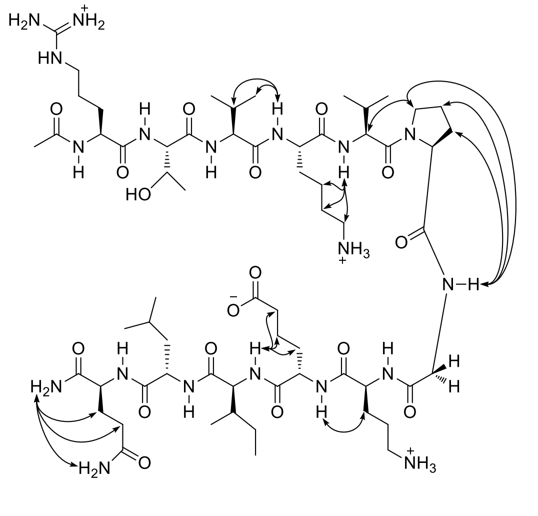

Figure S38. The NOEs in the ROESY spectra of HPTULysAad involving side chain protons.

(a)

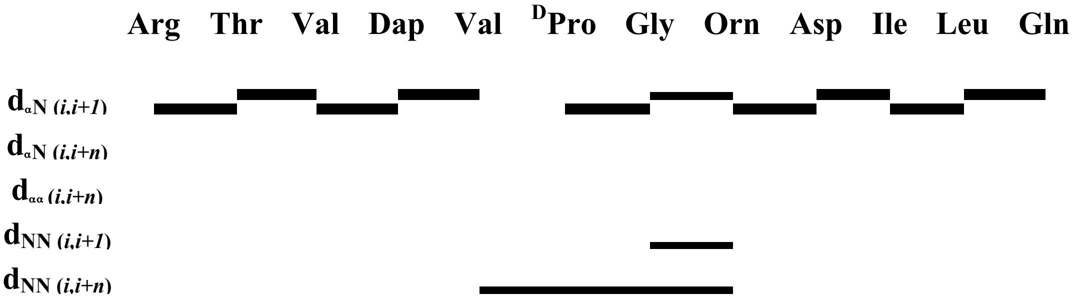

(b)

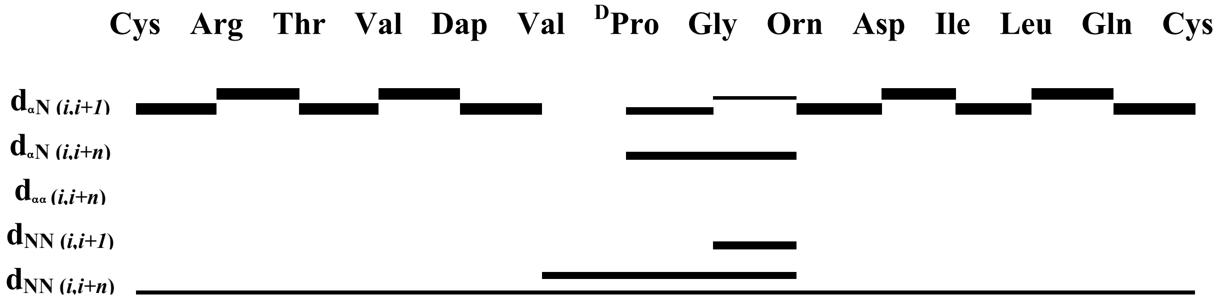

(c)

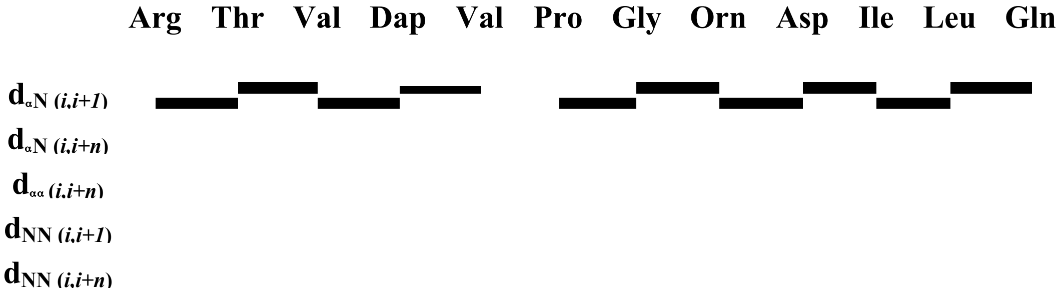

Figure S39. Wüthrich diagrams of the backbone NOE connectivities involving the α-protons and amide protons for peptides HPTDapAsp (a), HPTFDapAsp (b), and HPTUDapAsp (c). The line thickness reflects the NOE intensity and interproton distance; the thicker the line, the stronger the NOE intensity, the shorter the distance.

(a)

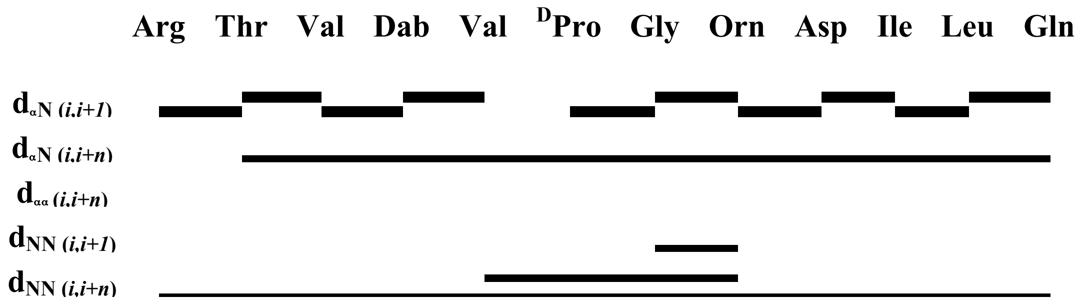

(b)

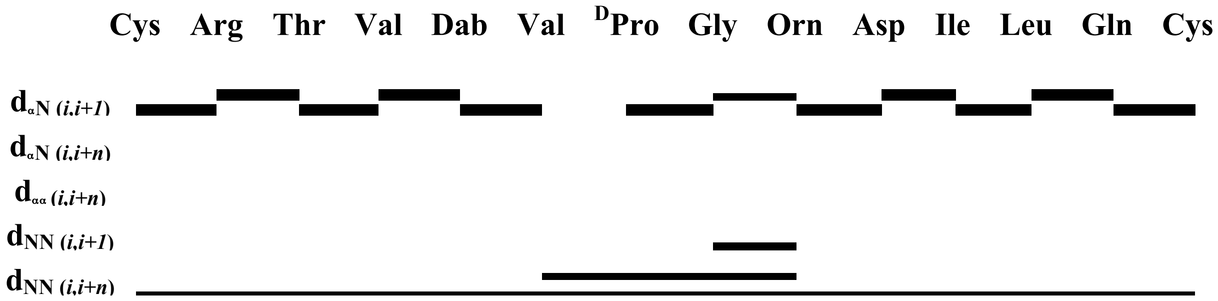

(c)

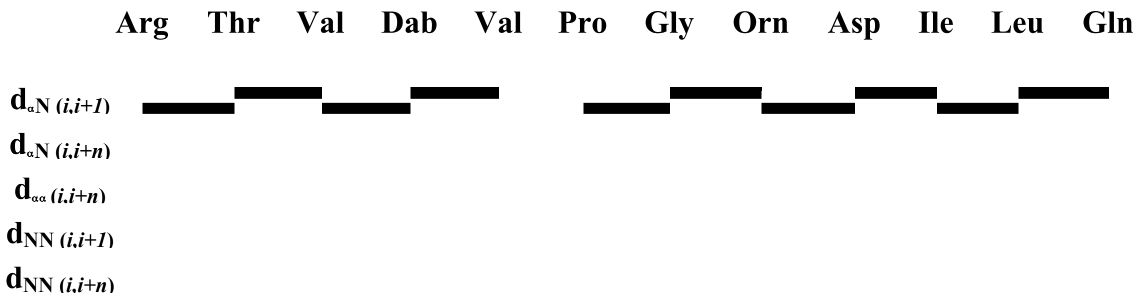

Figure S40. Wüthrich diagrams of the backbone NOE connectivities involving the α-protons and amide protons for peptides HPTDabAsp (a), HPTFDabAsp (b), and HPTUDabAsp (c). The line thickness reflects the NOE intensity and interproton distance; the thicker the line, the stronger the NOE intensity, the shorter the distance.

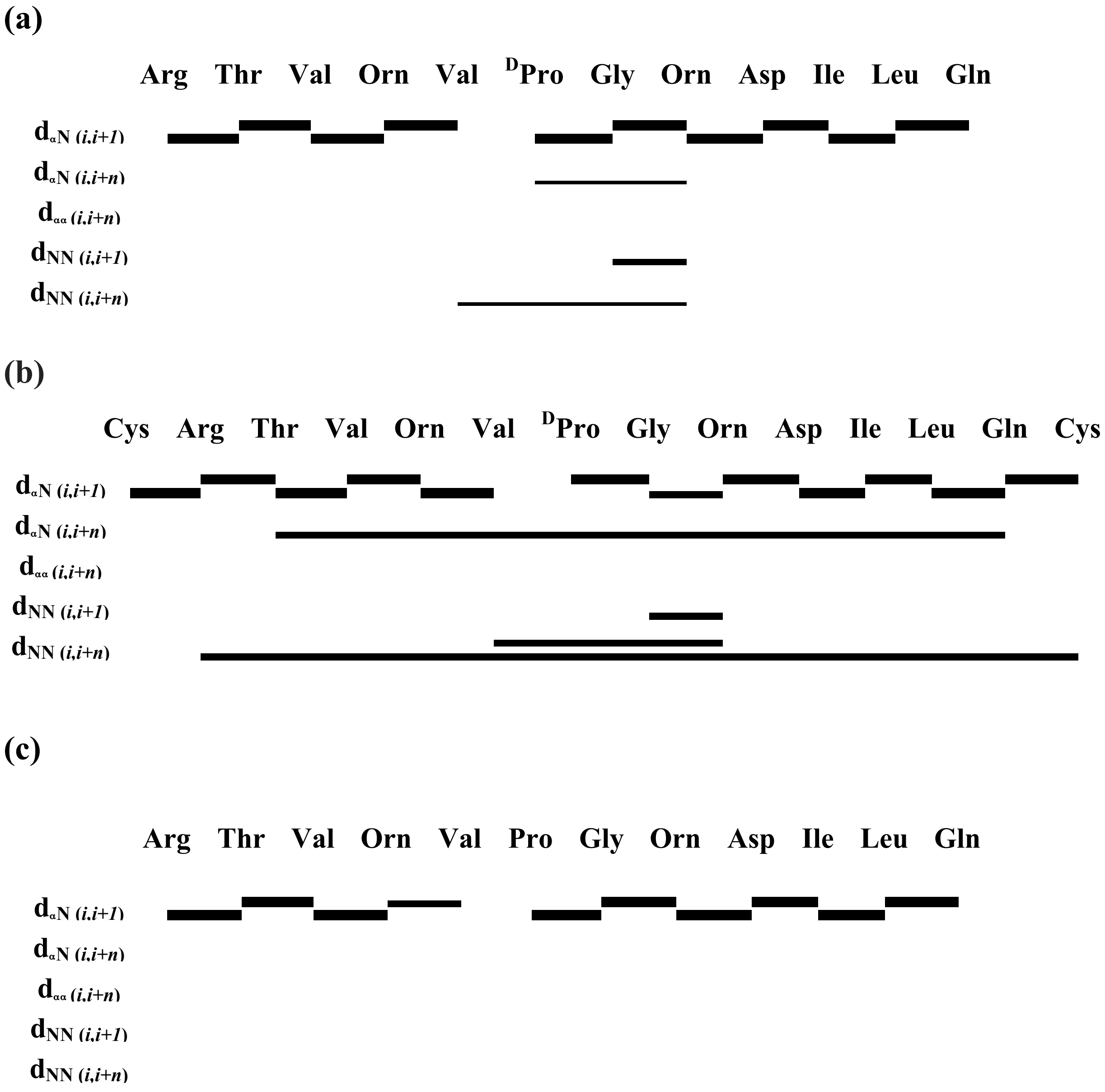

Figure S41. Wüthrich diagrams of the backbone NOE connectivities involving the α-protons and amide protons for peptides HPTOrnAsp (a), HPTFOrnAsp (b), and HPTUOrnAsp (c). The line thickness reflects the NOE intensity and interproton distance; the thicker the line, the stronger the NOE intensity, the shorter the distance.

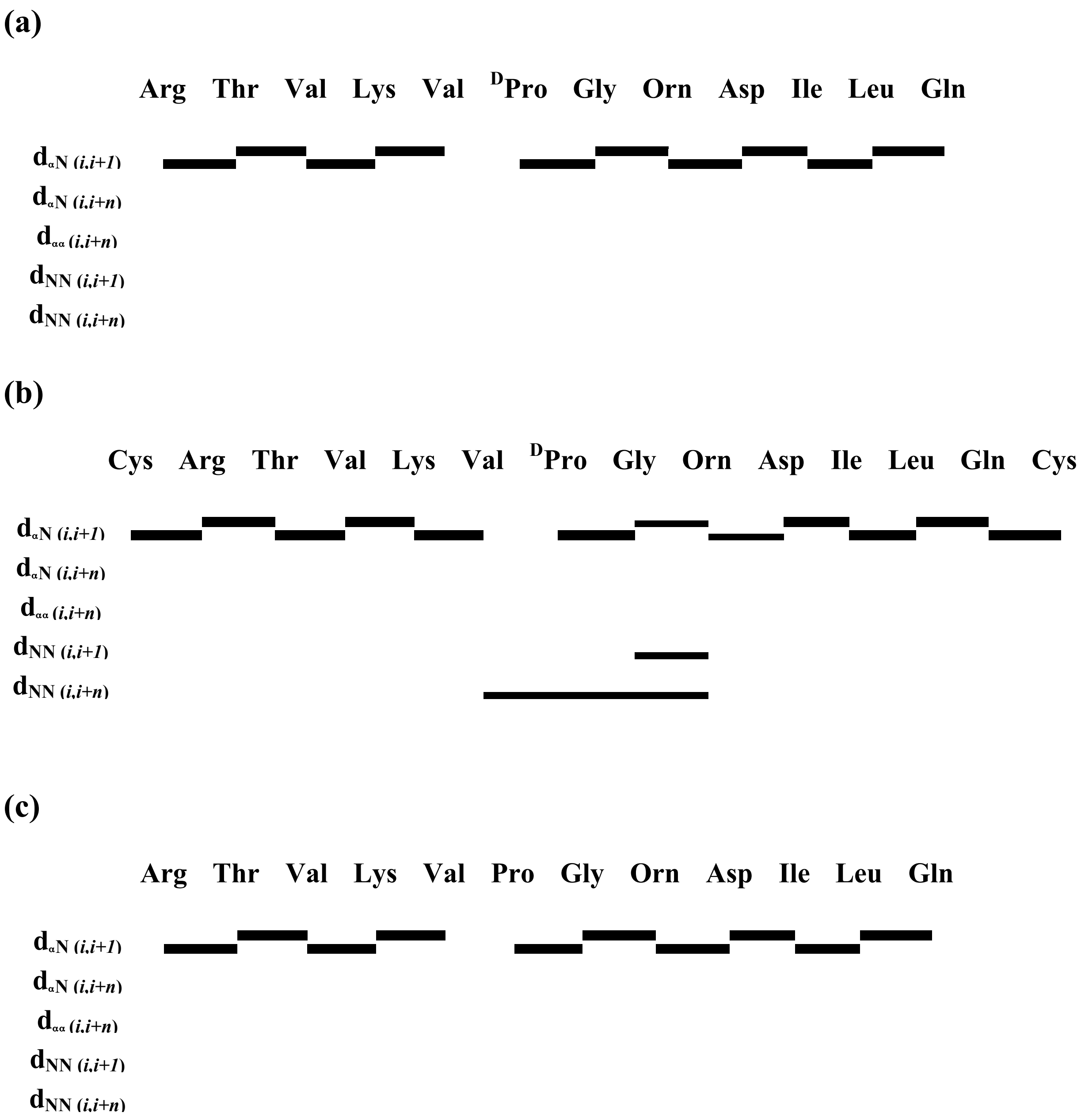

Figure S42. Wüthrich diagrams of the backbone NOE connectivities involving the α-protons and amide protons for peptides HPTLysAsp (a), HPTFLysAsp (b), and HPTULysAsp (c). The line thickness reflects the NOE intensity and interproton distance; the thicker the line, the stronger the NOE intensity, the shorter the distance.

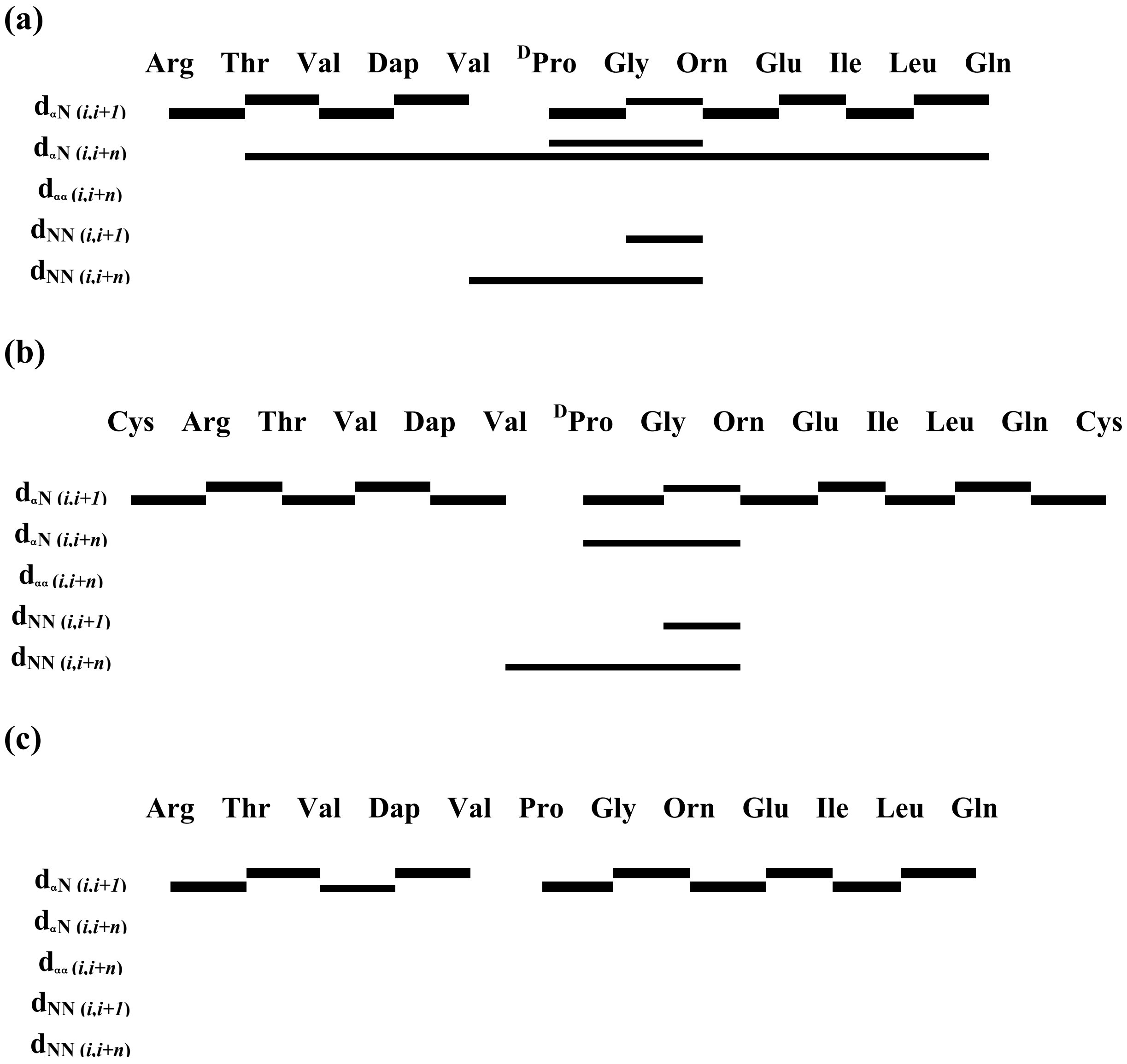

Figure S43. Wüthrich diagrams of the backbone NOE connectivities involving the α-protons and amide protons for peptides HPTDapGlu (a), HPTFDapGlu (b), and HPTUDapGlu (c). The line thickness reflects the NOE intensity and interproton distance; the thicker the line, the stronger the NOE intensity, the shorter the distance.

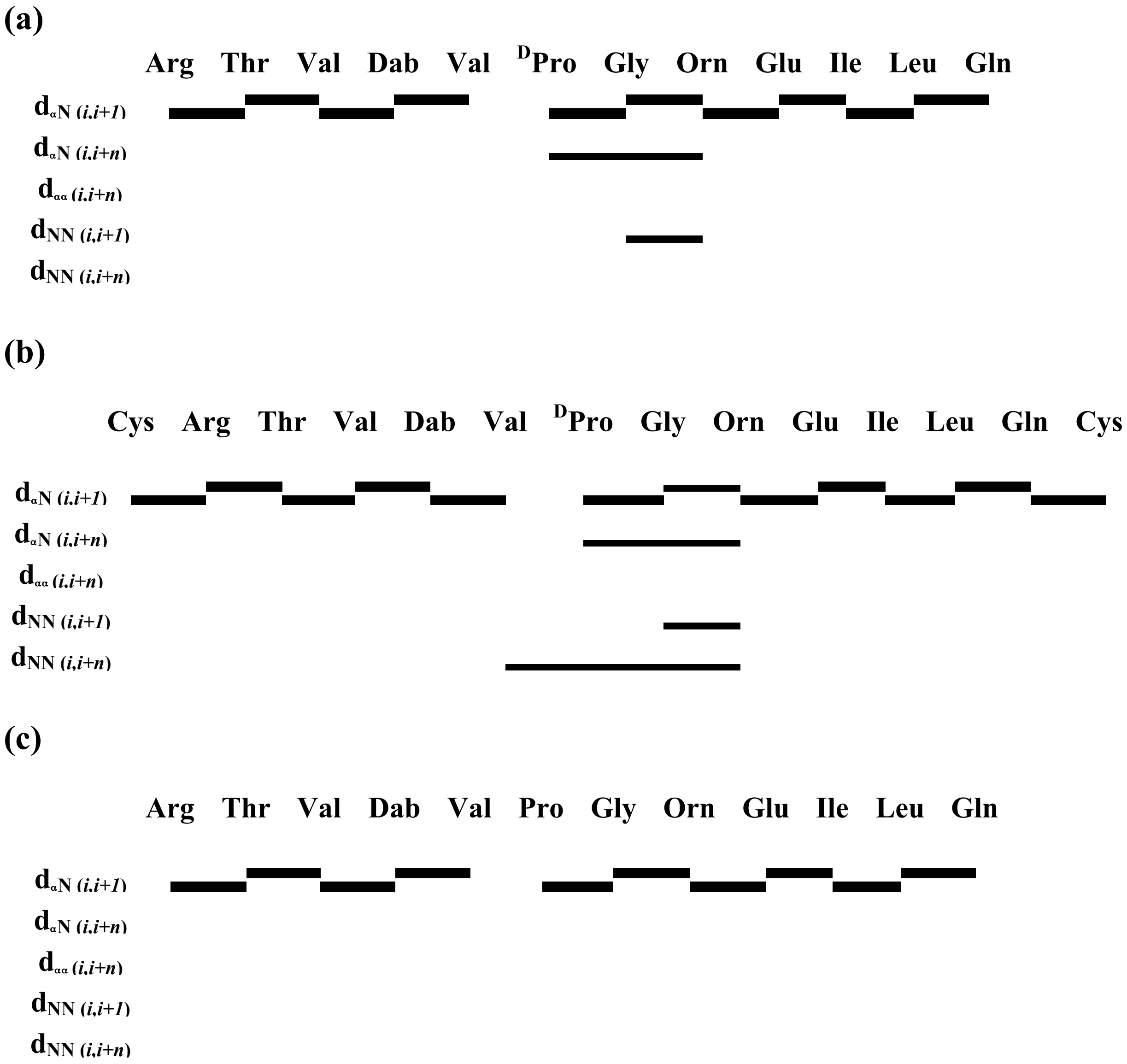

Figure S44. Wüthrich diagrams of the backbone NOE connectivities involving the α-protons and amide protons for peptides HPTDabGlu (a), HPTFDabGlu (b), and HPTUDabGlu (c). The line thickness reflects the NOE intensity and interproton distance; the thicker the line, the stronger the NOE intensity, the shorter the distance.

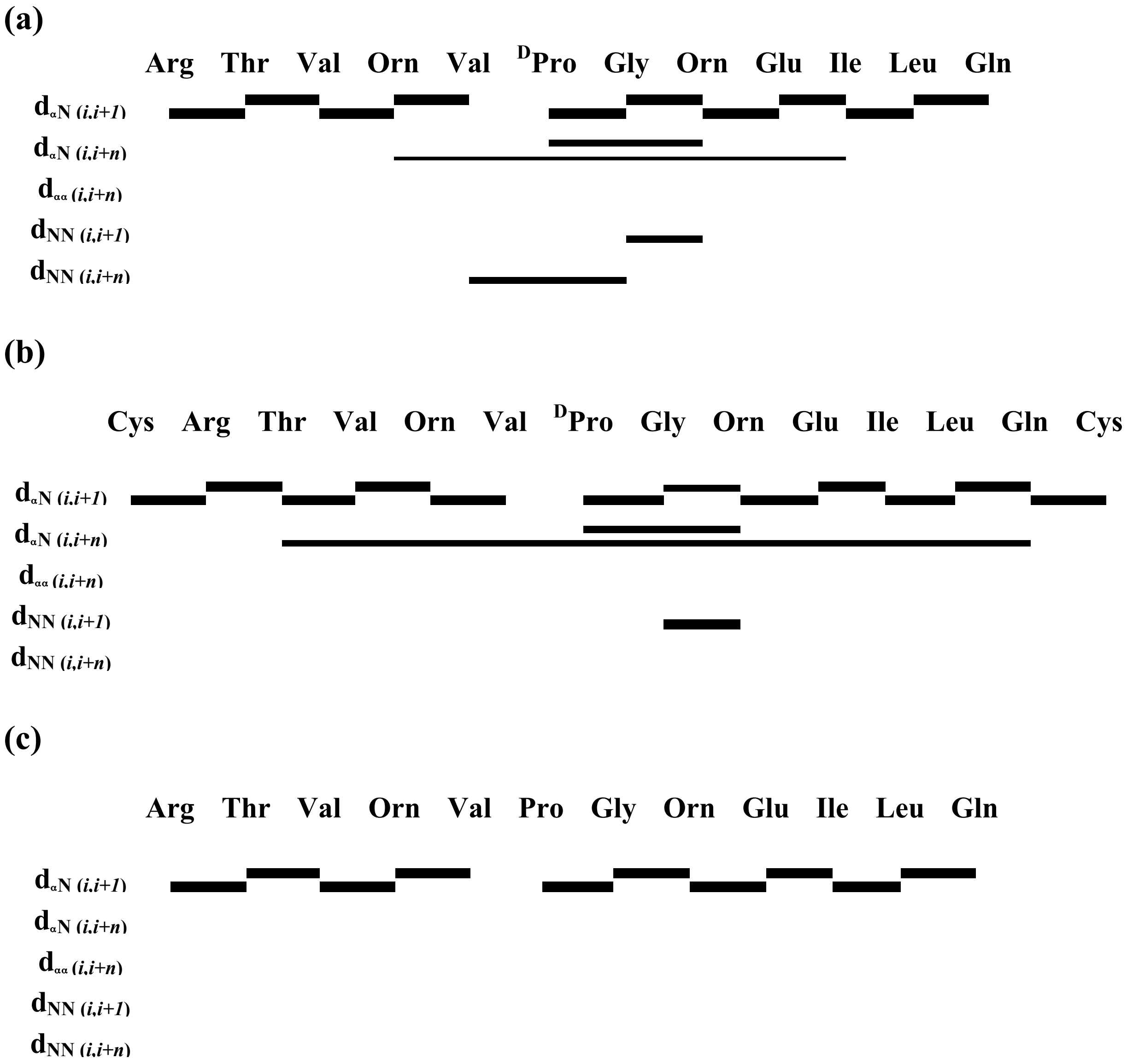

Figure S45. Wüthrich diagrams of the backbone NOE connectivities involving the α-protons and amide protons for peptides HPTOrnGlu (a), HPTFOrnGlu (b), and HPTUOrnGlu (c). The line thickness reflects the NOE intensity and interproton distance; the thicker the line, the stronger the NOE intensity, the shorter the distance.

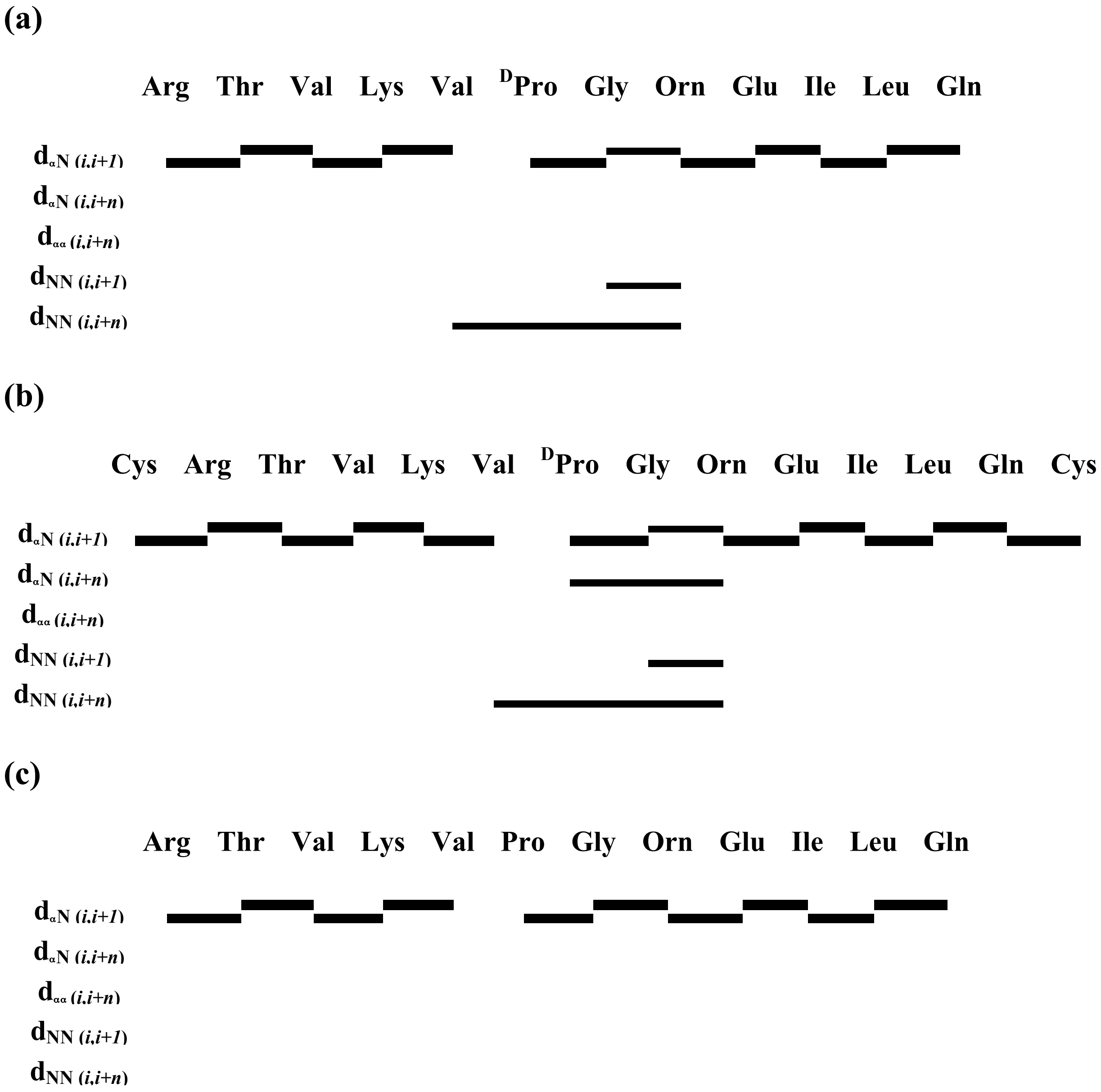

Figure S46. Wüthrich diagrams of the backbone NOE connectivities involving the α-protons and amide protons for peptides HPTLysGlu (a), HPTFLysGlu (b), and HPTULysGlu (c). The line thickness reflects the NOE intensity and interproton distance; the thicker the line, the stronger the NOE intensity, the shorter the distance.

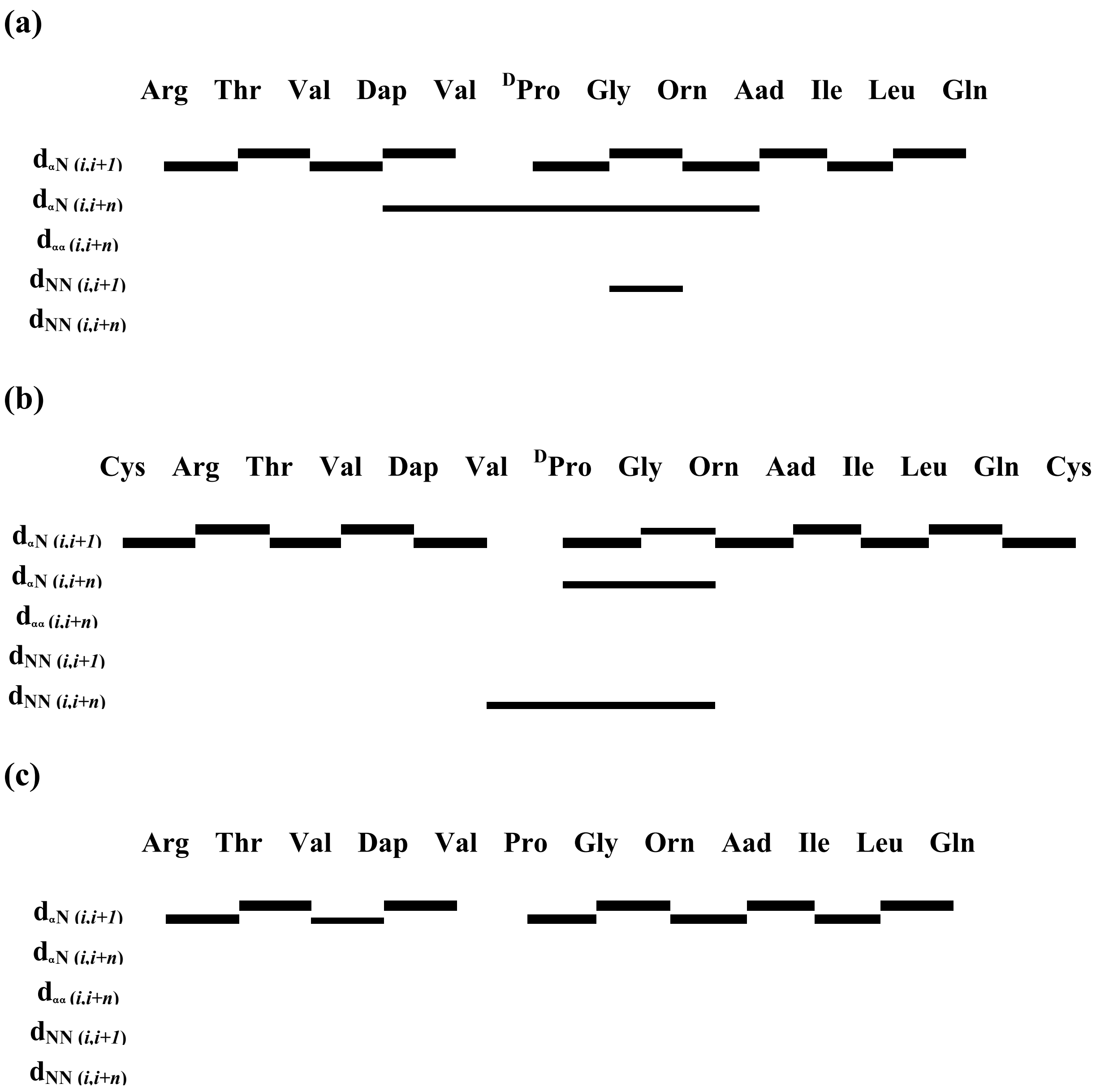

Figure S47. Wüthrich diagrams of the backbone NOE connectivities involving the α-protons and amide protons for peptides HPTDapAad (a), HPTFDapAad (b), and HPTUDapAad (c). The line thickness reflects the NOE intensity and interproton distance; the thicker the line, the stronger the NOE intensity, the shorter the distance.

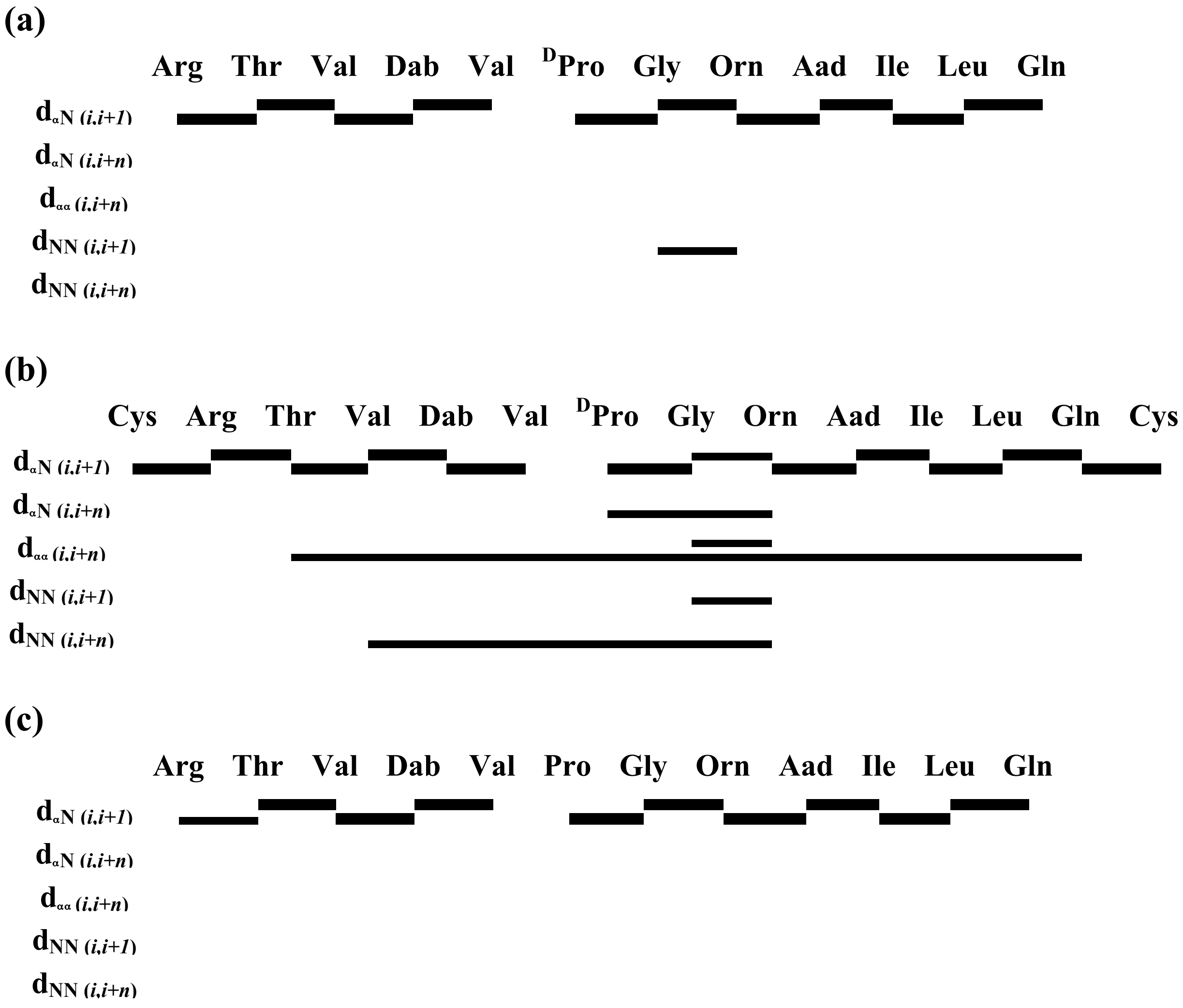

Figure S48. Wüthrich diagrams of the backbone NOE connectivities involving the α-protons and amide protons for peptides HPTDabAad (a), HPTFDabAad (b), and HPTUDabAad (c). The line thickness reflects the NOE intensity and interproton distance; the thicker the line, the stronger the NOE intensity, the shorter the distance.

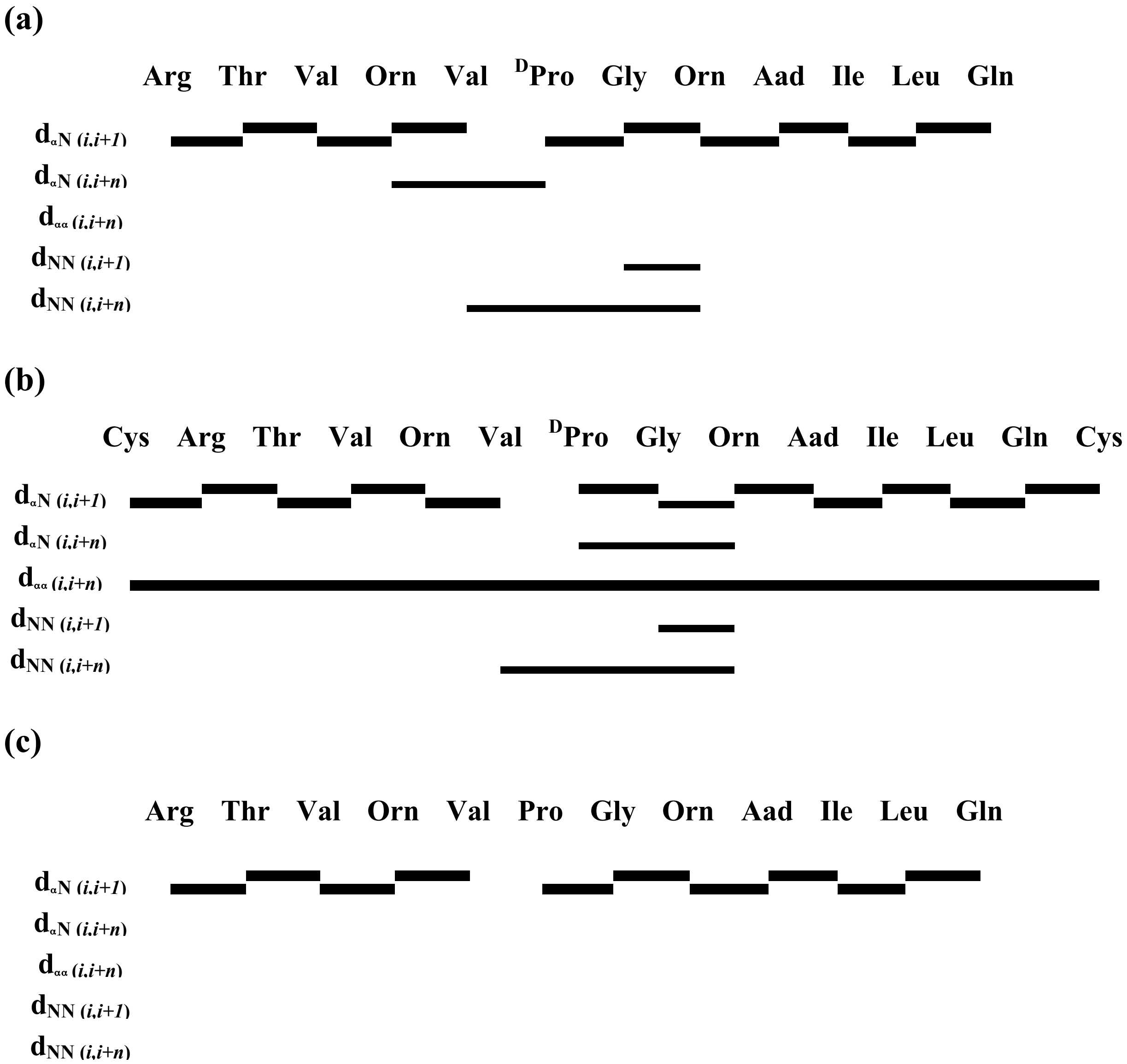

Figure S49. Wüthrich diagrams of the backbone NOE connectivities involving the α-protons and amide protons for peptides HPTOrnAad (a), HPTFOrnAad (b), and HPTUOrnAad (c). The line thickness reflects the NOE intensity and interproton distance; the thicker the line, the stronger the NOE intensity, the shorter the distance.

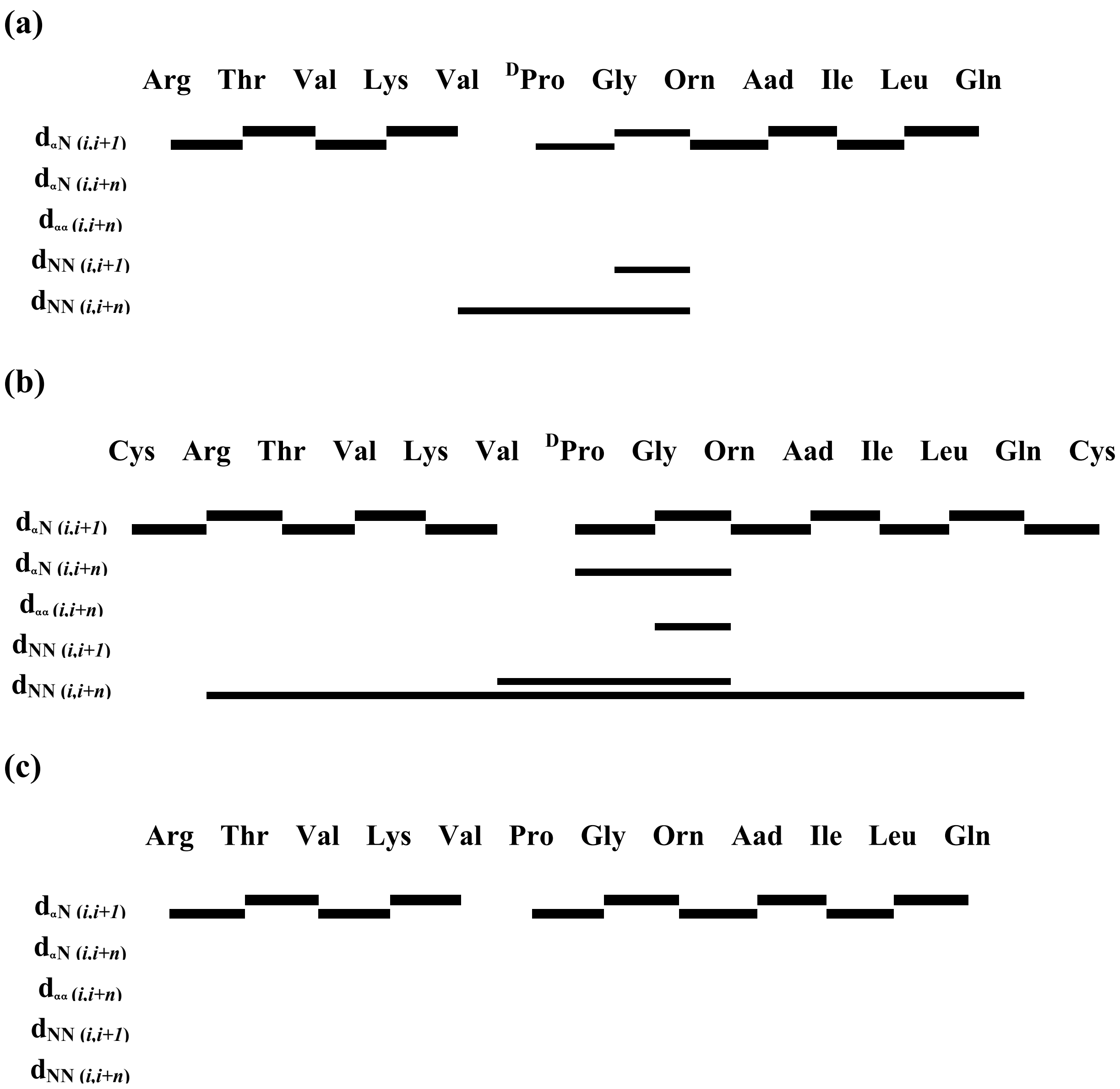

Figure S50. Wüthrich diagrams of the backbone NOE connectivities involving the α-protons and amide protons for peptides HPTLysAad (a), HPTFLysAad (b), and HPTULysAad (c). The line thickness reflects the NOE intensity and interproton distance; the thicker the line, the stronger the NOE intensity, the shorter the distance.

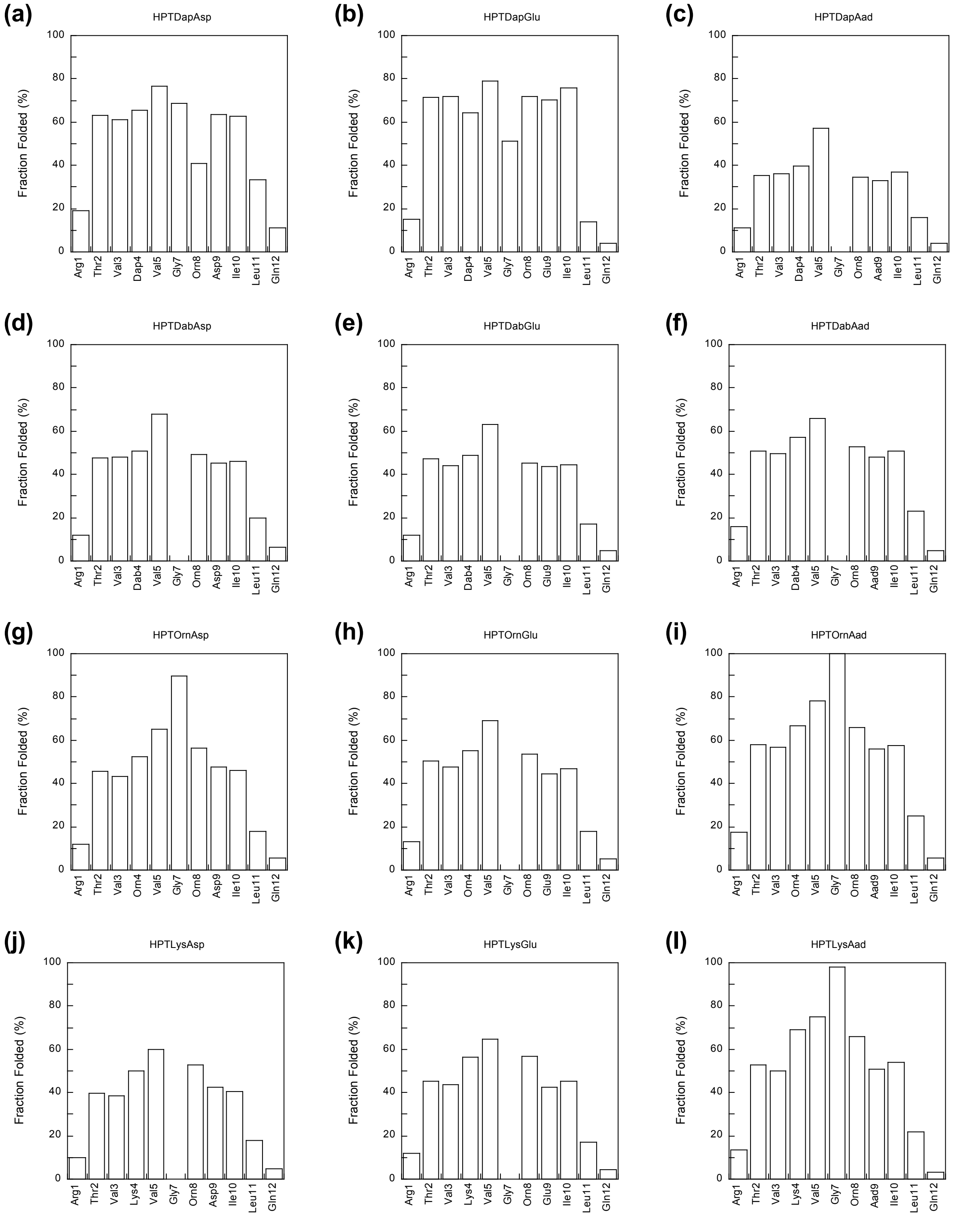

Figure S51. The fraction folded of the residues in HPTXaaZbb peptides. HPTDapAsp (a), HPTDapGlu (b), HPTDapAad (c), HPTDabAsp (d), HPTDabGlu (e), HPTDabAad (f), HPTOrnAsp (g), HPTOrnGlu (h), HPTOrnAad (i), HPTLysAsp (j), HPTLysGlu (k), HPTLysAad (l).

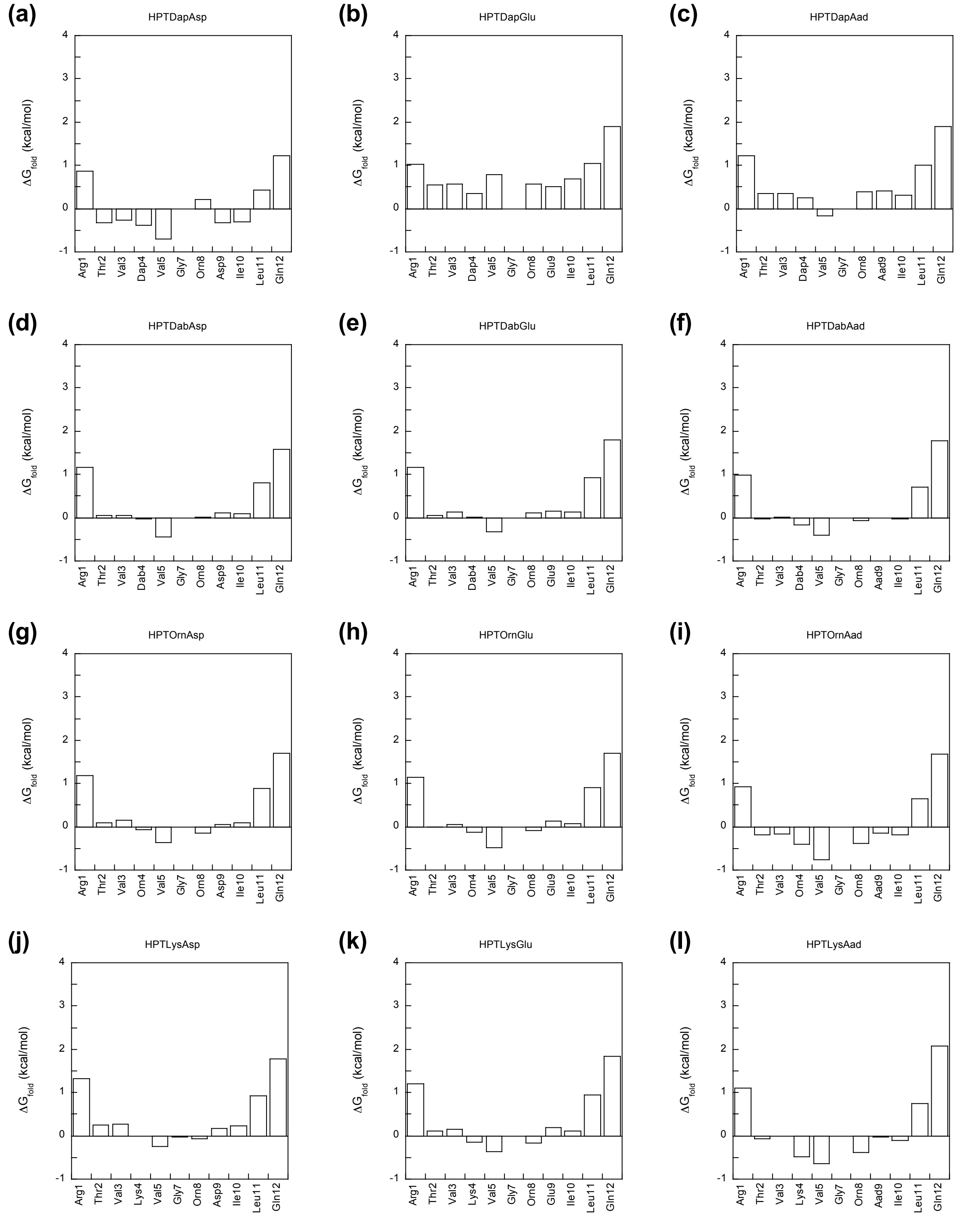

Figure S52. The ΔG_{fold} of the residues in HPTXaaZbb peptides. HPTDapAsp (a), HPTDapGlu (b), HPTDapAad (c), HPTDabAsp (d), HPTDabGlu (e), HPTDabAad (f), HPTOrnAsp (g), HPTOrnGlu (h), HPTOrnAad (i), HPTLysAsp (j), HPTLysGlu (k), HPTLysAad (l).

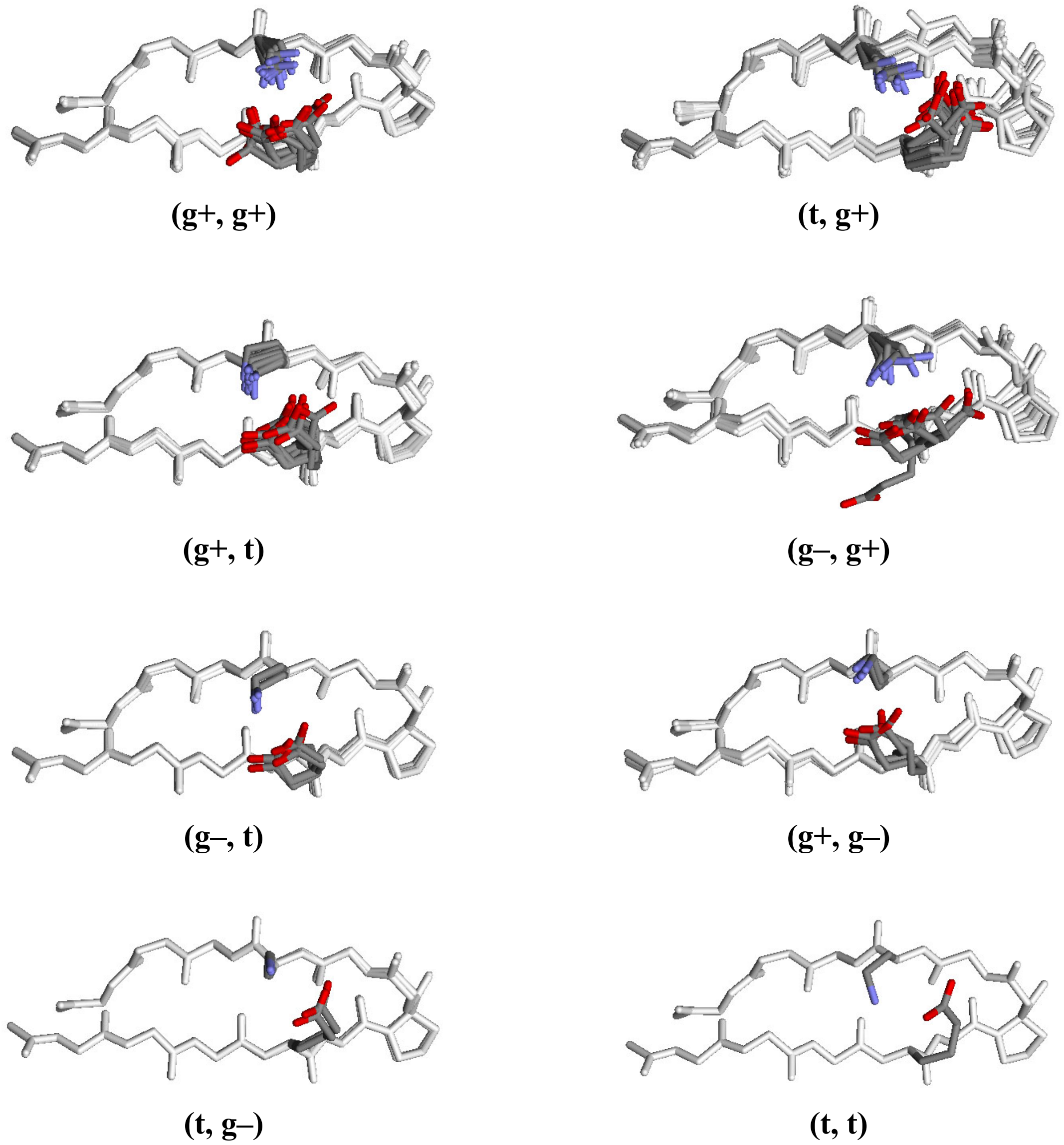

Figure S53. The low energy conformations for peptide HPTAadDab from the side chain conformational analysis by molecular mechanics calculations.

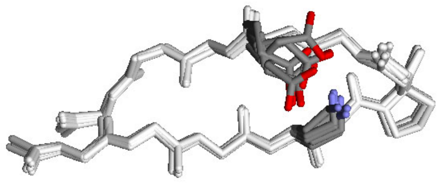

(t, g+)

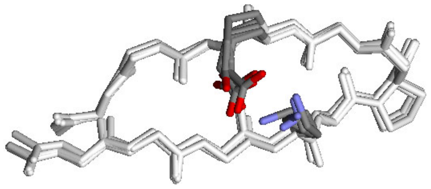

(g+, t)

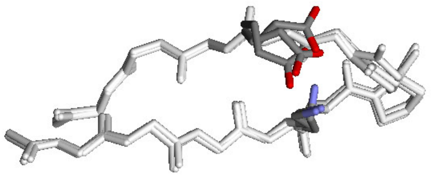

(g+, g+)

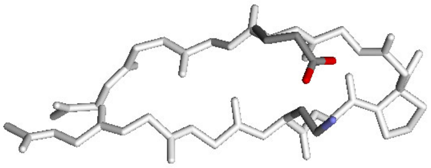

(g–, g+)

Figure S54. The low energy conformations for peptide HPTDabAad from the side chain conformational analysis by molecular mechanics calculations.

Material and Methods

General Section

All reagents and solvents were used without purification. Diisopropylethylamine (DIEA), piperidine, trifluoroacetic acid (TFA), acetic anhydride (Ac_2O) were purchased from Acros. N_α-Fmoc-N_β-Boc-L-2,3-diaminopropionic acid, N_α-Fmoc-N_γ-Boc-L-2,4-diaminobutyric acid, N_α-Fmoc-D-proline, dimethylformamide (DMF), methanol, and acetonitrile were purchased from Merck. N_α-Fmoc-aminoadipic acid-δ-t-butyl ester was from BaChem. N_α-Fmoc-amino acids, 1-hydroxybenzotriazole (HOBt), 2-(1H-Benzotriazole-1-yl)-1, 1, 3, 3 -tetramethyluronium hexafluorophosphate (HBTU), NovaSyn® TGR resin were from NovaBiochem. Hexanes were from Duksan. Analytical reverse phase (RP)-HPLC was performed on an Agilent 1200 series chromatography system using a Vydac C_{18} column (4.6 mm diameter, 250 mm length). Preparative RP-HPLC was performed on Waters Breeze chromatography system using a Seppak® plus short tC_{18} cartridges, Vydac C_4 or C_{18} column (22 mm diameter, 250 mm length) Mass spectrometry of the peptides was performed on a matrix-assisted laser desorption ionization time-of-fight (MALDI-TOF) (Bruker BIFLEX) using α-cyano-4-hydroxycinnamic acid as the matrix. 2-Dimensional nuclear magnetic resonance spectroscopy experiments were performed on the Bruker AV III 800MHz spectrometer.

Peptide Synthesis

The peptides were synthesized by solid phase peptide synthesis using Fmoc-based chemistry [1, 2]. NovaSyn® TGR resin (0.050 mmol) was swollen in N, N-dimethylformamide (DMF, 3 mL) for 30 minutes. A mixture of 3 equivalents of the appropriately protected Fmoc-amino acid, HOBt, and HBTU was dissolved in DMF (1 mL). Diisopropylethylamine (DIEA, 8 equivalents) was then added to the solution and mixed thoroughly. The solution was then applied to the resin. The vial that contained the solution was rinsed with DMF (2x1 mL) and added to the reaction. The first coupling was carried out for 8 hours. The 8th to 14th residues were coupled for 1.5 hours. Other residues were coupled for 45 minutes. The residue with β-branching and the residue after it were coupled with double the time. After each coupling, the resin was washed with DMF (5 mL, 5x1 min). The Fmoc-group was then removed by 20% piperidine/DMF (5 mL, 3x8 min). After the final residue was coupled, a solution of acetic anhydride (20 equivalents), DIEA (20 equivalents), and DMF (3 mL) was added to resin for capping. The reaction was shaken for 2 hours.

Peptides were deprotected and cleaved off the resin by treating the resin with 5 mL 95:5 trifluoroacetic acid (TFA)/triisopropylsilane and shaken for 2 hours. For Cys-containing peptides, 5 mL 90:5:5 trifluoroacetic acid (TFA)/triisopropylsilane/ethanedithiol was used instead. The solution was then filtered through glass wool and the resin was washed with TFA (3x1.5 mL). The combined filtrate was evaporated gently by an air pump (nitrogen gas was used for the Cys-containing peptides). The resulting material was washed with hexanes

(3x3 mL), dissolved in water, and lyophilized. The peptide (1 mg/ mL, aqueous solution) was analyzed using analytical RP-HPLC on a 25 cm C_{18} column (dia 4.6 mm) with flow rate 1 mL/min, temperature 25°C, linear 1 %/ min gradient from 100% A to 0% A (solvent A: 99.9% water, 0.1% TFA; solvent B: 90% acetonitrile, 10% water, 0.1% TFA). The disulfide bond of the Cys-containing HPTFXaaZbb peptides were formed via charcoal mediated air oxidation [3]. Peptides were purified to higher than 95% purity by Sep-Pak® Plus Short tC18 cartridges using an appropriate percentage of B solvent and by reverse phase HPLC using a preparative C_4 and C_{18} columns with flow rate 10 mL·min^{-1}, temperature 25°C, linear 0.5 %·min^{-1} gradient. Appropriate linear gradients of solvent A and solvent B were used for each peptide to place the retention time for the desired peptide between 20 and 30 minutes. These gradients are listed individually for each peptide (vide infra); for example, PLG15_25 was used to purify HPTDapAsp using a C_{18} column, representing the linear gradient from 15 % B to 25 % B (flow rate 10 mL·min^{-1}, temperature 25°C, linear 0.5 %·min^{-1} gradient). The identity of the peptide was confirmed by MALDI-TOF.

HPTDapAsp (Ac-Arg Thr Val Dap Val ^{D}Pro Gly Orn Asp Ile Leu Gln-NH_2)

The peptide was synthesized using 200.2 mg (0.050 mmol) of NovaSyn® TGR resin. The synthesis gave 286.6 mg of resin (99.2% yield). The cleavage yielded 52.6 mg of crude peptide (87.0% yield). The peptide was purified by preparative RP-HPLC using a C4 (PLG8_18) and a C18 column (PLG15_25) to give a 10.6 mg of pure peptide (96.9% purity). Retention time on analytical RP-HPLC was 27.4 minutes. The identity of the peptide was confirmed by MALDI-TOF mass spectrometry. Calculated for $C_{58}H_{103}N_{19}O_{17}$ [MH]+: 1338.785; observed: 1338.776. The concentration of the peptide for NMR analysis was 10.5 mM.

HPTDabAsp (Ac-Arg Thr Val Dab Val ^{D}Pro Gly Orn Asp Ile Leu Gln-NH_2)

The peptide was synthesized using 203.9 mg (0.051 mmol) of NovaSyn® TGR resin. The synthesis gave 293.0 mg of resin (99.2% yield). The cleavage yielded 55.3 mg of crude peptide (88.0% yield). The peptide was purified by preparative RP-HPLC using a C4 (PLG7_17) and a C18 column (PLG15_25) to give a 12.3 mg of pure peptide (96.1% purity). Retention time on analytical RP-HPLC was 27.4 minutes. The identity of the peptide was confirmed by MALDI-TOF mass spectrometry. Calculated for $C_{59}H_{105}N_{19}O_{17}$ [MH]+: 1352.801; observed: 1352.822. The concentration of the peptide for NMR analysis was 10.5 mM.

HPTOrnAsp (Ac-Arg Thr Val Orn Val ^{D}Pro Gly Orn Asp Ile Leu Gln-NH_2)

The peptide was synthesized using 204.8 mg (0.051 mmol) of NovaSyn® TGR resin. The synthesis gave 291.8 mg of resin (87.8% yield). The cleavage yielded 57.3 mg of crude peptide (75.8% yield). The peptide was purified by preparative RP-HPLC using a C4 column

(PLG7_17) to give a 18.9 mg of pure peptide (95.9% purity). Retention time on analytical RP-HPLC was 27.9 minutes. The identity of the peptide was confirmed by MALDI-TOF mass spectrometry. Calculated for $C_{60}H_{107}N_{19}O_{17}$ [MH]+: 1366.817; observed: 1367.091. The concentration of the peptide for NMR analysis was 9.9 mM.

HPTLysAsp (Ac-Arg Thr Val Lys Val ^{D}Pro Gly Orn Asp Ile Leu Gln-NH_2)

The peptide was synthesized using 200.0 mg (0.050 mmol) of NovaSyn® TGR resin. The synthesis gave 291.6 mg of resin (>99% yield). The cleavage yielded 57.0 mg of crude peptide (83.0% yield). The peptide was purified by preparative RP-HPLC using a C4 (PLG7_17) and a C18 column (PLG16_26) to give 7.4 mg of pure peptide (96.0% purity). Retention time on analytical RP-HPLC was 28.4 minutes. The identity of the peptide was confirmed by MALDI-TOF mass spectrometry. Calculated for $C_{61}H_{109}N_{19}O_{17}$ [MH]+: 1380.832; observed: 1380.873. The concentration of the peptide for NMR analysis was 11.0 mM.

HPTDapGlu (Ac-Arg Thr Val Dap Val ^{D}Pro Gly Orn Glu Ile Leu Gln-NH_2)

The peptide was synthesized using 207.3 mg (0.052 mmol) of NovaSyn® TGR resin. The synthesis gave 300.1 mg of resin (93.7% yield). The cleavage yielded 52.4 mg of crude peptide (80.0% yield). The peptide was purified by preparative RP-HPLC using a C4 (PLG6_16) and a C18 column (PLG15_25) to give 9.4 mg of pure peptide (97.2% purity). Retention time on analytical RP-HPLC was 26.7 minutes. The identity of the peptide was confirmed by MALDI-TOF mass spectrometry. Calculated for $C_{59}H_{105}N_{19}O_{17}$ [MH]+: 1352.801; observed: 1352.869. The concentration of the peptide for NMR analysis was 13.9 mM.

HPTDabGlu (Ac-Arg Thr Val Dab Val ^{D}Pro Gly Orn Glu Ile Leu Gln-NH_2)

The peptide was synthesized using 211.0 mg (0.053 mmol) of NovaSyn® TGR resin. The synthesis gave 292.4 mg of resin (98.1% yield). The cleavage yielded 48.6 mg of crude peptide (85.0% yield). The peptide was purified by preparative RP-HPLC using a C4 (PLG6_16) and a C18 column (PLG15_25) to give a 10.5 mg of pure peptide (95.7% purity). Retention time on analytical RP-HPLC was 26.9 minutes. The identity of peptide was confirmed by MALDI-TOF mass spectrometry. Calculated for $C_{60}H_{107}N_{19}O_{17}$ [MH]+: 1366.817; observed: 1366.929. The concentration of the peptide for NMR analysis was 15.4 mM.

HPTOrnGlu (Ac-Arg Thr Val Orn Val ^{D}Pro Gly Orn Glu Ile Leu Gln-NH_2)

The peptide was synthesized using 200.5 mg (0.050 mmol) of NovaSyn® TGR resin. The synthesis gave 293.4 mg of resin (99.6% yield). The cleavage yielded 52.8 mg of crude peptide (88.0% yield). The peptide was purified by preparative RP-HPLC using a C4

(PLG8_18) and a C18 column (PLG15_25) to give 9.1 mg of pure peptide (95.9% purity). Retention time on analytical RP-HPLC was 27.0 minutes. The identity of the peptide was confirmed by MALDI-TOF mass spectrometry. Calculated for $C_{61}H_{109}N_{19}O_{17}$ [MH]+: 1380.832; observed: 1381.082. The concentration of the peptide for NMR analysis was 13.2 mM.

HPTLysGlu (Ac-Arg Thr Val Lys Val ^{D}Pro Gly Orn Glu Ile Leu Gln-NH_2)

The peptide was synthesized using 207.2 mg (0.052 mmol) of NovaSyn® TGR resin. The synthesis gave 303.3 mg of resin (99.5% yield). The cleavage yielded 52.0 mg of crude peptide (76.0% yield). The peptide was purified by preparative RP-HPLC using a C4 (PLG5_15) and a C18 column (PLG15_25) to give a 14.1 mg of pure peptide (95.9% purity). Retention time on analytical RP-HPLC was 27.6 minutes. The identity of the peptide was confirmed by MALDI-TOF mass spectrometry. Calculated for $C_{62}H_{111}N_{19}O_{17}$ MH]+: 1394.848; observed: 1395.096. The concentration of the peptide for NMR analysis is 10.1 mM.

HPTDapAad (Ac-Arg Thr Val Dap Val ^{D}Pro Gly Orn Aad Ile Leu Gln-NH_2)

The peptide was synthesized using 200.9 mg (0.050 mmol) of NovaSyn® TGR resin. The synthesis gave 277.9 mg of resin (73.3% yield). The cleavage yielded 30.6 mg of crude peptide (51.0% yield). The peptide was purified by preparative RP-HPLC using Seppak® plus short tC_{18} cartridges (35% B) and a C18 column (PLG17_27) to 96.0% purity (15.1 mg). Retention time on analytical RP-HPLC was 27.2 minutes. The identity of the peptide was confirmed by MALDI-TOF mass spectrometry. Calculated for $C_{60}H_{107}N_{19}O_{17}$ [MH]+: 1366.817; observed: 1366.754. The concentration of the peptide for NMR analysis was 10.0 mM.

HPTDabAad (Ac-Arg Thr Val Dab Val ^{D}Pro Gly Orn Aad Ile Leu Gln-NH_2)

The peptide was synthesized using 200.5 mg (0.050 mmol) of NovaSyn® TGR resin. The synthesis gave 270.8 mg of resin (72.0% yield). The cleavage yielded 33.2 mg of crude peptide (56.0% yield). The peptide was purified by preparative RP-HPLC using Seppak® plus short tC_{18} cartridges (35% B) and a C18 column (PLG17_27) to 96.5% purity (11.3 mg). Retention time on analytical RP-HPLC was 27.6 minutes. The identity of the peptide was confirmed by MALDI-TOF mass spectrometry. Calculated for $C_{61}H_{109}N_{19}O_{17}$ [MH]+: 1380.832; observed: 1380.825. The concentration of the peptide for NMR analysis was 10.0 mM.

HPTOrnAad (Ac-Arg Thr Val Orn Val ^{D}Pro Gly Orn Aad Ile Leu Gln-NH_2)

The peptide was synthesized using 200.8 mg (0.050 mmol) of NovaSyn® TGR resin. The synthesis gave 271.5 mg of resin (71.8% yield). The cleavage yielded 37.3 mg of crude

peptide (62.0% yield). The peptide was purified by preparative RP-HPLC using Seppak® plus short tC_{18} cartridges (35% B) and a C4 column (PLG6_16) to give 8.0 mg of pure peptide (97.4% purity). Retention time on analytical RP-HPLC was 28.1 minutes. The identity of the peptide was confirmed by MALDI-TOF mass spectrometry. Calculated for $C_{62}H_{111}N_{19}O_{17}$ [MH]+: 1394.848; observed: 1395.037. The concentration of the peptide for NMR analysis was 10.4 mM.

HPTLysAad (Ac-Arg Thr Val Lys Val ^{D}Pro Gly Orn Aad Ile Leu Gln-NH_2)

The peptide was synthesized using 211.1 mg (0.053 mmol) of NovaSyn® TGR resin. The synthesis gave 301.9 mg of resin (86.3% yield). The cleavage yielded 58.2 mg of crude peptide (81.5% yield). The peptide was purified by preparative RP-HPLC using Seppak® plus short tC_{18} cartridges (40% B) and a C18 column (PLG17_27) to give 26.5 mg of pure peptide (97.4% purity). Retention time on analytical RP-HPLC was 27.4 minutes. The identity of the peptide was confirmed by MALDI-TOF mass spectrometry. Calculated for $C_{63}H_{113}N_{19}O_{17}$ [MH]+: 1408.863; observed: 1409.219. The concentration of the peptide for NMR analysis was 10.0 mM.

HPTUDapAsp (Ac-Arg Thr Val Dap Val ^{L}Pro Gly Orn Asp Ile Leu Gln-NH_2)

The peptide was synthesized using 202.8 mg (0.051 mmol) of NovaSyn® TGR resin. The synthesis gave 299.5 mg of resin (>99% yield). The cleavage yielded 72.7 mg of crude peptide (85.0% yield). The peptide was purified by preparative RP-HPLC using Seppak® plus short tC_{18} cartridges (25% B) and a C18 column (PLG16_26) to give 4.4 mg of pure peptide (96.9% purity). Retention time on analytical RP-HPLC was 27.9 minutes. The identity of the peptide was confirmed by MALDI-TOF mass spectrometry. Calculated for $C_{58}H_{103}N_{19}O_{17}$ [MH]+: 1338.785; observed: 1338.740. The concentration of the peptide for NMR analysis was 9.1 mM.

HPTUDabAsp (Ac-Arg Thr Val Dab Val ^{L}Pro Gly Orn Asp Ile Leu Gln-NH_2)

The peptide was synthesized using 207.6 mg (0.052 mmol) of NovaSyn TGR® resin. The synthesis gave 304.8 mg of resin (99.8% yield). The cleavage yielded 82.3 mg of crude peptide (96.0% yield). The peptide was purified by preparative RP-HPLC using Seppak® plus short tC_{18} cartridges (25% B) and a C18 column (PLG16_26) to give 4.3 mg of pure peptide (95.7% purity). Retention time on analytical RP-HPLC was 28.0 minutes. The identity of peptide was confirmed by MALDI-TOF mass spectrometry. Calculated for $C_{59}H_{105}N_{19}O_{17}$ [MH]+: 1352.801; observed: 1352.826. The concentration of the peptide for NMR analysis was 9.3 mM.

HPTUOrnAsp (Ac-Arg Thr Val Orn Val ^{L}Pro Gly Orn Asp Ile Leu Gln-NH_2)

The peptide was synthesized using 206.8 mg (0.052 mmol) of NovaSyn® TGR resin.

The synthesis gave 303.2 mg of resin (99.7% yield). The cleavage yielded 47.6 mg of crude peptide (56.0% yield). The peptide was purified by preparative RP-HPLC using Seppak® plus short tC_{18} cartridges (25% B) and a C18 column (PLG15_25) to give 3.8 mg of pure peptide (95.2% purity). Retention time on analytical RP-HPLC was 26.4 minutes. The identity of the peptide was confirmed by MALDI-TOF mass spectrometry. Calculated for $C_{60}H_{107}N_{19}O_{17}$ [MH]+: 1366.817; observed: 1366.801. The concentration of the peptide for NMR analysis is 9.3 mM.

HPTULysAsp (Ac-Arg Thr Val Lys Val ^{L}Pro Gly Orn Asp Ile Leu Gln-NH_2)

The peptide was synthesized using 210.8 mg (0.053 mmol) of NovaSyn® TGR resin. The synthesis gave 313.7 mg of resin (>99.9% yield). The cleavage yielded 60.9 mg of crude peptide (67.0% yield). The peptide was purified by preparative RP-HPLC using Seppak® plus short tC_{18} cartridges (25% B) and a C18 column (PLG16_26) to give 5.2 mg of pure peptide (95.0% purity). Retention time on analytical RP-HPLC was 27.7 minutes. The identity of the peptide was confirmed by MALDI-TOF mass spectrometry. Calculated for $C_{61}H_{109}N_{19}O_{17}$ [MH]+: 1380.833; observed: 1366.880. The concentration of the peptide for NMR analysis was 8.0 mM.

HPTUDapGlu (Ac-Arg Thr Val Dap Val ^{L}Pro Gly Orn Glu Ile Leu Gln-NH_2)

The peptide was synthesized using 213.8 mg (0.053 mmol) of NovaSyn® TGR resin. The synthesis gave 303.8 mg of resin (>99% yield). The cleavage yielded 72.7 mg of crude peptide (85.0% yield). The peptide was purified by preparative RP-HPLC using a C4 column (PLG5_15) to give 11.6 mg of pure peptide (95.7% purity). Retention time on analytical RP-HPLC was 26.1 minutes. The identity of the peptide was confirmed by MALDI-TOF mass spectrometry. Calculated for $C_{59}H_{105}N_{19}O_{17}$ [MH]+: 1352.801; observed :1353.004. The concentration of the peptide for NMR analysis was 10.2 mM.

HPTUDabGlu (Ac-Arg Thr Val Dab Val ^{L}Pro Gly Orn Glu Ile Leu Gln-NH_2)

The peptide was synthesized using 211.7 mg (0.053 mmol) of NovaSyn® TGR resin. The synthesis gave 308.1 mg of resin (>99% yield). The cleavage yielded 72.7 mg of crude peptide (85.0% yield). The peptide was purified by preparative RP-HPLC using a C4 column (PLG15_25) to give 12.0 mg of pure peptide (96.8% purity). Retention time on analytical RP-HPLC was 26.2 minutes. The identity of the peptide was confirmed by MALDI-TOF mass spectrometry. Calculated for $C_{60}H_{107}N_{19}O_{17}$ [MH]+: 1366.817; observed: 1366.909. The concentration of the peptide for NMR analysis was 9.7 mM.

HPTUOrnGlu (Ac-Arg Thr Val Orn Val ^{L}Pro Gly Orn Glu Ile Leu Gln-NH_2)

The peptide was synthesized using 213.1 mg (0.053 mmol) of NovaSyn® TGR resin. The synthesis gave 312.9 mg of resin (>99% yield). The cleavage yielded 72.7 mg of crude

peptide (85.0% yield). The peptide was purified by preparative RP-HPLC using a C4 column (PLG4_14) to give 15.4 mg of pure peptide (97.2% purity). Retention time on analytical RP-HPLC was 25.9 minutes. The identity of the peptide was confirmed by MALDI-TOF mass spectrometry. Calculated for $C_{61}H_{109}N_{19}O_{17}$ [MH]+: 1380.832; observed: 1380.929. The concentration of the peptide for NMR analysis was 13.2 mM.

HPTULysGlu (Ac-Arg Thr Val Lys Val ^{L}Pro Gly Orn Glu Ile Leu Gln-NH_2)

The peptide was synthesized using 210.0 mg (0.053 mmol) of NovaSyn® TGR resin. The synthesis gave 304.3 mg of resin (>99% peptide). The cleavage yielded 72.7 mg of crude peptide (85.0% yield). The peptide was purified by preparative RP-HPLC using a C4 column (PLG4_14) to give 18.6 mg of pure peptide (96.6% purity). Retention time on analytical RP-HPLC was 25.9 minutes. The identity of the peptide was confirmed by MALDI-TOF mass spectrometry. Calculated for $C_{62}H_{111}N_{19}O_{17}$ [MH]+: 1394.848; observed: 1394.983. The concentration of the peptide for NMR analysis was 13.2 mM.

HPTUDapAad (Ac-Arg Thr Val Dap Val ^{L}Pro Gly Orn Aad Ile Leu Gln-NH_2)

The peptide was synthesized using 202.8 mg (0.051 mmol) of NovaSyn® TGR resin. The synthesis gave 291.7 mg of resin (>99% yield). The cleavage yielded 72.7 mg of crude peptide (85.0% yield). The peptide was purified by preparative RP-HPLC using a C4 column (PLG4_14) to give 4.1 mg of pure peptide (95.7% purity). Retention time on analytical RP-HPLC was 26.2 minutes. The identity of the peptide was confirmed by MALDI-TOF mass spectrometry. Calculated for $C_{60}H_{107}N_{19}O_{17}$ [MH]+: 1366.817; observed: 1366.897. The concentration of the peptide for NMR analysis was 6.0 mM.

HPTUDabAad (Ac-Arg Thr Val Dab Val ^{L}Pro Gly Orn Aad Ile Leu Gln-NH_2)

The peptide was synthesized using 202.8 mg (0.051 mmol) of NovaSyn® TGR resin. The synthesis gave 299.5 mg of resin (>99% yield). The cleavage yielded 72.7 mg of crude peptide (85.0% yield). The peptide was purified by preparative RP-HPLC using a C4 column (PLG4_14) to give 7.8 mg of pure peptide (95.2% purity). Retention time on analytical RP-HPLC was 26.0 minutes. The identity of the peptide was confirmed by MALDI-TOF mass spectrometry. Calculated for $C_{61}H_{109}N_{19}O_{17}$ [MH]+: 1380.832; observed: 1380.958. The concentration of the peptide for NMR analysis was 11.0 mM.

HPTUOrnAad (Ac-Arg Thr Val Orn Val ^{L}Pro Gly Orn Aad Ile Leu Gln-NH_2)

The peptide was synthesized using 202.8 mg (0.051 mmol) of NovaSyn® TGR resin. The synthesis gave 299.5 mg of resin (>99% yield). The cleavage yielded 72.7 mg of crude peptide (85.0% yield). The peptide was purified by preparative RP-HPLC using a C4 (PLG3_13) to give 6.7 mg of pure peptide (95.8% purity). Retention time on analytical RP-HPLC was 26.0 minutes. The identity of the peptide was confirmed by MALDI-TOF

mass spectrometry. Calculated for $C_{62}H_{111}N_{19}O_{17}$ [MH]+: 1394.848; observed: 1395.985. The concentration of the peptide for NMR analysis was 9.6 mM.

HPTULysAad (Ac-Arg Thr Val Lys Val ^{L}Pro Gly Orn Aad Ile Leu Gln-NH_2)

The peptide was synthesized using 202.8 mg (0.051 mmol) of NovaSyn® TGR resin. The synthesis gave 299.5 mg of resin (>99% yield). The cleavage yielded 72.7 mg of crude peptide (85.0%). The peptide was purified by preparative RP-HPLC using a C4 (PLG4_14) to give 8.6 mg of pure peptide (96.7% purity). Retention time on analytical RP-HPLC was 26.2 minutes. The identity of the peptide was confirmed by MALDI-TOF mass spectrometry. Calculated for $C_{63}H_{113}N_{19}O_{17}$ [MH]+: 1408.863; observed: 1408.902. The concentration of the peptide for NMR analysis was 12.2 mM.

HPTFDapAsp (Ac-Cys Arg Thr Dap Val ^{D}Pro Gly Orn Asp Ile Leu Gln Cys-NH_2)

The peptide was synthesized using 204.9 mg (0.051 mmol) of NovaSyn® TGR resin. The synthesis gave 351.1 mg of resin (>99% yield). The cleavage yielded 98.7 mg of crude peptide (>99% yield). Retention time on analytical RP-HPLC was 29.8 minutes. The identity of the peptide was confirmed by MALDI-TOF mass spectrometry. Calculated for $C_{64}H_{113}N_{21}O_{19}S_2$ [MH]+: 1544.802; observed: 1544.820. The peptide was dissolved in 1 mM pH 8 phosphate, citrate, and borate buffer at a concentration of 0.1 mg/mL (~0.1 mM). Granulated charcoal was added to the peptide solution, using up to 10:1 (w/w) ratio of charcoal to peptide [3]. After stirring over air for 4 hours, the cyclized peptide was purified by preparative RP-HPLC using Seppak® plus short tC_{18} cartridges (25% B) and a C18 column (PLG16_26) to give 1.6 mg of pure peptide (95.5% purity). Retention time on analytical RP-HPLC was 26.7 minutes. The identity of the peptide was confirmed by MALDI-TOF mass spectrometry. Calculated for $C_{64}H_{111}N_{21}O_{19}S_2$ [MH]+: 1542.788; observed: 1542.886. The concentration of the peptide for NMR analysis was 2.1 mM.

HPTFDabAsp (Ac-Cys Arg Thr Dab Val ^{D}Pro Gly Orn Asp Ile Leu Gln Cys-NH_2)

The peptide was synthesized using 203.7 mg (0.051 mmol) of NovaSyn® TGR resin. The synthesis gave 330.1 mg of resin (99.6% yield). The cleavage yielded 93.3 mg of crude peptide (>99% yield). Retention time on analytical RP-HPLC was 30.1 minutes. The identity of the peptide was confirmed by MALDI-TOF mass spectrometry. Calculated for $C_{65}H_{115}N_{21}O_{19}S_2$ [MH]+: 1558.818; observed: 1558.629. The peptide was dissolved in 1 mM pH 8 phosphate, citrate, and borate buffer at a concentration of 0.1 mg/mL (~0.1 mM). Granulated charcoal was added to the peptide solution, using up to 10:1 (w/w) ratio of charcoal to peptide [3]. After stirring over air for 4 hours, the cyclized peptide was purified by preparative RP-HPLC using Seppak® plus short tC_{18} cartridges (25% B) and a C18 column (PLG16_26) to give 2.2 mg of pure peptide (95.3% purity). Retention time on analytical RP-HPLC was 26.8 minutes. The identity of the peptide was confirmed by

MALDI-TOF mass spectrometry. Calculated for $C_{65}H_{113}N_{21}O_{19}S_2$ [MH]+: 1556.804; observed: 1556.703. The concentration of the peptide for NMR analysis was 2.8 mM.

HPTFOrnAsp (Ac-Cys Arg Thr Orn Val ^{D}Pro Gly Orn Asp Ile Leu Gln Cys-NH_2)

The peptide was synthesized using 209.5 mg (0.052 mmol) of NovaSyn® TGR resin. The synthesis gave 325.5 mg of resin (84.4% yield). The cleavage yielded 88.1 mg of crude peptide (>99% yield). Retention time on analytical RP-HPLC was 30.1 minutes. The identity of the peptide was confirmed by MALDI-TOF mass spectrometry. Calculated for $C_{66}H_{117}N_{21}O_{19}S_2$ [MH]+: 1572.835; observed: 1573.083. The peptide was dissolved in 1 mM pH 8 phosphate, citrate, and borate buffer at a concentration of 1 mg/mL (~1 mM). Granulated charcoal was added to the peptide solution, using up to 10:1 (w/w) ratio of charcoal to peptide [3]. After stirring over air for 6 hours, the cyclized peptide was purified by preparative RP-HPLC using a C4 column (PLG8_18) to give 5.2 mg of pure peptide (95.9% purity). Retention time on analytical RP-HPLC was 27.2 minutes. The identity of the peptide was confirmed by MALDI-TOF mass spectrometry. Calculated for $C_{66}H_{115}N_{21}O_{19}S_2$ [MH]+: 1570.819; observed: 1571.151. The concentration of the peptide for NMR analysis was 5.9 mM.

HPTFLysAsp (Ac-Cys Arg Thr Lys Val ^{D}Pro Gly Orn Asp Ile Leu Gln Cys-NH_2)

The peptide was synthesized using 201.1 mg (0.050 mmol) of NovaSyn® TGR resin. The synthesis gave 329.9 mg of resin (99.8% yield). The cleavage yielded 94.6 mg of crude peptide (>99% yield). Retention time on analytical RP-HPLC was 30.6 minutes. The identity of the peptide was confirmed by MALDI-TOF mass spectrometry. Calculated for $C_{67}H_{119}N_{21}O_{19}S_2$ [MH]+: 1586.849; observed: 1586.813. The peptide was dissolved in 1 mM pH 8 phosphate, citrate, and borate buffer at a concentration of 0.1 mg/mL (~0.1 mM). Granulated charcoal was added to the peptide solution, using up to 10:1 (w/w) ratio of charcoal to peptide [3]. After stirring over air for 4 hours, the cyclized peptide was purified by preparative RP-HPLC using Seppak® plus short tC_{18} cartridges (25% B) and a C18 column (PLG16_26) to give 2.8 mg of pure peptide (95.9% purity). Retention time on analytical RP-HPLC was 26.9 minutes. The identity of the peptide was confirmed by MALDI-TOF mass spectrometry. Calculated for $C_{67}H_{117}N_{21}O_{19}S_2$ [MH]+: 1584.835; observed: 1584.713. The concentration of the peptide for NMR analysis was 3.5 mM.

HPTFDapGlu (Ac-Cys Arg Thr Dap Val ^{D}Pro Gly Orn Glu Ile Leu Gln Cys-NH_2)

The peptide was synthesized using 204.2 mg (0.051 mmol) of NovaSyn® TGR resin. The synthesis gave 315.4 mg of resin (98.7% yield). The cleavage yielded 79.5 mg of crude peptide (>99% yield). Retention time on analytical RP-HPLC was 29.2 minutes. The identity of the peptide was confirmed by MALDI-TOF mass spectrometry. Calculated for $C_{65}H_{115}N_{21}O_{19}S_2$ [MH]+: 1558.818; observed: 1558.841. The peptide was dissolved in 1 mM

pH 8 phosphate, citrate, and borate buffer at a concentration of 0.1 mg/mL (~0.1 mM). Granulated charcoal was added to the peptide solution, using up to 10:1 (w/w) ratio of charcoal to peptide [3]. After stirring over air for 4 hours, the cyclized peptide was purified by preparative RP-HPLC using Seppak® plus short tC_{18} cartridges (25% B) and a C18 column (PLG16_26) to give 7.4 mg of pure peptide (96.6% purity). Retention time on analytical RP-HPLC was 25.9 minutes. The identity of the peptide was confirmed by MALDI-TOF mass spectrometry. Calculated for $C_{65}H_{113}N_{21}O_{19}S_2$ [MH]+: 1556.804; observed: 1556.972. The concentration of the peptide for NMR analysis was 9.9 mM.

HPTFDabGlu (Ac-Cys Arg Thr Dab Val ^{D}Pro Gly Orn Glu Ile Leu Gln Cys-NH_2)

The peptide was synthesized using 204.2 mg (0.051 mmol) of NovaSyn® TGR resin. The synthesis gave 313.7 mg of resin (98.6% yield). The cleavage yielded 74.7 mg of crude peptide (>99% yield). Retention time on analytical RP-HPLC was 29.4 minutes. The peptide was dissolved in 1 mM pH 8 phosphate, citrate, and borate buffer at a concentration of 0.1 mg/mL (~0.1 mM). Granulated charcoal was added to the peptide solution, using up to 10:1 (w/w) ratio of charcoal to peptide [3]. After stirring over air for 4 hours, the cyclized peptide was purified by preparative RP-HPLC using Seppak® plus short tC_{18} cartridges (25% B) and a C18 column (PLG16_26) to give 4.0 mg of pure peptide (96.7% purity). Retention time on analytical RP-HPLC was 25.9 minutes. The identity of the peptide was confirmed by MALDI-TOF mass spectrometry. Calculated for $C_{66}H_{115}N_{21}O_{19}S_2$ [MH]+: 1570.819; observed: 1571.019. The concentration of the peptide for NMR analysis was 5.3 mM.

HPTFOrnGlu (Ac-Cys Arg Thr Val Orn Val ^{D}Pro Gly Orn Glu Ile Leu Gln Cys-NH_2)

The peptide was synthesized using 205.1 mg (0.049 mmol) of NovaSyn® TGR resin. The synthesis gave 312.7 mg of resin (82.8% yield). The cleavage yielded 91.4mg of crude peptide (>99.9% yield). Retention time on analytical RP-HPLC was 29.5 minutes. The identity of the peptide was confirmed by MALDI-TOF mass spectrometry. Calculated for $C_{67}H_{119}N_{21}O_{19}S_2$ $[MH]^+$: 1586.850; observed: 1586.892. The peptide was dissolved in 1 mM pH 8 phosphate, citrate, and borate buffer at a concentration of 0.1 mg/mL (~0.1 mM). Granulated charcoal was added to the peptide solution, using up to 10:1 (w/w) ratio of charcoal to peptide [3]. After stirring over air for 2 hours, the cyclized peptide was purified by using a preparative RP-HPLC C4 (PLG08_18) to 95.8% purity (5.1mg). Retention time on analytical RP-HPLC was 26.8 minutes. The identity of the peptide was confirmed by MALDI-TOF mass spectrometry. Calculated for $C_{67}H_{117}N_{21}O_{19}S_2$ $[MH]^+$: 1583.835; observed: 1585.075. The concentration of the peptide for NMR analysis was 6.4 mM.

HPTFLysGlu (Ac-Cys Arg Thr Lys Val ^{D}Pro Gly Orn Glu Ile Leu Gln Cys-NH_2)

The peptide was synthesized using 200.0 mg (0.050 mmol) of NovaSyn® TGR resin. The synthesis gave 349.8 mg of resin (>99% yield). The cleavage yielded 76.2 mg of crude peptide (70.0% yield). Retention time on analytical RP-HPLC was 30.1 minutes. The identity of the peptide was confirmed by MALDI-TOF mass spectrometry. Calculated for $C_{68}H_{121}N_{21}O_{19}S_2$ [MH]+: 1600.865; observed: 1600.896. The peptide was dissolved in 1 mM pH 8 phosphate, citrate, and borate buffer at a concentration of 0.1 mg/mL (~0.1 mM). Granulated charcoal was added to the peptide solution, using up to 10:1 (w/w) ratio of charcoal to peptide [3]. After stirring over air for 4 hours, the cyclized peptide was purified by preparative RP-HPLC using a C4 (PLG8_18) and a C18 column (PLG15_25) to give 2.0 mg of pure peptide (96.5% purity). Retention time on analytical RP-HPLC was 26.75 minutes. The identity of the peptide was confirmed by MALDI-TOF mass spectrometry. Calculated for $C_{68}H_{119}N_{21}O_{19}S_2$ [MH]+: 1598.851; observed: 1599.250. The concentration of the peptide for NMR analysis was 2.5 mM.

HPTFDapAad (Ac-Cys Arg Thr Dap Val ^{D}Pro Gly Orn Aad Ile Leu Gln Cys-NH_2)

The peptide was synthesized using 203.8 mg (0.051 mmol) of NovaSyn® TGR resin. The synthesis gave 333.1 mg of resin (99.8% yield). The cleavage yielded 84.6 mg of crude peptide (>99% yield). Retention time on analytical RP-HPLC was 29.9 minutes. The identity of the peptide was confirmed by MALDI-TOF mass spectrometry. Calculated for $C_{66}H_{117}N_{21}O_{19}S_2$ [MH]+: 1572.834; observed: 1572.528. The peptide was dissolved in 1 mM pH 8 phosphate, citrate, and borate buffer at a concentration of 0.1 mg/mL (~0.1 mM). Granulated charcoal was added to the peptide solution, using up to 10:1 (w/w) ratio of charcoal to peptide [3]. After stirring over air for 4 hours, the cyclized peptide was purified by preparative RP-HPLC using Seppak® plus short tC_{18} cartridges (25% B) and a C18 column (PLG16_26) to give 7.3 mg of pure peptide (96.5% purity). Retention time on analytical RP-HPLC was 27.0 minutes. The identity of the peptide was confirmed by MALDI-TOF mass spectrometry. Calculated for $C_{66}H_{115}N_{21}O_{19}S_2$ [MH]+: 1570.819; observed: 1570.923. The concentration of the peptide for NMR analysis was 9.3 mM.

HPTFDabAad (Ac-Cys Arg Thr Dap Val ^{D}Pro Gly Orn Aad Ile Leu Gln Cys-NH_2)

The peptide was synthesized using 201.8 mg (0.050 mmol) of NovaSyn® TGR resin. The synthesis gave 322.4 mg of resin (99.3% yield). The cleavage yielded 80.7 mg of crude peptide (>99% yield). Retention time on analytical RP-HPLC was 29.8 minutes. The identity of the peptide was confirmed by MALDI-TOF mass spectrometry. Calculated for $C_{67}H_{119}N_{21}O_{19}S_2$ [MH]+: 1586.849; observed: 1586.415. The peptide was dissolved in 1 mM pH 8 phosphate, citrate, and borate buffer at a concentration of 0.1 mg/mL (~0.1 mM). Granulated charcoal was added to the peptide solution, using up to 10:1 (w/w) ratio of charcoal to peptide [3]. After stirring over air for 4 hours, the cyclized peptide was purified

by preparative RP-HPLC using using Seppak® plus short tC_{18} cartridges (25% B) and C18 column and (PLG16_26) to give 6.6 mg of pure peptide (96.7% purity). Retention time on analytical RP-HPLC was 27.1 minutes. The identity of the peptide was confirmed by MALDI-TOF mass spectrometry. Calculated for $C_{67}H_{117}N_{21}O_{19}S_2$ [MH]+: 1584.835; observed: 1585.058. The concentration of the peptide for NMR analysis was 8.3 mM.

HPTFOrnAad (Ac-Cys Arg Thr Orn Val ^{D}Pro Gly Orn Aad Ile Leu Gln Cys-NH_2)

The peptide was synthesized using 201.6 mg (0.050 mmol) of NovaSyn® TGR resin. The synthesis gave 307.7 mg of resin (80.0% yield). The cleavage yielded 69.1 mg of crude peptide (92.0% yield). Retention time on analytical RP-HPLC was 31.4 minutes. The identity of the peptide was confirmed by MALDI-TOF mass spectrometry. Calculated for $C_{68}H_{121}N_{21}O_{19}S_2$ [MH]+: 1600.865; observed: 1601.087. The peptide was dissolved in 1 mM pH 8 phosphate, citrate, and borate buffer at a concentration of 1 mg/mL (~1 mM). Granulated charcoal was added to the peptide solution, using up to 10:1 (w/w) ratio of charcoal to peptide [3]. After stirring over air for 4 hours, the cyclized peptide was purified by preparative RP-HPLC using Seppak® plus short tC_{18} cartridges (35% B) and a C4 column (PLG8_18) to give 3.1 mg of pure peptide (96.8% purity). Retention time on analytical RP-HPLC was 28.4 minutes. The identity of the peptide was confirmed by MALDI-TOF mass spectrometry. Calculated for $C_{68}H_{119}N_{21}O_{19}S_2$ [MH]+: 1598.851; observed: 1599.270. The concentration of the peptide for NMR analysis was 3.5 mM.

HPTFLysAad (Ac-Cys Arg Thr Lys Val ^{D}Pro Gly Orn Aad Ile Leu Gln Cys-NH_2)

The peptide was synthesized using 205.4 mg (0.051 mmol) of NovaSyn® TGR resin. The synthesis gave 333.4 mg of resin (99.5% yield). The cleavage yielded 101.6 mg of crude peptide (>99% yield). Retention time on analytical RP-HPLC was 30.5 minutes. The identity of the peptide was confirmed by MALDI-TOF mass spectrometry. Calculated for $C_{69}H_{123}N_{21}O_{19}S_2$ [MH]+: 1614.881; observed: 1614.778. The peptide was dissolved in 1 mM pH 8 phosphate, citrate, and borate buffer at a concentration of 0.1 mg/mL (~0.1 mM). Granulated charcoal was added to the peptide solution, using up to 10:1 (w/w) ratio of charcoal to peptide [3]. After stirring over air for 4 hours, the cyclized peptide was purified by preparative RP-HPLC using Seppak® plus short tC_{18} cartridges (25% B) and a C18 column (PLG16_26) to give 2.7 mg of pure peptide (96.9% purity). Retention time on analytical RP-HPLC was 26.9 minutes. The identity of the peptide was confirmed by MALDI-TOF mass spectrometry. Calculated for $C_{69}H_{121}N_{21}O_{19}S_2$ [MH]+: 1612.866; observed: 1612.696. The concentration of the peptide for NMR analysis was 2.0 mM.

References

1. Atherton, E.; Fox, H.; Harkiss, D.; Logan, C. J.; Sheppard, R. C.; Williams, B. J., A mild procedure for solid phase peptide synthesis: use of fluorenylmethoxycarbonylamino-acids. *J. Chem. Soc., Chem. Commun.* **1978**, 537-539.
2. Fields, G. B.; Noble, R. L., Solid phase peptide synthesis utilizing 9-fluorenylmethoxycarbonyl amino acids. *Int. J. Pept. Protein Res.* **1990,** 35, 161-214.
3. Volkmer-Engert, R.; Landgraf, C.; Schneider-Mergener, J., Charcoal surface-assisted catalysis of intramolecular disulfide bond formation in peptides. *J. Pept. Res.* **1998,** 51, 365-369.
